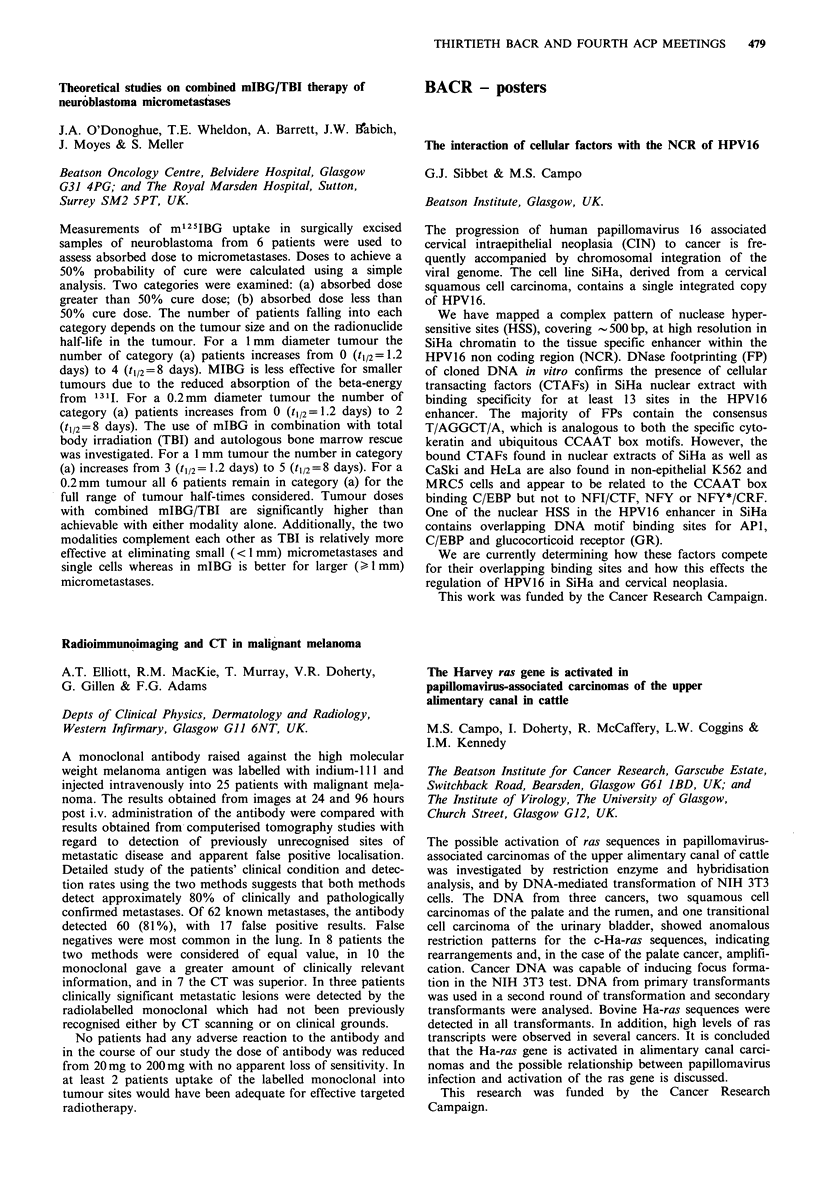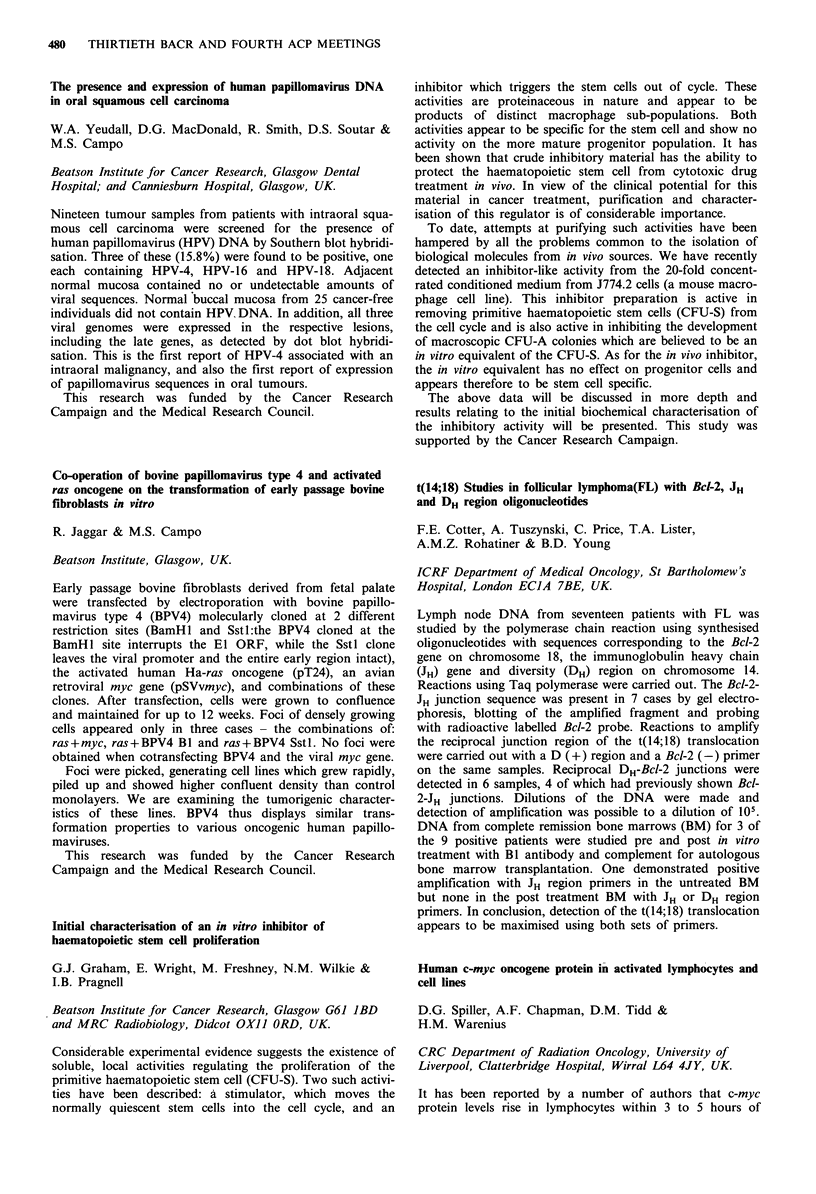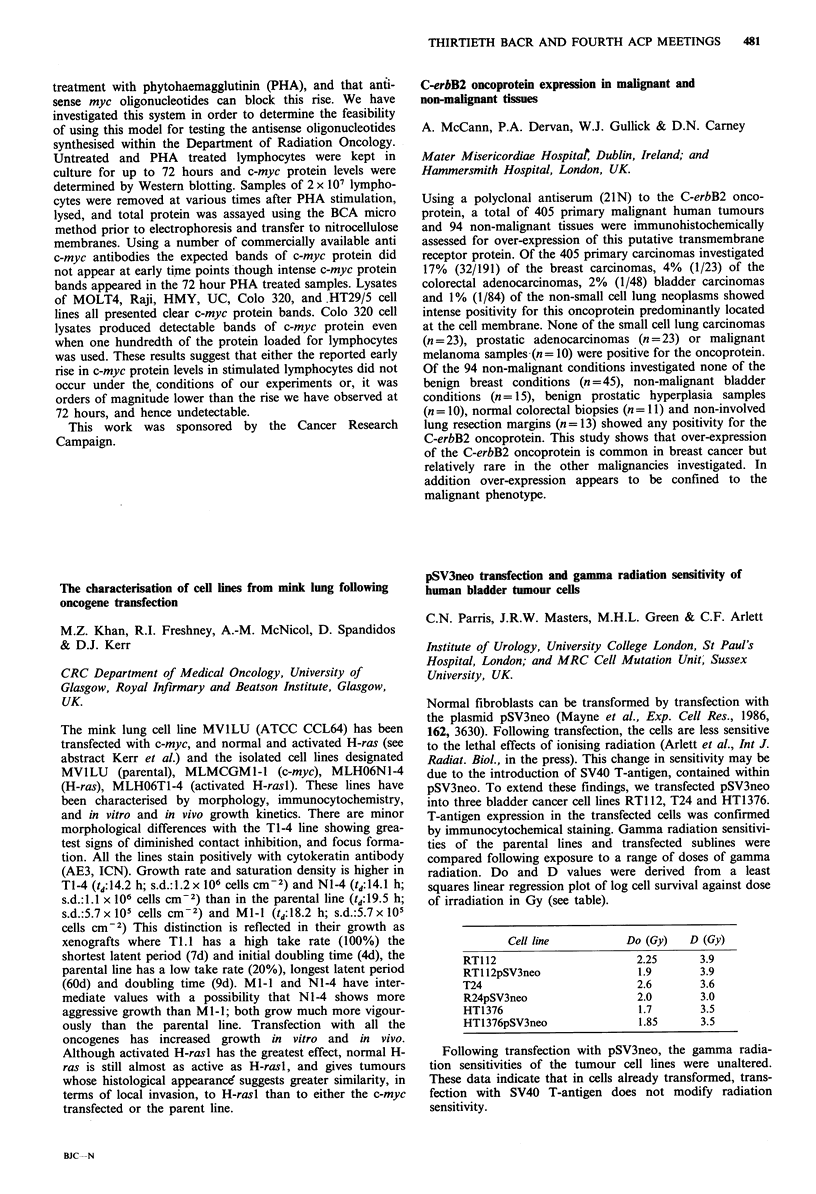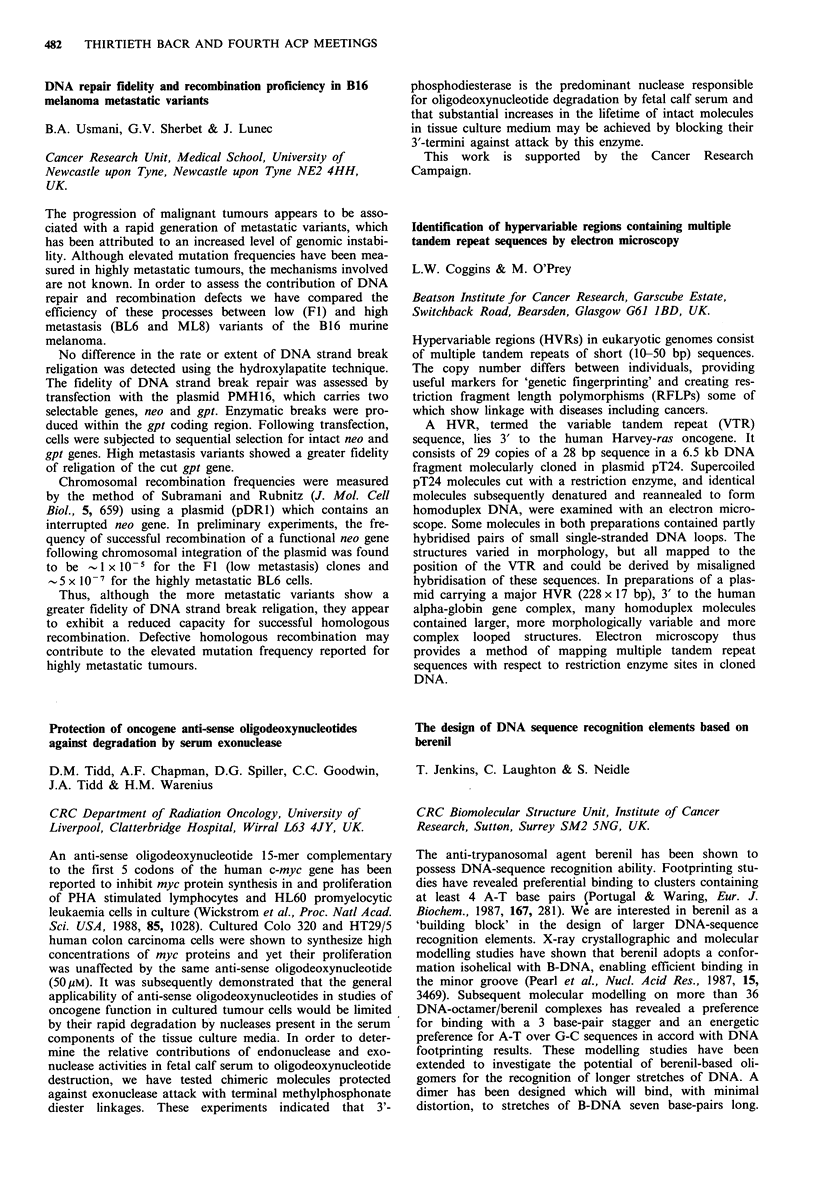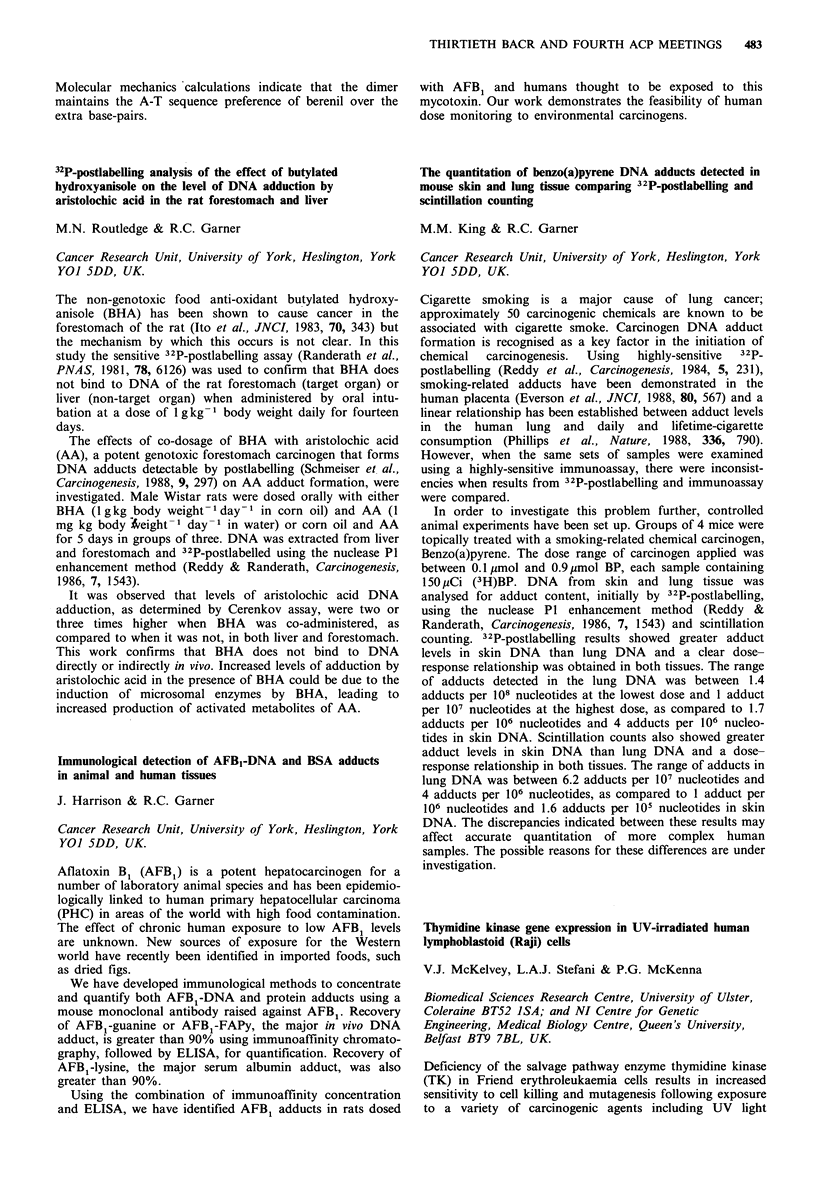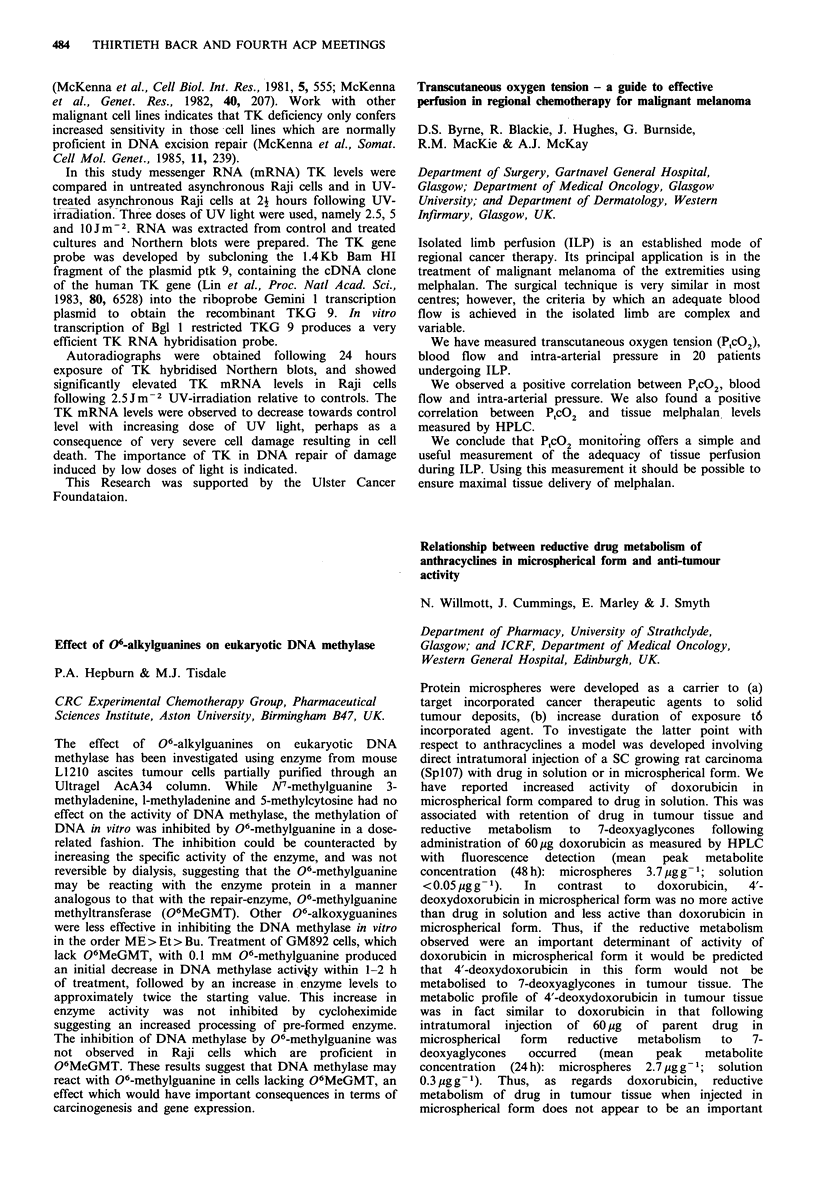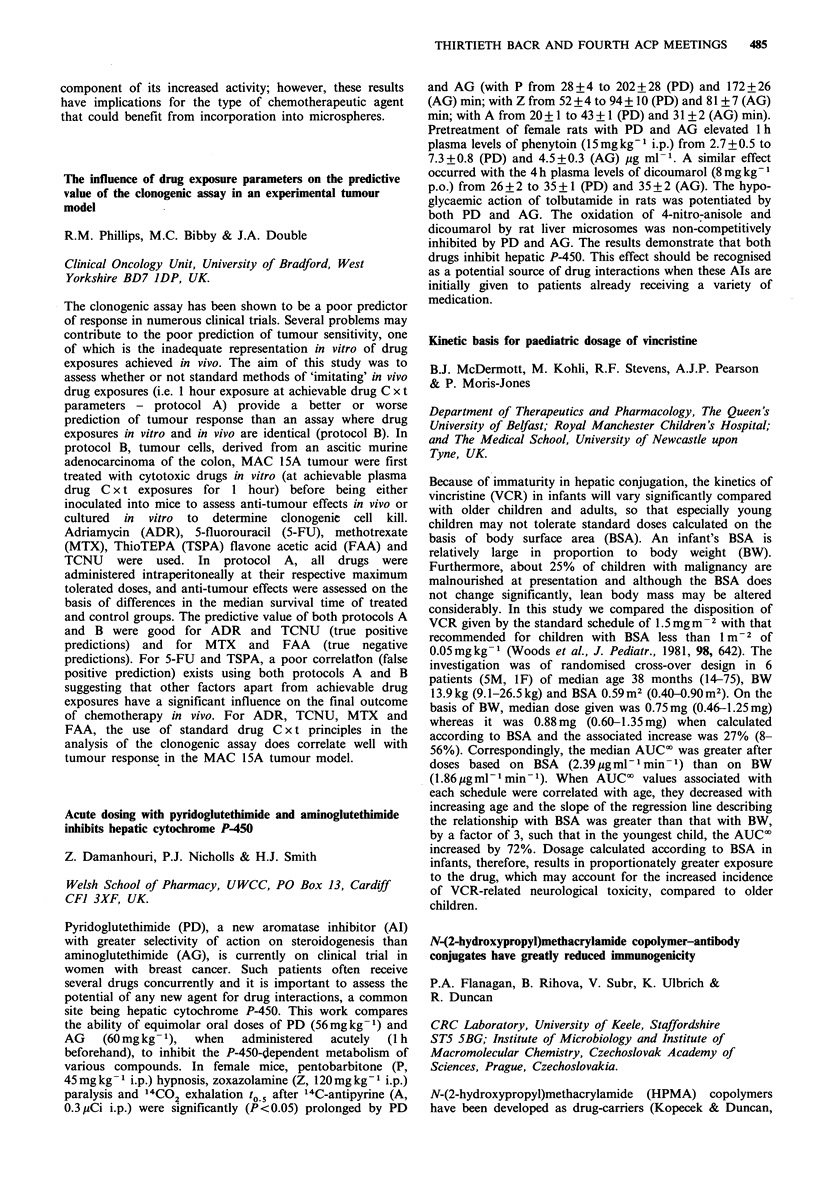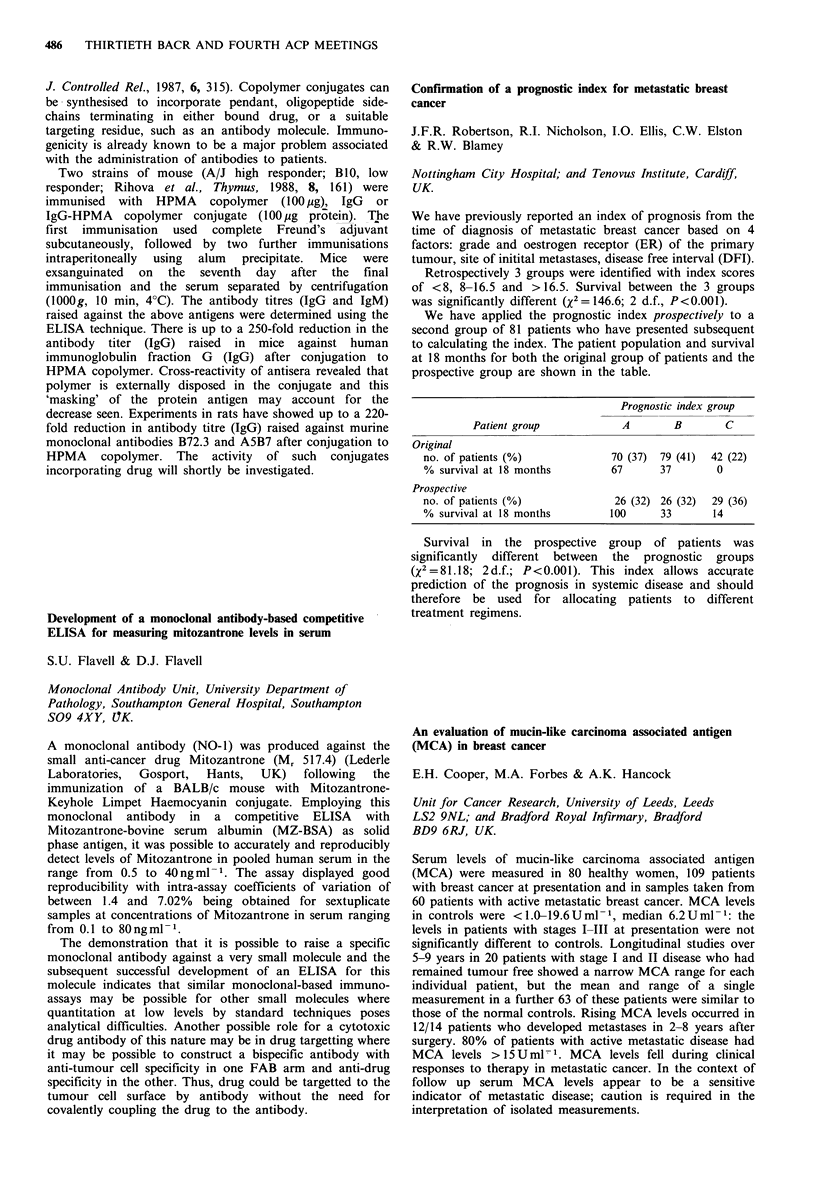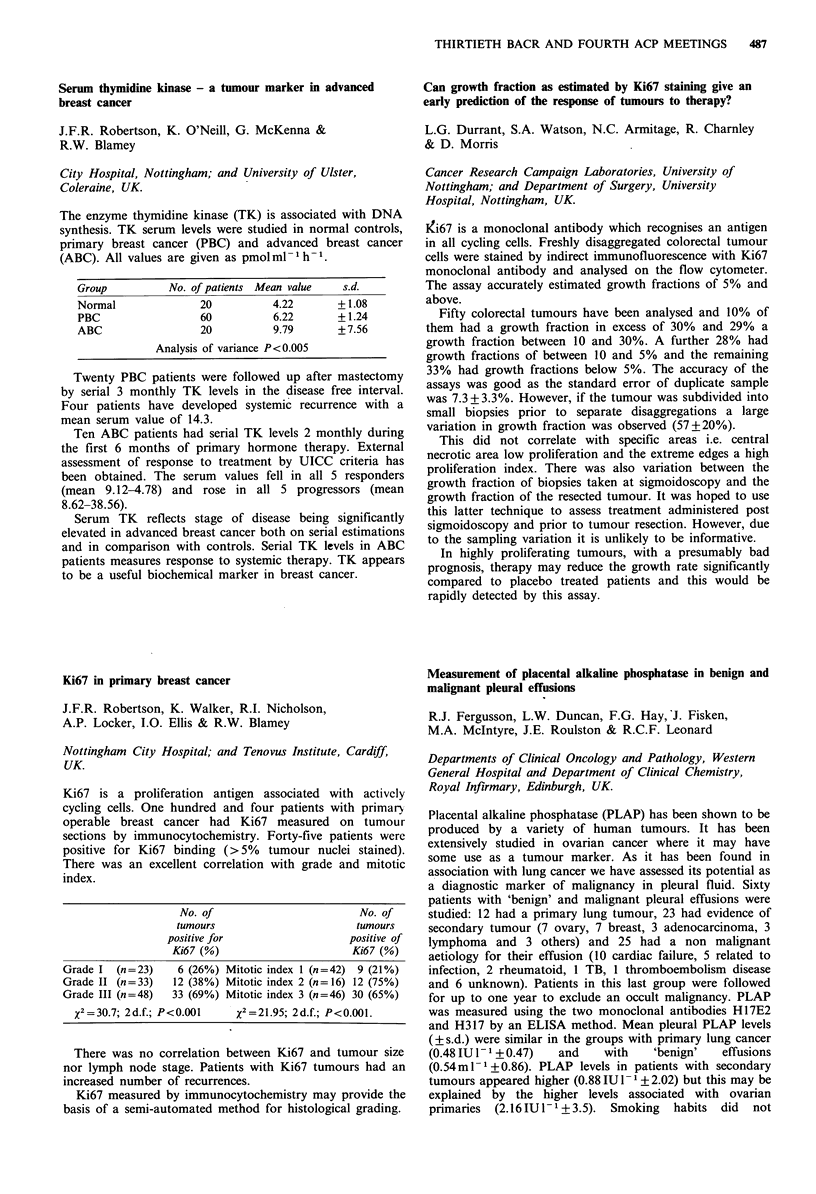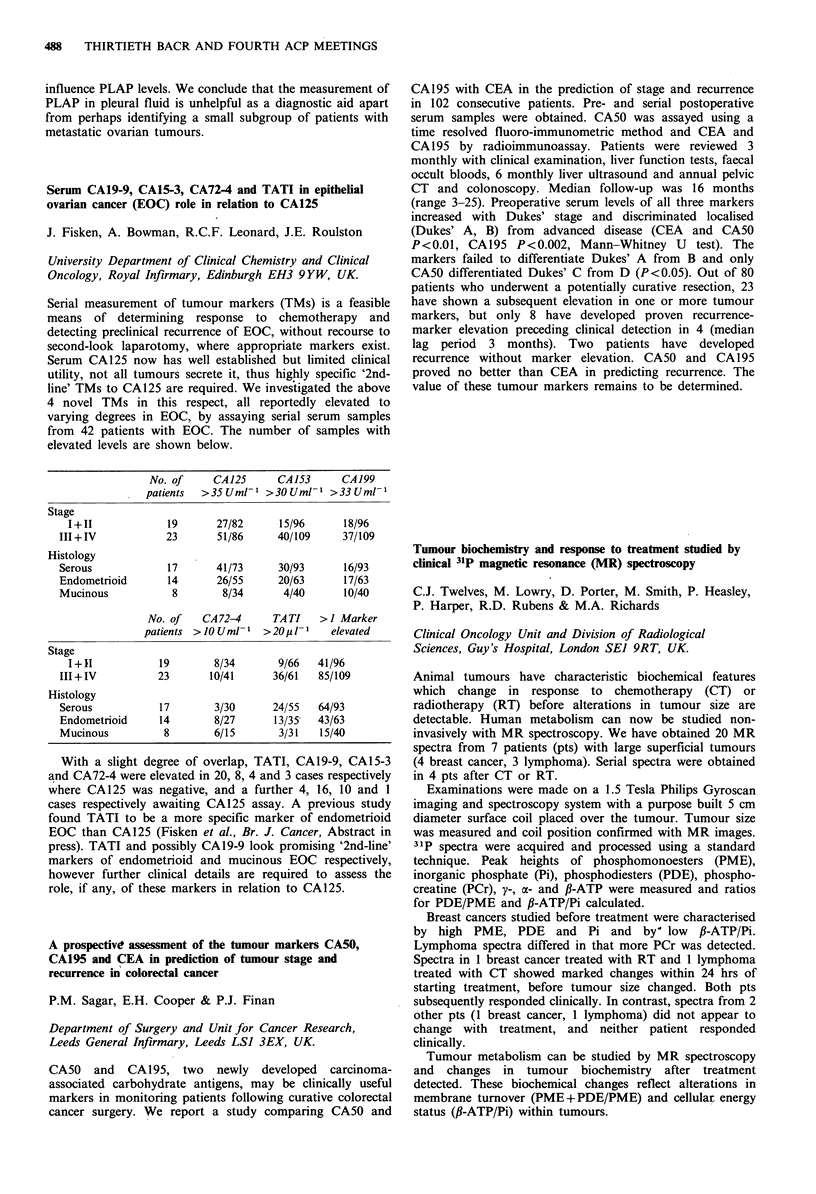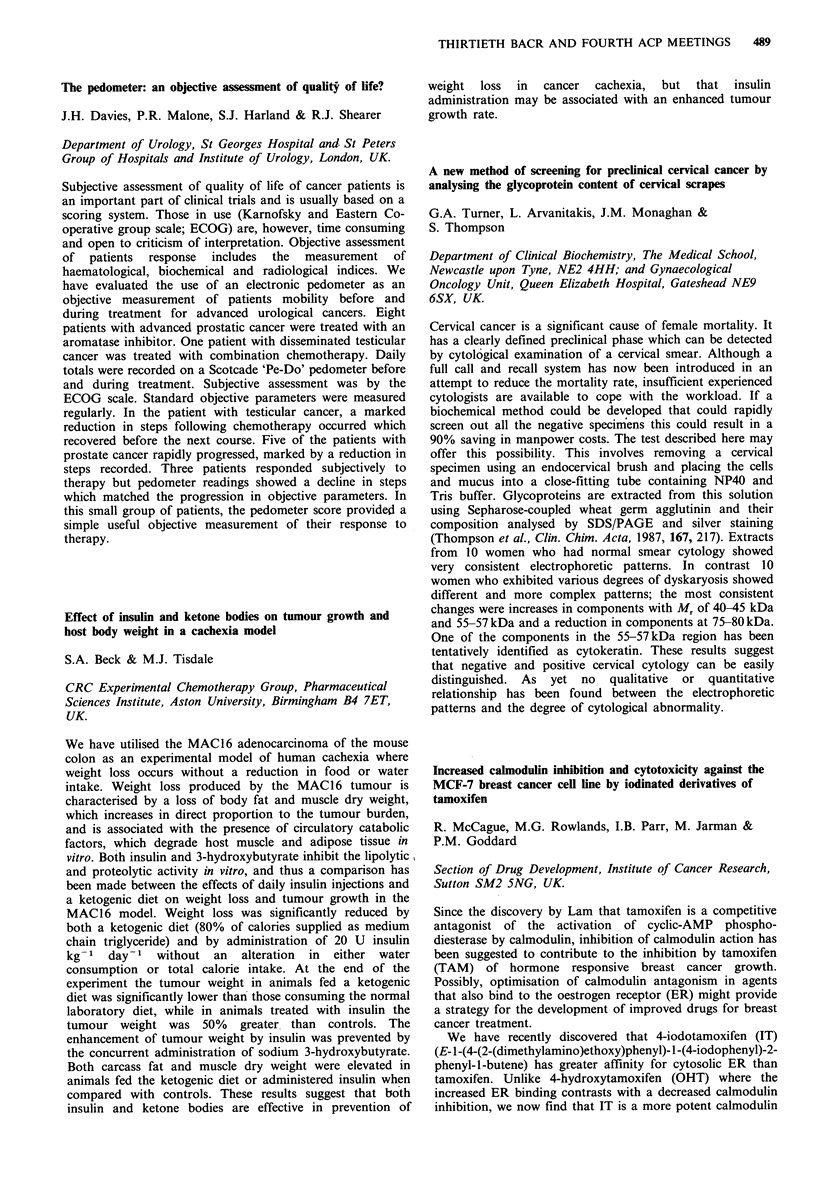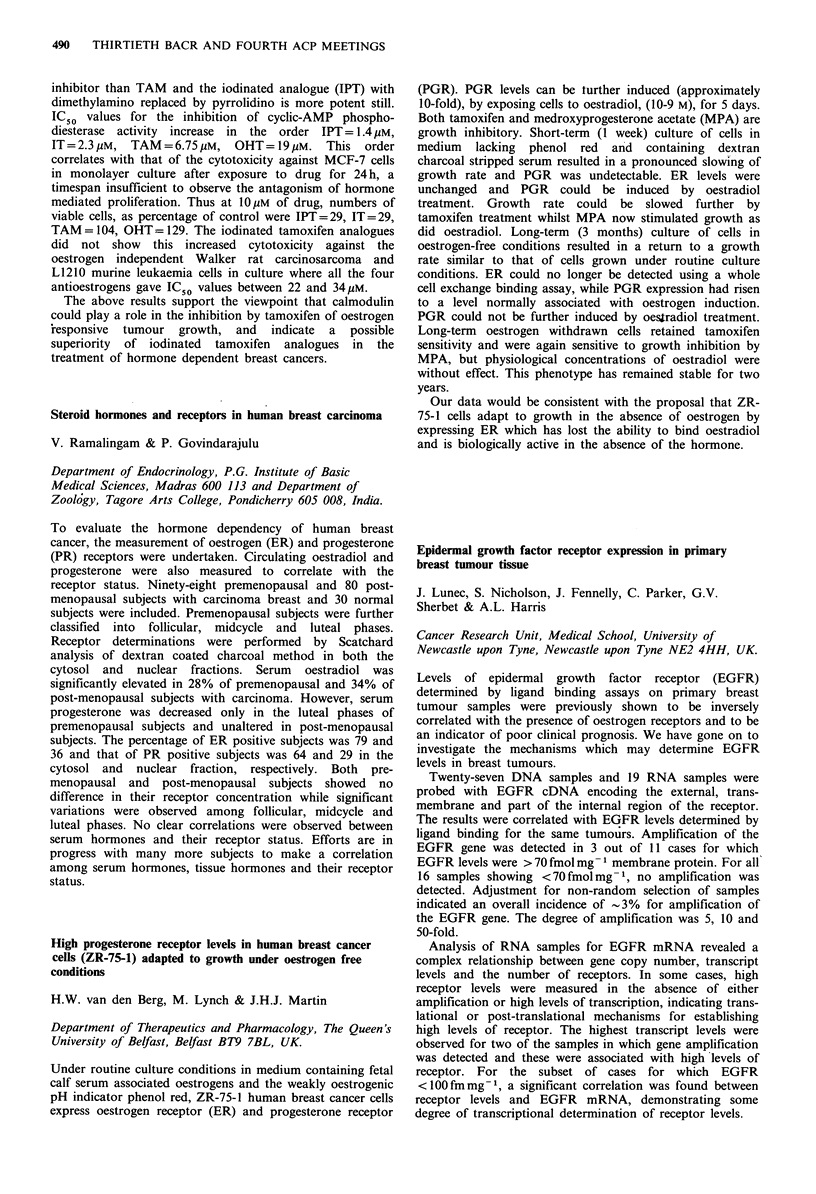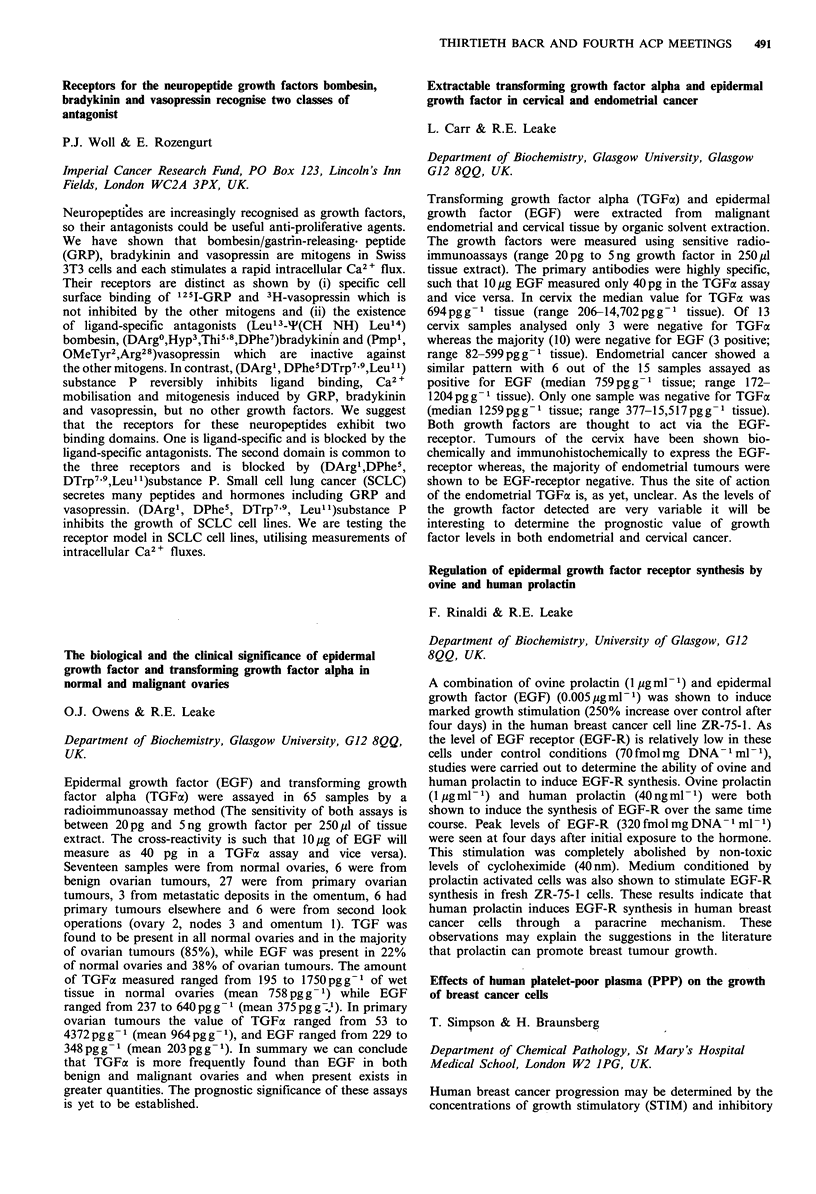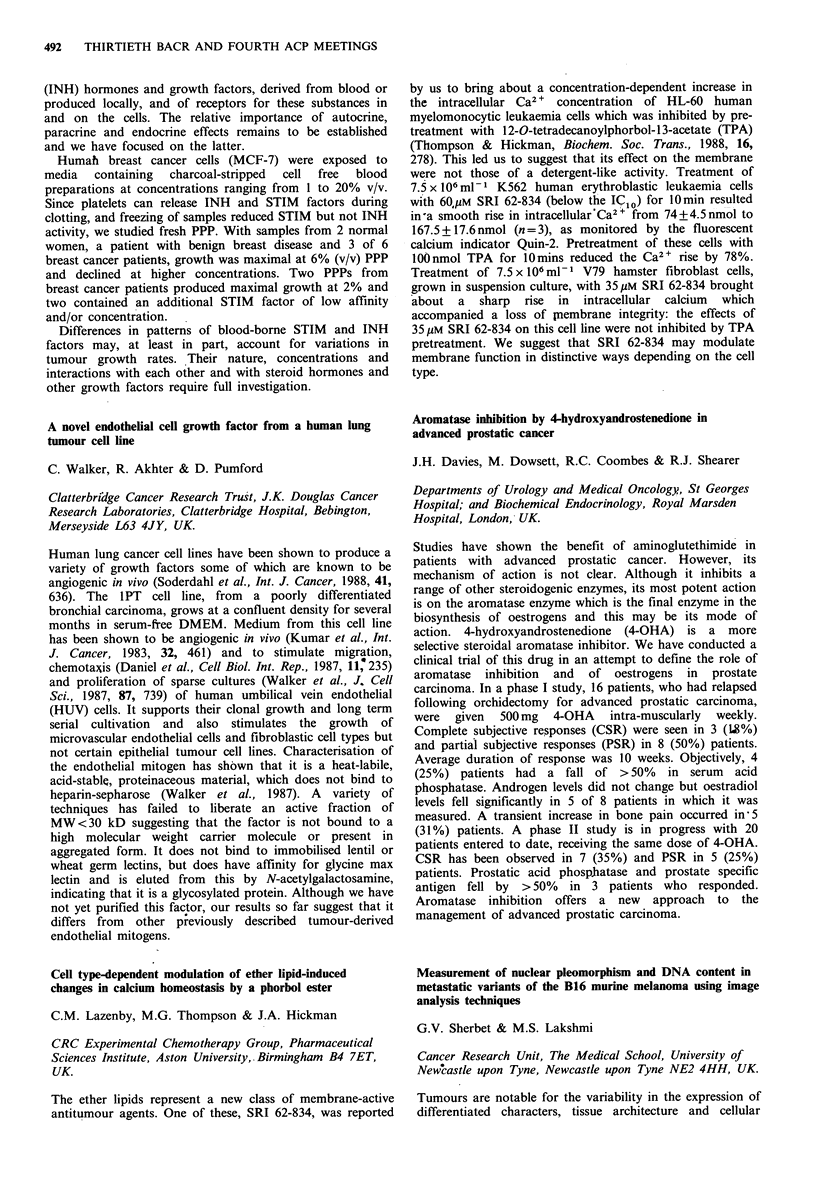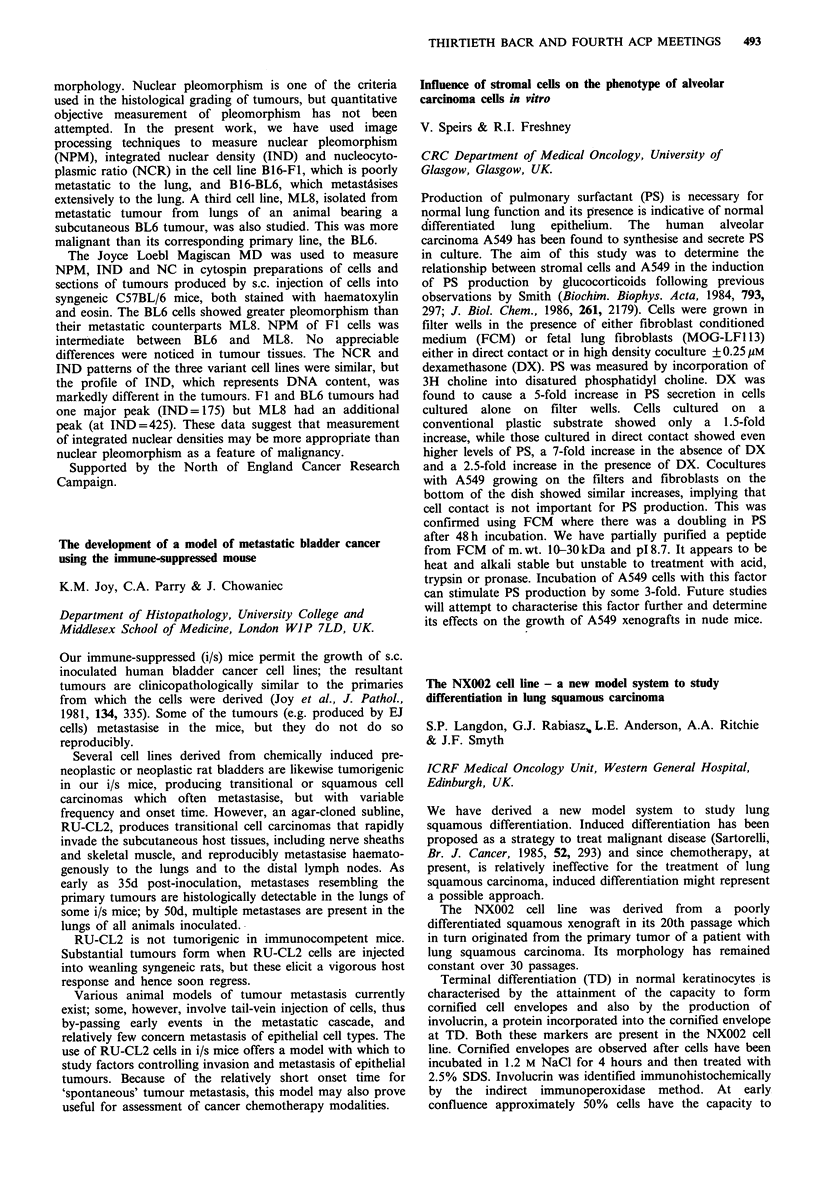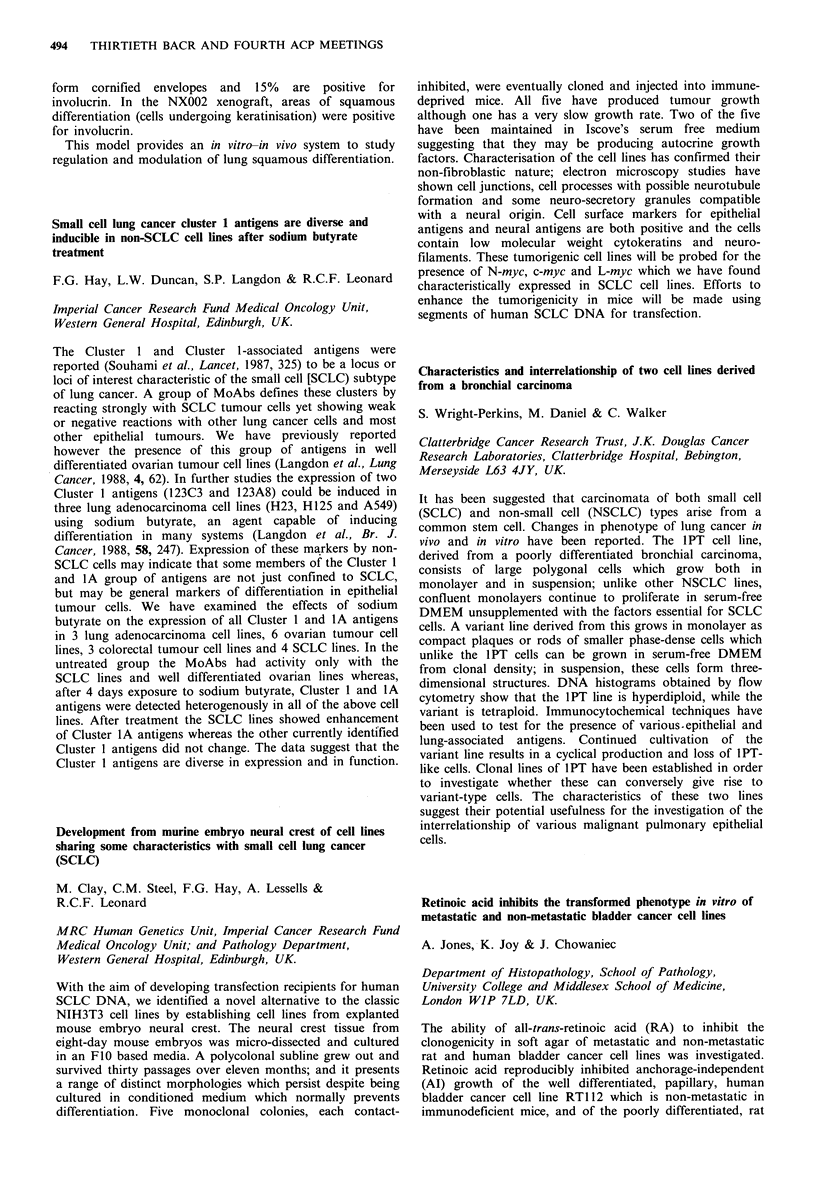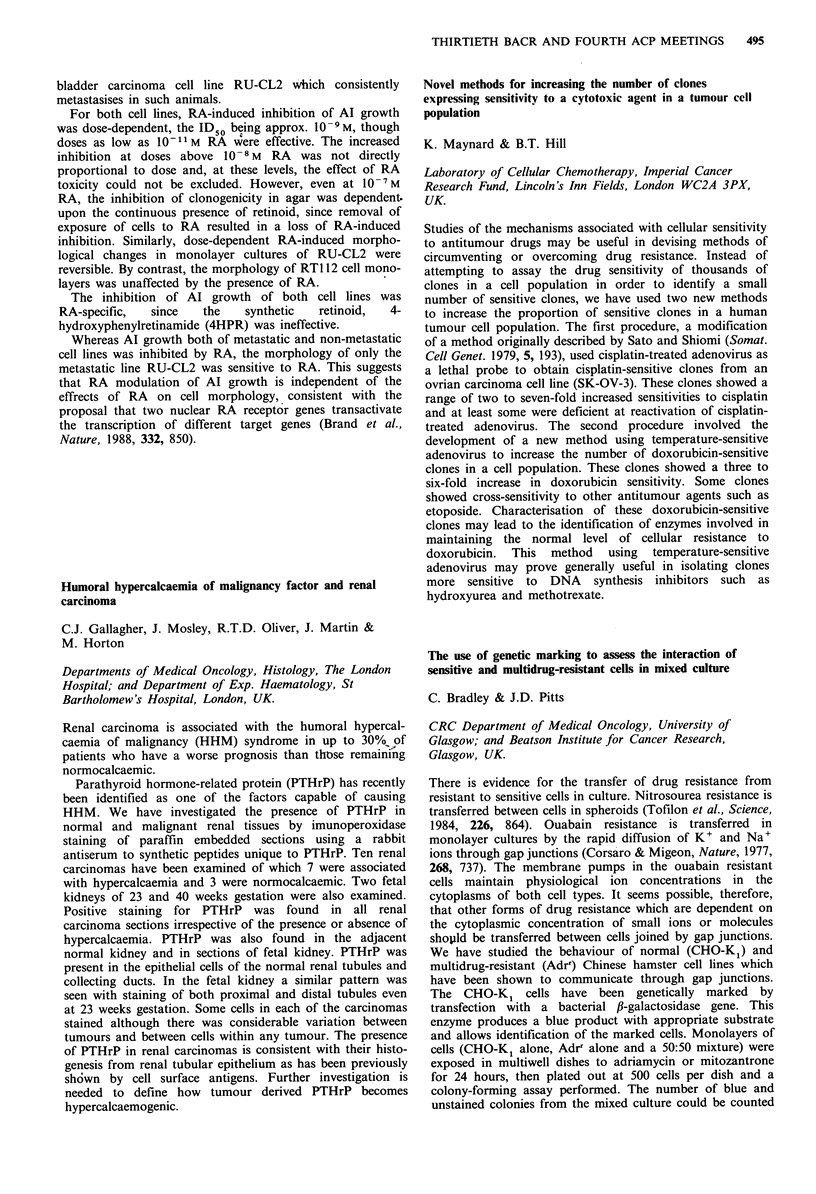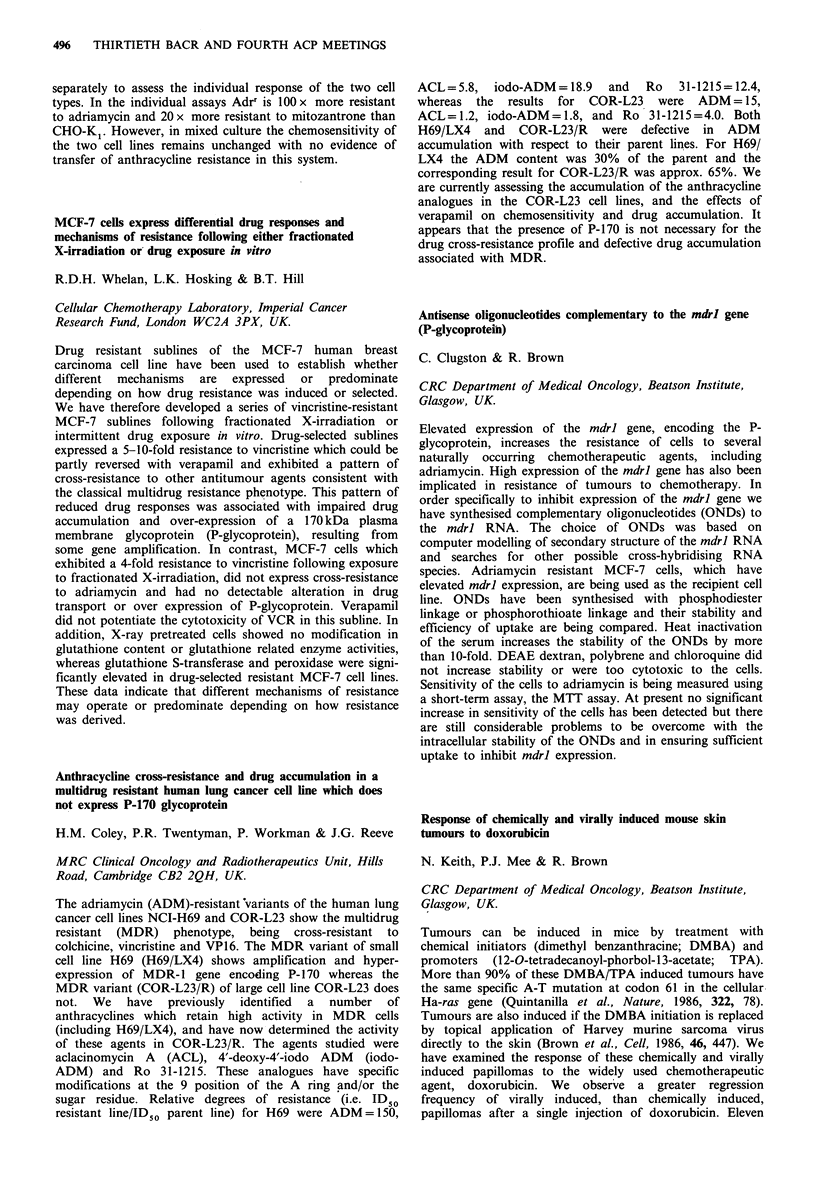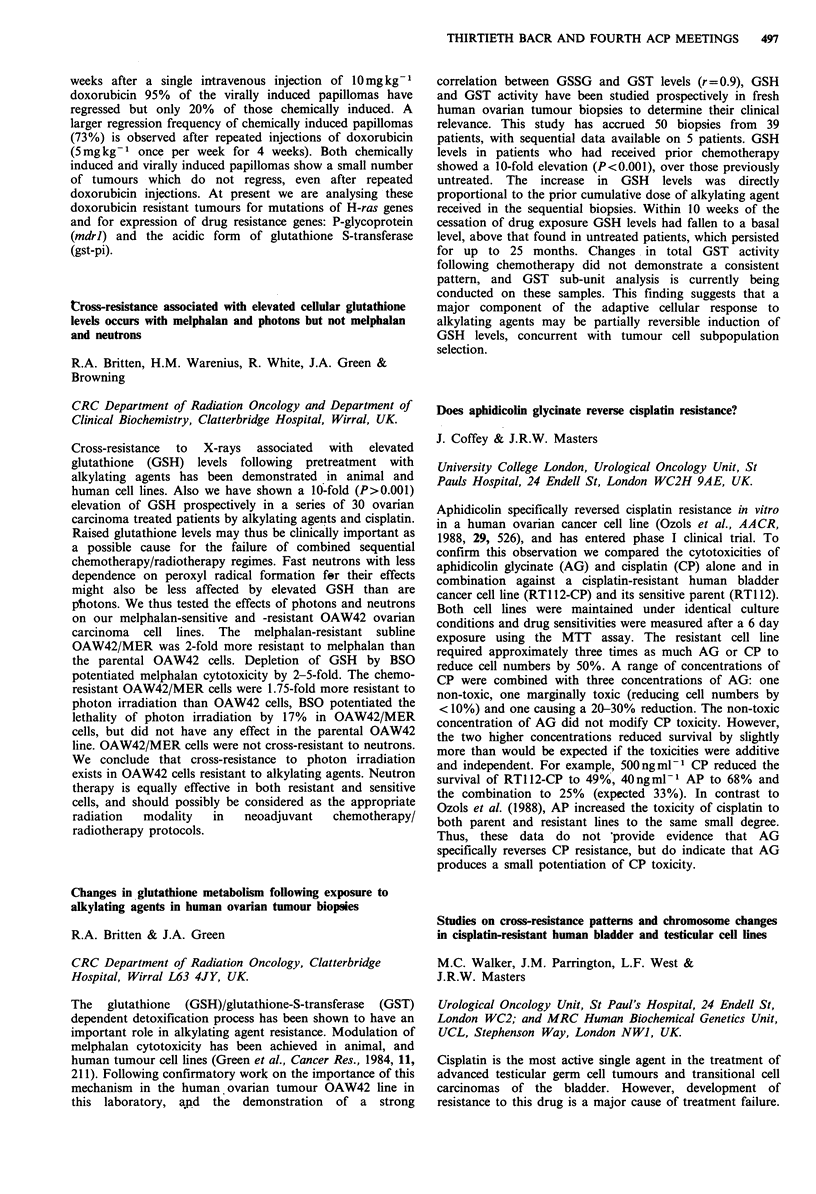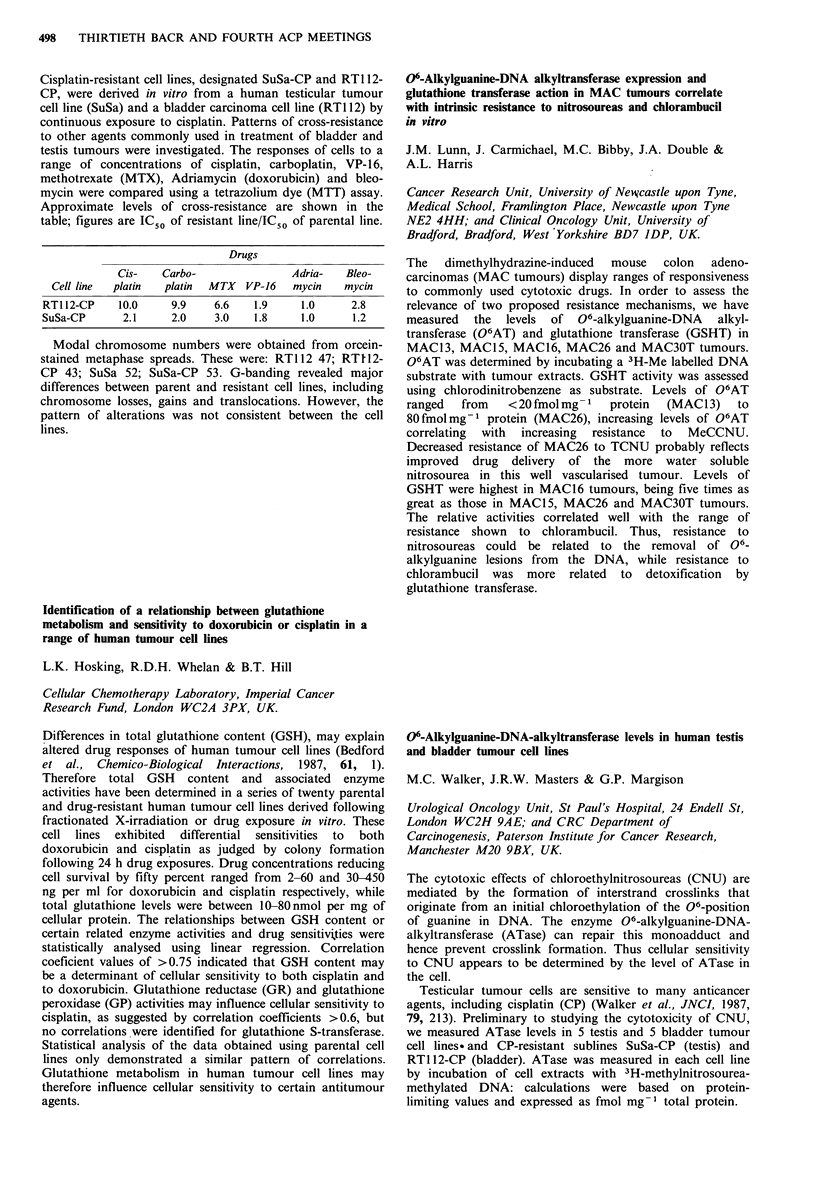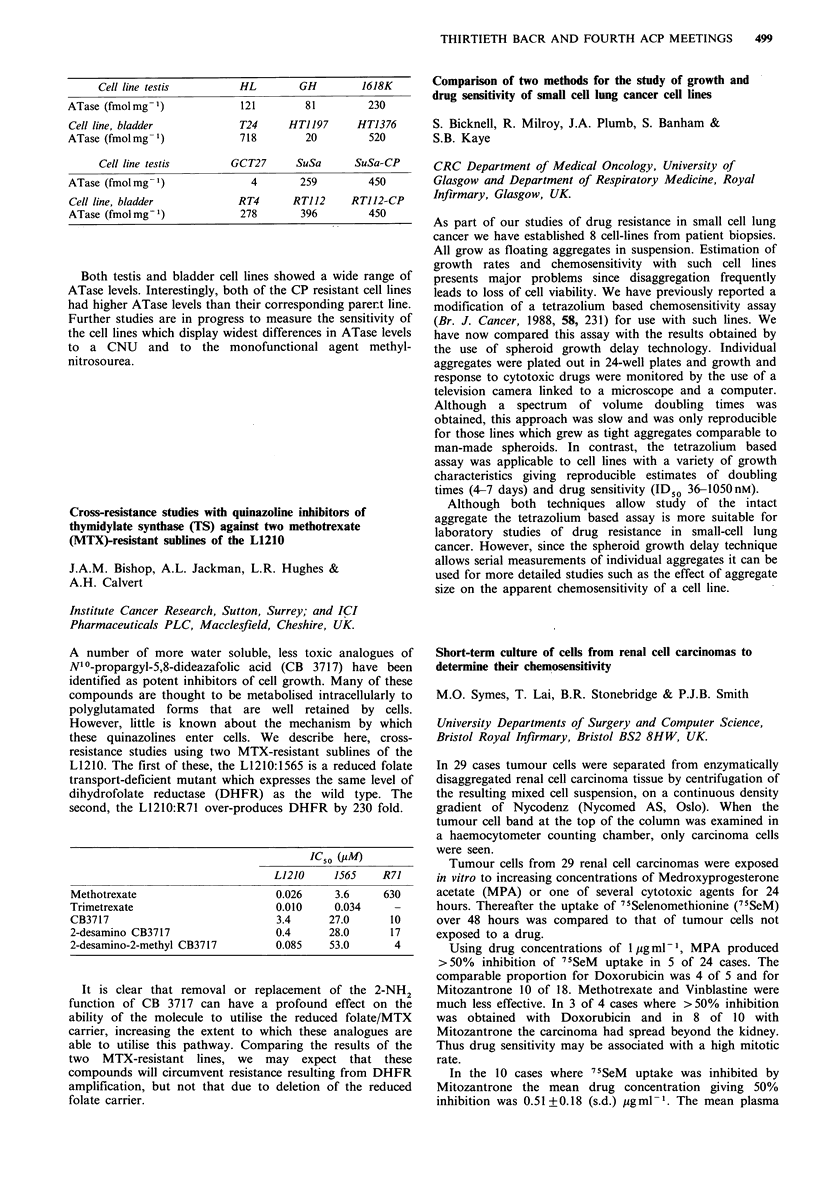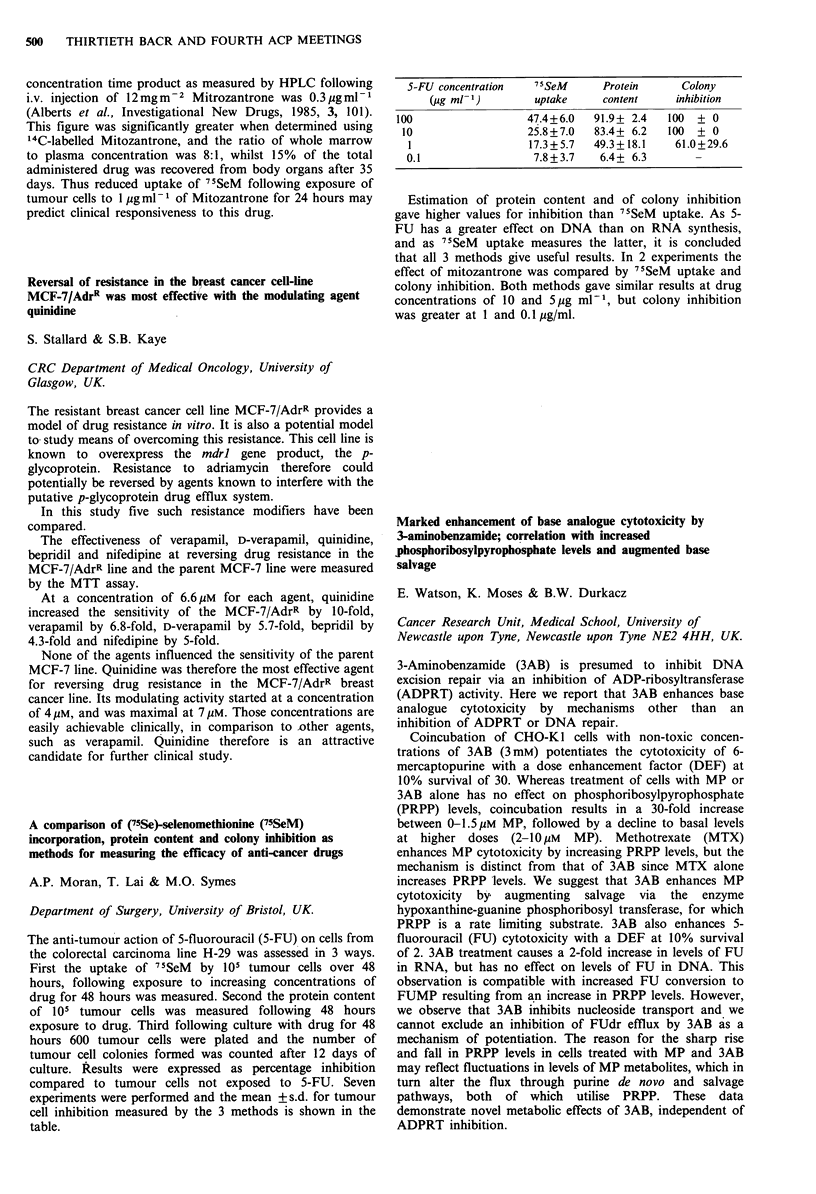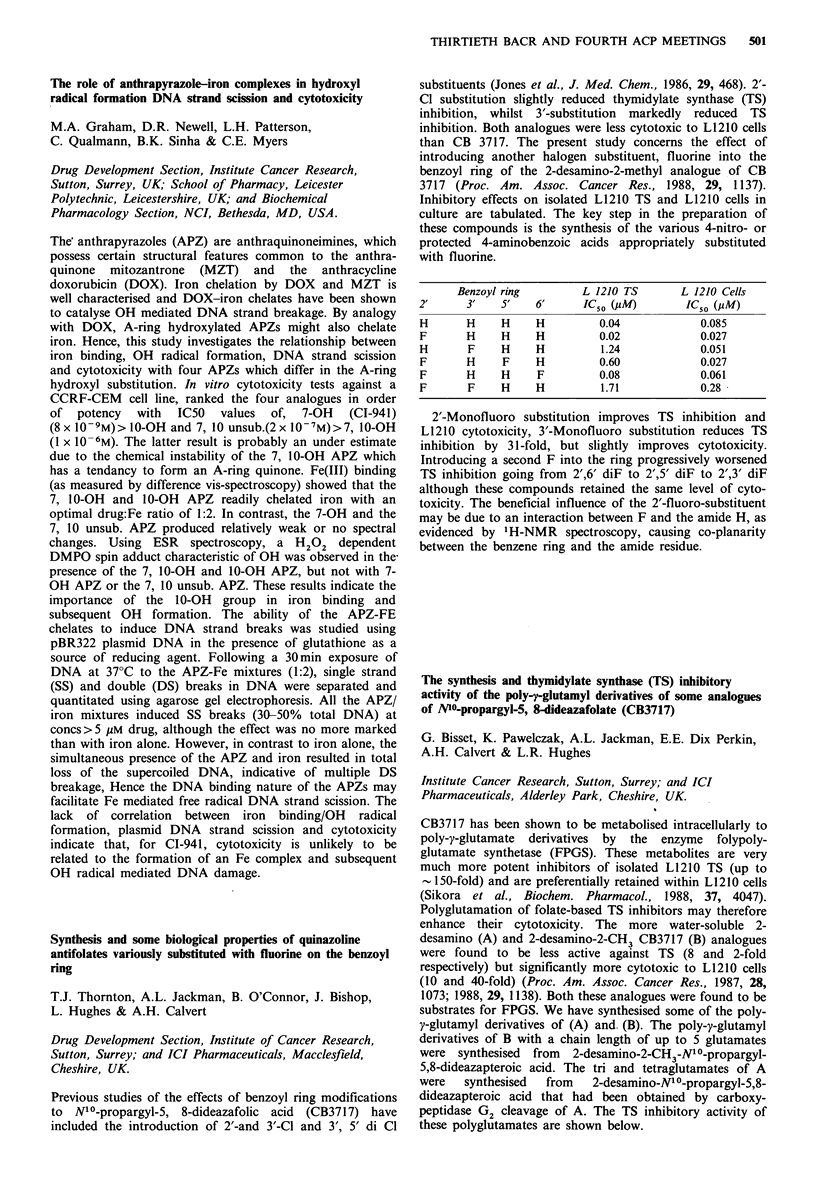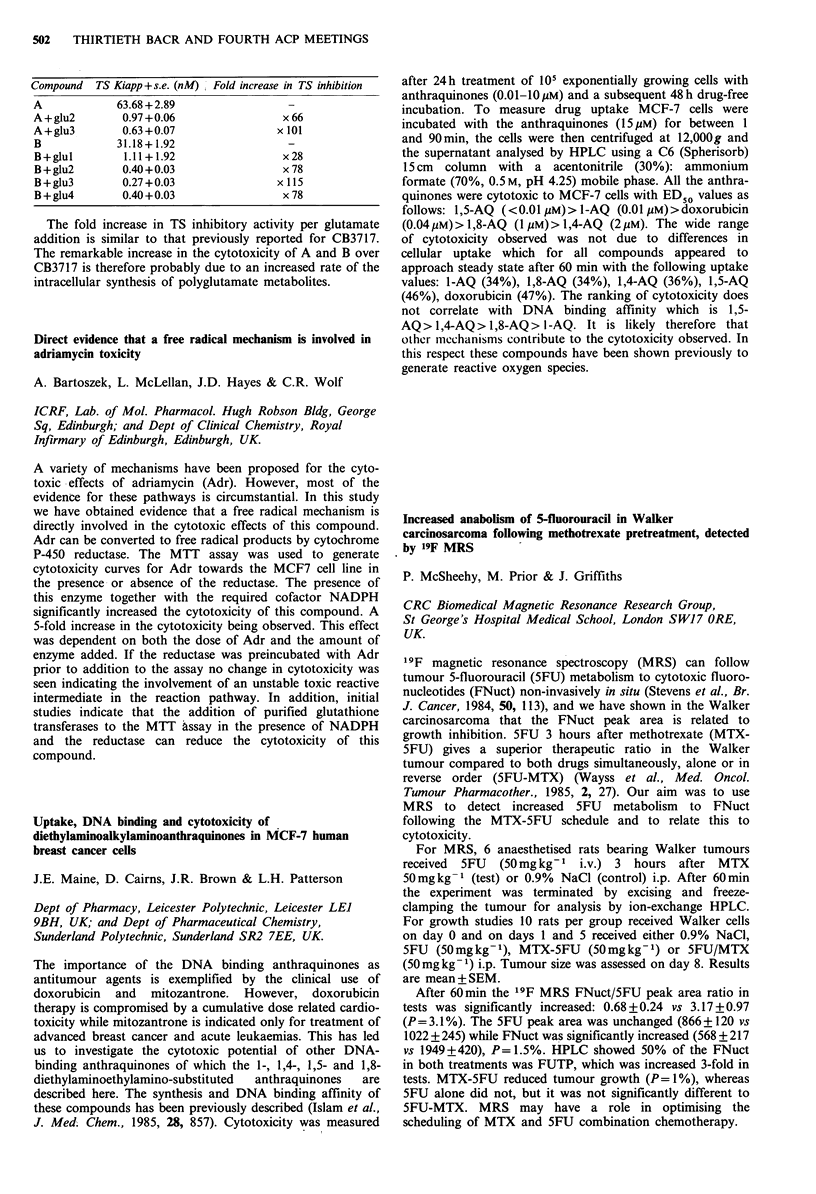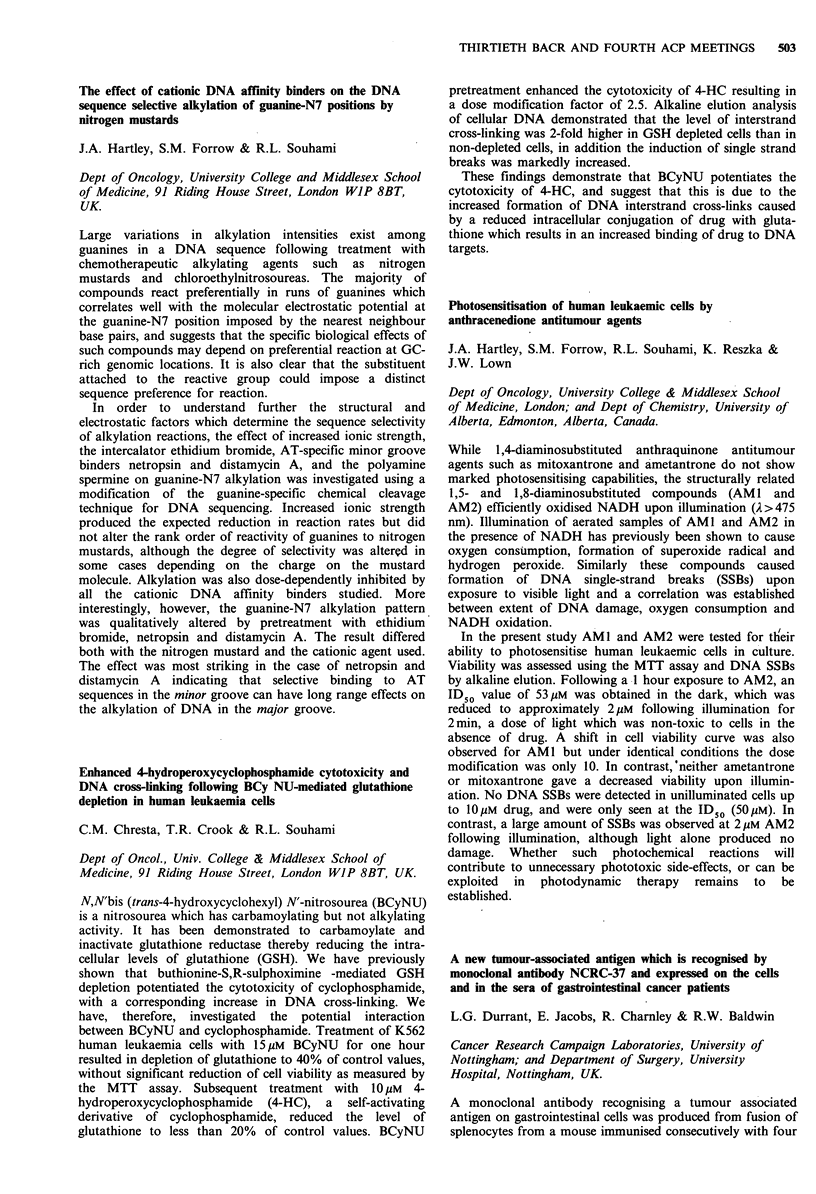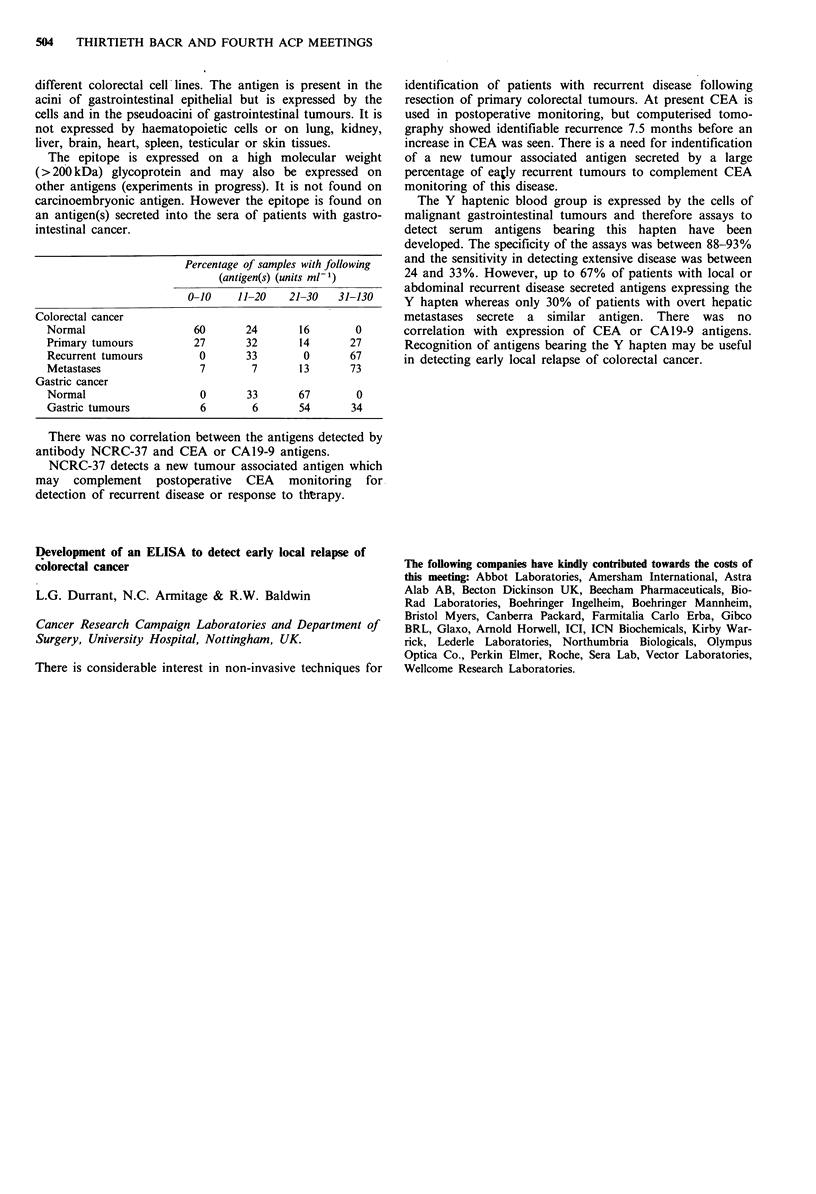# 30th Annual Meeting of the BACR & 4th Annual Meeting of ACP - 10-12 April 1989

**Published:** 1989-09

**Authors:** 


### BACR – posters

### The interaction of cellular factors with the NCR of HPV16

G.J. Sibbet & M.S. Campo

*Beatson Institute, Glasgow, UK.*

The progression of human papillomavirus 16 associated cervical intraepithelial neoplasia (CIN) to cancer is frequently accompanied by chromosomal integration of the viral genome. The cell line SiHa, derived from a cervical squamous cell carcinoma, contains a single integrated copy of HPV16.

We have mapped a complex pattern of nuclease hypersensitive sites (HSS), covering ~500 bp, at high resolution in SiHa chromatin to the tissue specific enhancer within the HPV16 non coding region (NCR). DNase footprinting (FP) of cloned DNA *in vitro* confirms the presence of cellular transacting factors (CTAFs) in SiHa nuclear extract with binding specificity for at least 13 sites in the HPV16 enhancer. The majority of FPs contain the consensus T/AGGCT/A, which is analogous to both the specific cytokeratin and ubiquitous CCAAT box motifs. However, the bound CTAFs found in nuclear extracts of SiHa as well as CaSki and HeLa are also found in non-epithelial K562 and MRC5 cells and appear to be related to the CCAAT box binding C/EBP but not to NFI/CTF, NFY or NFY*/CRF. One of the nuclear HSS in the HPV16 enhancer in SiHa contains overlapping DNA motif binding sites for AP1, C/EBP and glucocorticoid receptor (GR).

We are currently determining how these factors compete for their overlapping binding sites and how this effects the regulation of HPV16 in SiHa and cervical neoplasia.

This work was funded by the Cancer Research Campaign.

### The Harvey *ras* gene is activated in papillomavirus-associated carcinomas of the upper alimentary canal in cattle

M.S. Campo, I. Doherty, R. McCaffery, L.W. Coggins & I.M. Kennedy

*The Beatson Institute for Cancer Research, Garscube Estate, Switchback Road, Bearsden, Glasgow G61 1BD, UK; and The Institute of Virology, The University of Glasgow, Church Street, Glasgow G12, UK.*

The possible activation of *ras* sequences in papillomavirus-associated carcinomas of the upper alimentary canal of cattle was investigated by restriction enzyme and hybridisation analysis, and by DNA-mediated transformation of NIH 3T3 cells. The DNA from three cancers, two squamous cell carcinomas of the palate and the rumen, and one transitional cell carcinoma of the urinary bladder, showed anomalous restriction patterns for the c-Ha-*ras* sequences, indicating rearrangements and, in the case of the palate cancer, amplification. Cancer DNA was capable of inducing focus formation in the NIH 3T3 test. DNA from primary transformants was used in a second round of transformation and secondary transformants were analysed. Bovine Ha-*ras* sequences were detected in all transformants. In addition, high levels of ras transcripts were observed in several cancers. It is concluded that the Ha-*ras* gene is activated in alimentary canal carcinomas and the possible relationship between papillomavirus infection and activation of the ras gene is discussed.

This research was funded by the Cancer Research Campaign.

**The presence and expression of human papillomavirus DNA in oral squamous cell carcinoma**

W.A. Yeudall, D.G. MacDonald, R. Smith, D.S. Soutar & M.S. Campo

*Beatson Institute for Cancer Research, Glasgow Dental Hospital; and Canniesburn Hospital, Glasgow, UK.*

Nineteen tumour samples from patients with intraoral squamous cell carcinoma were screened for the presence of human papillomavirus (HPV) DNA by Southern blot hybridisation. Three of these (15.8%) were found to be positive, one each containing HPV-4, HPV-16 and HPV-18. Adjacent normal mucosa contained no or undetectable amounts of viral sequences. Normal buccal mucosa from 25 cancer-free individuals did not contain HPV DNA. In addition, all three viral genomes were expressed in the respective lesions, including the late genes, as detected by dot blot hybridisation. This is the first report of HPV-4 associated with an intraoral malignancy, and also the first report of expression of papillomavirus sequences in oral tumours.

This research was funded by the Cancer Research Campaign and the Medical Research Council.

**Co-operation of bovine papillomavirus type 4 and activated *ras* oncogene on the transformation of early passage bovine fibroblasts *in vitro***

R. Jaggar & M.S. Campo

*Beatson Institute, Glasgow, UK.*

Early passage bovine fibroblasts derived from fetal palate were transfected by electroporation with bovine papillomavirus type 4 (BPV4) molecularly cloned at 2 different restriction sites (BamH1 and Sst1:the BPV4 cloned at the BamH1 site interrupts the E1 ORF, while the Sst1 clone leaves the viral promoter and the entire early region intact), the activated human Ha-*ras* oncogene (pT24), an avian retroviral *myc* gene (pSVv*myc*), and combinations of these clones. After transfection, cells were grown to confluence and maintained for up to 12 weeks. Foci of densely growing cells appeared only in three cases – the combinations of: *ras*+*myc*, *ras*+BPV4 B1 and *ras*+BPV4 Sst1. No foci were obtained when cotransfecting BPV4 and the viral *myc* gene.

Foci were picked, generating cell lines which grew rapidly, piled up and showed higher confluent density than control monolayers. We are examining the tumorigenic characteristics of these lines. BPV4 thus displays similar transformation properties to various oncogenic human papillomaviruses.

This research was funded by the Cancer Research Campaign and the Medical Research Council.

**Initial characterisation of an *in vitro* inhibitor of haematopoietic stem cell proliferation**

G.J. Graham, E. Wright, M. Freshney, N.M. Wilkie & I.B. Pragnell

*Beatson Institute for Cancer Research, Glasgow G61 1BD and MRC Radiobiology, Didcot OX11 0RD, UK.*

Considerable experimental evidence suggests the existence of soluble, local activities regulating the proliferation of the primitive haematopoietic stem cell (CFU-S). Two such activities have been described: a stimulator, which moves the normally quiescent stem cells into the cell cycle, and an inhibitor which triggers the stem cells out of cycle. These activities are proteinaceous in nature and appear to be products of distinct macrophage sub-populations. Both activities appear to be specific for the stem cell and show no activity on the more mature progenitor population. It has been shown that crude inhibitory material has the ability to protect the haematopoietic stem cell from cytotoxic drug treatment *in vivo*. In view of the clinical potential for this material in cancer treatment, purification and characterisation of this regulator is of considerable importance.

To date, attempts at purifying such activities have been hampered by all the problems common to the isolation of biological molecules from *in vivo* sources. We have recently detected an inhibitor-like activity from the 20-fold concentrated conditioned medium from J774.2 cells (a mouse macrophage cell line). This inhibitor preparation is active in removing primitive haematopoietic stem cells (CFU-S) from the cell cycle and is also active in inhibiting the development of macroscopic CFU-A colonies which are believed to be an *in vitro* equivalent of the CFU-S. As for the *in vivo* inhibitor, the *in vitro* equivalent has no effect on progenitor cells and appears therefore to be stem cell specific.

The above data will be discussed in more depth and results relating to the initial biochemical characterisation of the inhibitory activity will be presented. This study was supported by the Cancer Research Campaign.

**t(14;18) Studies in follicular lymphoma(FL) with *Bcl*-2, $J_H$ and $D_H$ region oligonucleotides**

F.E. Cotter, A. Tuszynski, C. Price, T.A. Lister, A.M.Z. Rohatiner & B.D. Young

*ICRF Department of Medical Oncology, St Bartholomew's Hospital, London EC1A 7BE, UK.*

Lymph node DNA from seventeen patients with FL was studied by the polymerase chain reaction using synthesised oligonucleotides with sequences corresponding to the *Bcl*-2 gene on chromosome 18, the immunoglobulin heavy chain ($J_H$) gene and diversity ($D_H$) region on chromosome 14. Reactions using Taq polymerase were carried out. The *Bcl*-2-$J_H$ junction sequence was present in 7 cases by gel electrophoresis, blotting of the amplified fragment and probing with radioactive labelled *Bcl*-2 probe. Reactions to amplify the reciprocal junction region of the t(14;18) translocation were carried out with a D (+) region and a *Bcl*-2 (−) primer on the same samples. Reciprocal $D_H$-*Bcl*-2 junctions were detected in 6 samples, 4 of which had previously shown *Bcl*-2-$J_H$ junctions. Dilutions of the DNA were made and detection of amplification was possible to a dilution of $10^5$. DNA from complete remission bone marrows (BM) for 3 of the 9 positive patients were studied pre and post *in vitro* treatment with B1 antibody and complement for autologous bone marrow transplantation. One demonstrated positive amplification with $J_H$ region primers in the untreated BM but none in the post treatment BM with $J_H$ or $D_H$ region primers. In conclusion, detection of the t(14;18) translocation appears to be maximised using both sets of primers.

**Human c-*myc* oncogene protein in activated lymphocytes and cell lines**

D.G. Spiller, A.F. Chapman, D.M. Tidd & H.M. Warenius

*CRC Department of Radiation Oncology, University of Liverpool, Clatterbridge Hospital, Wirral L64 4JY, UK.*

It has been reported by a number of authors that c-*myc* protein levels rise in lymphocytes within 3 to 5 hours of treatment with phytohaemagglutinin (PHA), and that antisense *myc* oligonucleotides can block this rise. We have investigated this system in order to determine the feasibility of using this model for testing the antisense oligonucleotides synthesised within the Department of Radiation Oncology. Untreated and PHA treated lymphocytes were kept in culture for up to 72 hours and c-*myc* protein levels were determined by Western blotting. Samples of $2 \times 10^7$ lymphocytes were removed at various times after PHA stimulation, lysed, and total protein was assayed using the BCA micro method prior to electrophoresis and transfer to nitrocellulose membranes. Using a number of commercially available anti c-*myc* antibodies the expected bands of c-*myc* protein did not appear at early time points though intense c-*myc* protein bands appeared in the 72 hour PHA treated samples. Lysates of MOLT4, Raji, HMY, UC, Colo 320, and HT29/5 cell lines all presented clear c-*myc* protein bands. Colo 320 cell lysates produced detectable bands of c-*myc* protein even when one hundredth of the protein loaded for lymphocytes was used. These results suggest that either the reported early rise in c-*myc* protein levels in stimulated lymphocytes did not occur under the conditions of our experiments or, it was orders of magnitude lower than the rise we have observed at 72 hours, and hence undetectable.

This work was sponsored by the Cancer Research Campaign.

### C-*erb*B2 oncoprotein expression in malignant and non-malignant tissues

A. McCann, P.A. Dervan, W.J. Gullick & D.N. Carney

*Mater Misericordiae Hospital, Dublin, Ireland; and Hammersmith Hospital, London, UK.*

Using a polyclonal antiserum (21N) to the C-*erb*B2 oncoprotein, a total of 405 primary malignant human tumours and 94 non-malignant tissues were immunohistochemically assessed for over-expression of this putative transmembrane receptor protein. Of the 405 primary carcinomas investigated 17% (32/191) of the breast carcinomas, 4% (1/23) of the colorectal adenocarcinomas, 2% (1/48) bladder carcinomas and 1% (1/84) of the non-small cell lung neoplasms showed intense positivity for this oncoprotein predominantly located at the cell membrane. None of the small cell lung carcinomas ($n=23$), prostatic adenocarcinomas ($n=23$) or malignant melanoma samples ($n=10$) were positive for the oncoprotein. Of the 94 non-malignant conditions investigated none of the benign breast conditions ($n=45$), non-malignant bladder conditions ($n=15$), benign prostatic hyperplasia samples ($n=10$), normal colorectal biopsies ($n=11$) and non-involved lung resection margins ($n=13$) showed any positivity for the C-*erb*B2 oncoprotein. This study shows that over-expression of the C-*erb*B2 oncoprotein is common in breast cancer but relatively rare in the other malignancies investigated. In addition over-expression appears to be confined to the malignant phenotype.

### The characterisation of cell lines from mink lung following oncogene transfection

M.Z. Khan, R.I. Freshney, A.-M. McNicol, D. Spandidos & D.J. Kerr

*CRC Department of Medical Oncology, University of Glasgow, Royal Infirmary and Beatson Institute, Glasgow, UK.*

The mink lung cell line MV1LU (ATCC CCL64) has been transfected with c-*myc*, and normal and activated H-*ras* (see abstract Kerr *et al.*) and the isolated cell lines designated MV1LU (parental), MLMCGM1-1 (c-*myc*), MLH06N1-4 (H-*ras*), MLH06T1-4 (activated H-*ras*1). These lines have been characterised by morphology, immunocytochemistry, and *in vitro* and *in vivo* growth kinetics. There are minor morphological differences with the T1-4 line showing greatest signs of diminished contact inhibition, and focus formation. All the lines stain positively with cytokeratin antibody (AE3, ICN). Growth rate and saturation density is higher in T1-4 ($t_d$:14.2 h; s.d.:$1.2 \times 10^6$ cells cm$^{-2}$) and N1-4 ($t_d$:14.1 h; s.d.:$1.1 \times 10^6$ cells cm$^{-2}$) than in the parental line ($t_d$:19.5 h; s.d.:$5.7 \times 10^5$ cells cm$^{-2}$) and M1-1 ($t_d$:18.2 h; s.d.:$5.7 \times 10^5$ cells cm$^{-2}$) This distinction is reflected in their growth as xenografts where T1.1 has a high take rate (100%) the shortest latent period (7d) and initial doubling time (4d), the parental line has a low take rate (20%), longest latent period (60d) and doubling time (9d). M1-1 and N1-4 have intermediate values with a possibility that N1-4 shows more aggressive growth than M1-1; both grow much more vigourously than the parental line. Transfection with all the oncogenes has increased growth *in vitro* and *in vivo*. Although activated H-*ras*1 has the greatest effect, normal H-*ras* is still almost as active as H-*ras*1, and gives tumours whose histological appearance suggests greater similarity, in terms of local invasion, to H-*ras*1 than to either the c-*myc* transfected or the parent line.

### pSV3neo transfection and gamma radiation sensitivity of human bladder tumour cells

C.N. Parris, J.R.W. Masters, M.H.L. Green & C.F. Arlett

*Institute of Urology, University College London, St Paul's Hospital, London; and MRC Cell Mutation Unit, Sussex University, UK.*

Normal fibroblasts can be transformed by transfection with the plasmid pSV3neo (Mayne *et al.*, *Exp. Cell Res.*, 1986, **162,** 3630). Following transfection, the cells are less sensitive to the lethal effects of ionising radiation (Arlett *et al.*, *Int J. Radiat. Biol.*, in the press). This change in sensitivity may be due to the introduction of SV40 T-antigen, contained within pSV3neo. To extend these findings, we transfected pSV3neo into three bladder cancer cell lines RT112, T24 and HT1376. T-antigen expression in the transfected cells was confirmed by immunocytochemical staining. Gamma radiation sensitivities of the parental lines and transfected sublines were compared following exposure to a range of doses of gamma radiation. Do and D values were derived from a least squares linear regression plot of log cell survival against dose of irradiation in Gy (see table).

| *Cell line* | *Do (Gy)* | *D (Gy)* |
|---|---|---|
| RT112 | 2.25 | 3.9 |
| RT112pSV3neo | 1.9 | 3.9 |
| T24 | 2.6 | 3.6 |
| R24pSV3neo | 2.0 | 3.0 |
| HT1376 | 1.7 | 3.5 |
| HT1376pSV3neo | 1.85 | 3.5 |

Following transfection with pSV3neo, the gamma radiation sensitivities of the tumour cell lines were unaltered. These data indicate that in cells already transformed, transfection with SV40 T-antigen does not modify radiation sensitivity.

**DNA repair fidelity and recombination proficiency in B16 melanoma metastatic variants**

B.A. Usmani, G.V. Sherbet & J. Lunec

*Cancer Research Unit, Medical School, University of Newcastle upon Tyne, Newcastle upon Tyne NE2 4HH, UK.*

The progression of malignant tumours appears to be associated with a rapid generation of metastatic variants, which has been attributed to an increased level of genomic instability. Although elevated mutation frequencies have been measured in highly metastatic tumours, the mechanisms involved are not known. In order to assess the contribution of DNA repair and recombination defects we have compared the efficiency of these processes between low (F1) and high metastasis (BL6 and ML8) variants of the B16 murine melanoma.

No difference in the rate or extent of DNA strand break religation was detected using the hydroxylapatite technique. The fidelity of DNA strand break repair was assessed by transfection with the plasmid PMH16, which carries two selectable genes, *neo* and *gpt*. Enzymatic breaks were produced within the *gpt* coding region. Following transfection, cells were subjected to sequential selection for intact *neo* and *gpt* genes. High metastasis variants showed a greater fidelity of religation of the cut *gpt* gene.

Chromosomal recombination frequencies were measured by the method of Subramani and Rubnitz (*J. Mol. Cell Biol.*, **5**, 659) using a plasmid (pDR1) which contains an interrupted *neo* gene. In preliminary experiments, the frequency of successful recombination of a functional *neo* gene following chromosomal integration of the plasmid was found to be $\sim 1 \times 10^{-5}$ for the F1 (low metastasis) clones and $\sim 5 \times 10^{-7}$ for the highly metastatic BL6 cells.

Thus, although the more metastatic variants show a greater fidelity of DNA strand break religation, they appear to exhibit a reduced capacity for successful homologous recombination. Defective homologous recombination may contribute to the elevated mutation frequency reported for highly metastatic tumours.

**Protection of oncogene anti-sense oligodeoxynucleotides against degradation by serum exonuclease**

D.M. Tidd, A.F. Chapman, D.G. Spiller, C.C. Goodwin, J.A. Tidd & H.M. Warenius

*CRC Department of Radiation Oncology, University of Liverpool, Clatterbridge Hospital, Wirral L63 4JY, UK.*

An anti-sense oligodeoxynucleotide 15-mer complementary to the first 5 codons of the human c-*myc* gene has been reported to inhibit *myc* protein synthesis in and proliferation of PHA stimulated lymphocytes and HL60 promyelocytic leukaemia cells in culture (Wickstrom *et al.*, *Proc. Natl Acad. Sci. USA*, 1988, **85**, 1028). Cultured Colo 320 and HT29/5 human colon carcinoma cells were shown to synthesize high concentrations of *myc* proteins and yet their proliferation was unaffected by the same anti-sense oligodeoxynucleotide (50 μM). It was subsequently demonstrated that the general applicability of anti-sense oligodeoxynucleotides in studies of oncogene function in cultured tumour cells would be limited by their rapid degradation by nucleases present in the serum components of the tissue culture media. In order to determine the relative contributions of endonuclease and exonuclease activities in fetal calf serum to oligodeoxynucleotide destruction, we have tested chimeric molecules protected against exonuclease attack with terminal methylphosphonate diester linkages. These experiments indicated that 3'-phosphodiesterase is the predominant nuclease responsible for oligodeoxynucleotide degradation by fetal calf serum and that substantial increases in the lifetime of intact molecules in tissue culture medium may be achieved by blocking their 3'-termini against attack by this enzyme.

This work is supported by the Cancer Research Campaign.

**Identification of hypervariable regions containing multiple tandem repeat sequences by electron microscopy**

L.W. Coggins & M. O'Prey

*Beatson Institute for Cancer Research, Garscube Estate, Switchback Road, Bearsden, Glasgow G61 1BD, UK.*

Hypervariable regions (HVRs) in eukaryotic genomes consist of multiple tandem repeats of short (10–50 bp) sequences. The copy number differs between individuals, providing useful markers for 'genetic fingerprinting' and creating restriction fragment length polymorphisms (RFLPs) some of which show linkage with diseases including cancers.

A HVR, termed the variable tandem repeat (VTR) sequence, lies 3' to the human Harvey-*ras* oncogene. It consists of 29 copies of a 28 bp sequence in a 6.5 kb DNA fragment molecularly cloned in plasmid pT24. Supercoiled pT24 molecules cut with a restriction enzyme, and identical molecules subsequently denatured and reannealed to form homoduplex DNA, were examined with an electron microscope. Some molecules in both preparations contained partly hybridised pairs of small single-stranded DNA loops. The structures varied in morphology, but all mapped to the position of the VTR and could be derived by misaligned hybridisation of these sequences. In preparations of a plasmid carrying a major HVR (228 × 17 bp), 3' to the human alpha-globin gene complex, many homoduplex molecules contained larger, more morphologically variable and more complex looped structures. Electron microscopy thus provides a method of mapping multiple tandem repeat sequences with respect to restriction enzyme sites in cloned DNA.

**The design of DNA sequence recognition elements based on berenil**

T. Jenkins, C. Laughton & S. Neidle

*CRC Biomolecular Structure Unit, Institute of Cancer Research, Sutton, Surrey SM2 5NG, UK.*

The anti-trypanosomal agent berenil has been shown to possess DNA-sequence recognition ability. Footprinting studies have revealed preferential binding to clusters containing at least 4 A-T base pairs (Portugal & Waring, *Eur. J. Biochem.*, 1987, **167**, 281). We are interested in berenil as a 'building block' in the design of larger DNA-sequence recognition elements. X-ray crystallographic and molecular modelling studies have shown that berenil adopts a conformation isohelical with B-DNA, enabling efficient binding in the minor groove (Pearl *et al.*, *Nucl. Acid Res.*, 1987, **15**, 3469). Subsequent molecular modelling on more than 36 DNA-octamer/berenil complexes has revealed a preference for binding with a 3 base-pair stagger and an energetic preference for A-T over G-C sequences in accord with DNA footprinting results. These modelling studies have been extended to investigate the potential of berenil-based oligomers for the recognition of longer stretches of DNA. A dimer has been designed which will bind, with minimal distortion, to stretches of B-DNA seven base-pairs long.

Molecular mechanics calculations indicate that the dimer maintains the A-T sequence preference of berenil over the extra base-pairs.

**$^{32}$P-postlabelling analysis of the effect of butylated hydroxyanisole on the level of DNA adduction by aristolochic acid in the rat forestomach and liver**

M.N. Routledge & R.C. Garner

*Cancer Research Unit, University of York, Heslington, York YO1 5DD, UK.*

The non-genotoxic food anti-oxidant butylated hydroxyanisole (BHA) has been shown to cause cancer in the forestomach of the rat (Ito *et al., JNCI,* 1983, **70,** 343) but the mechanism by which this occurs is not clear. In this study the sensitive $^{32}$P-postlabelling assay (Randerath *et al., PNAS,* 1981, **78,** 6126) was used to confirm that BHA does not bind to DNA of the rat forestomach (target organ) or liver (non-target organ) when administered by oral intubation at a dose of $1\,\mathrm{g\,kg^{-1}}$ body weight daily for fourteen days.

The effects of co-dosage of BHA with aristolochic acid (AA), a potent genotoxic forestomach carcinogen that forms DNA adducts detectable by postlabelling (Schmeiser *et al., Carcinogenesis,* 1988, **9,** 297) on AA adduct formation, were investigated. Male Wistar rats were dosed orally with either BHA (1 g kg body weight$^{-1}$ day$^{-1}$ in corn oil) and AA (1 mg kg body weight$^{-1}$ day$^{-1}$ in water) or corn oil and AA for 5 days in groups of three. DNA was extracted from liver and forestomach and $^{32}$P-postlabelled using the nuclease P1 enhancement method (Reddy & Randerath, *Carcinogenesis,* 1986, **7,** 1543).

It was observed that levels of aristolochic acid DNA adduction, as determined by Cerenkov assay, were two or three times higher when BHA was co-administered, as compared to when it was not, in both liver and forestomach. This work confirms that BHA does not bind to DNA directly or indirectly *in vivo*. Increased levels of adduction by aristolochic acid in the presence of BHA could be due to the induction of microsomal enzymes by BHA, leading to increased production of activated metabolites of AA.

**Immunological detection of AFB$_1$-DNA and BSA adducts in animal and human tissues**

J. Harrison & R.C. Garner

*Cancer Research Unit, University of York, Heslington, York YO1 5DD, UK.*

Aflatoxin B$_1$ (AFB$_1$) is a potent hepatocarcinogen for a number of laboratory animal species and has been epidemiologically linked to human primary hepatocellular carcinoma (PHC) in areas of the world with high food contamination. The effect of chronic human exposure to low AFB$_1$ levels are unknown. New sources of exposure for the Western world have recently been identified in imported foods, such as dried figs.

We have developed immunological methods to concentrate and quantify both AFB$_1$-DNA and protein adducts using a mouse monoclonal antibody raised against AFB$_1$. Recovery of AFB$_1$-guanine or AFB$_1$-FAPy, the major *in vivo* DNA adduct, is greater than 90% using immunoaffinity chromatography, followed by ELISA, for quantification. Recovery of AFB$_1$-lysine, the major serum albumin adduct, was also greater than 90%.

Using the combination of immunoaffinity concentration and ELISA, we have identified AFB$_1$ adducts in rats dosed with AFB$_1$ and humans thought to be exposed to this mycotoxin. Our work demonstrates the feasibility of human dose monitoring to environmental carcinogens.

**The quantitation of benzo(a)pyrene DNA adducts detected in mouse skin and lung tissue comparing $^{32}$P-postlabelling and scintillation counting**

M.M. King & R.C. Garner

*Cancer Research Unit, University of York, Heslington, York YO1 5DD, UK.*

Cigarette smoking is a major cause of lung cancer; approximately 50 carcinogenic chemicals are known to be associated with cigarette smoke. Carcinogen DNA adduct formation is recognised as a key factor in the initiation of chemical carcinogenesis. Using highly-sensitive $^{32}$P-postlabelling (Reddy *et al., Carcinogenesis,* 1984, **5,** 231), smoking-related adducts have been demonstrated in the human placenta (Everson *et al., JNCI,* 1988, **80,** 567) and a linear relationship has been established between adduct levels in the human lung and daily and lifetime-cigarette consumption (Phillips *et al., Nature,* 1988, **336,** 790). However, when the same sets of samples were examined using a highly-sensitive immunoassay, there were inconsistencies when results from $^{32}$P-postlabelling and immunoassay were compared.

In order to investigate this problem further, controlled animal experiments have been set up. Groups of 4 mice were topically treated with a smoking-related chemical carcinogen, Benzo(a)pyrene. The dose range of carcinogen applied was between 0.1 $\mu$mol and 0.9 $\mu$mol BP, each sample containing 150 $\mu$Ci ($^3$H)BP. DNA from skin and lung tissue was analysed for adduct content, initially by $^{32}$P-postlabelling, using the nuclease P1 enhancement method (Reddy & Randerath, *Carcinogenesis,* 1986, **7,** 1543) and scintillation counting. $^{32}$P-postlabelling results showed greater adduct levels in skin DNA than lung DNA and a clear dose–response relationship was obtained in both tissues. The range of adducts detected in the lung DNA was between 1.4 adducts per $10^8$ nucleotides at the lowest dose and 1 adduct per $10^7$ nucleotides at the highest dose, as compared to 1.7 adducts per $10^6$ nucleotides and 4 adducts per $10^6$ nucleotides in skin DNA. Scintillation counts also showed greater adduct levels in skin DNA than lung DNA and a dose–response relationship in both tissues. The range of adducts in lung DNA was between 6.2 adducts per $10^7$ nucleotides and 4 adducts per $10^6$ nucleotides, as compared to 1 adduct per $10^6$ nucleotides and 1.6 adducts per $10^5$ nucleotides in skin DNA. The discrepancies indicated between these results may affect accurate quantitation of more complex human samples. The possible reasons for these differences are under investigation.

**Thymidine kinase gene expression in UV-irradiated human lymphoblastoid (Raji) cells**

V.J. McKelvey, L.A.J. Stefani & P.G. McKenna

*Biomedical Sciences Research Centre, University of Ulster, Coleraine BT52 1SA; and NI Centre for Genetic Engineering, Medical Biology Centre, Queen's University, Belfast BT9 7BL, UK.*

Deficiency of the salvage pathway enzyme thymidine kinase (TK) in Friend erythroleukaemia cells results in increased sensitivity to cell killing and mutagenesis following exposure to a variety of carcinogenic agents including UV light

(McKenna *et al., Cell Biol. Int. Res.*, 1981, **5**, 555; McKenna *et al., Genet. Res.*, 1982, **40**, 207). Work with other malignant cell lines indicates that TK deficiency only confers increased sensitivity in those cell lines which are normally proficient in DNA excision repair (McKenna *et al., Somat. Cell Mol. Genet.*, 1985, **11**, 239).

In this study messenger RNA (mRNA) TK levels were compared in untreated asynchronous Raji cells and in UV-treated asynchronous Raji cells at 2½ hours following UV-irradiation. Three doses of UV light were used, namely 2.5, 5 and $10\,J\,m^{-2}$. RNA was extracted from control and treated cultures and Northern blots were prepared. The TK gene probe was developed by subcloning the 1.4 Kb Bam HI fragment of the plasmid ptk 9, containing the cDNA clone of the human TK gene (Lin *et al., Proc. Natl Acad. Sci.*, 1983, **80**, 6528) into the riboprobe Gemini 1 transcription plasmid to obtain the recombinant TKG 9. *In vitro* transcription of Bgl 1 restricted TKG 9 produces a very efficient TK RNA hybridisation probe.

Autoradiographs were obtained following 24 hours exposure of TK hybridised Northern blots, and showed significantly elevated TK mRNA levels in Raji cells following $2.5\,J\,m^{-2}$ UV-irradiation relative to controls. The TK mRNA levels were observed to decrease towards control level with increasing dose of UV light, perhaps as a consequence of very severe cell damage resulting in cell death. The importance of TK in DNA repair of damage induced by low doses of light is indicated.

This Research was supported by the Ulster Cancer Foundataion.

### Effect of $O^6$-alkylguanines on eukaryotic DNA methylase

P.A. Hepburn & M.J. Tisdale

*CRC Experimental Chemotherapy Group, Pharmaceutical Sciences Institute, Aston University, Birmingham B47, UK.*

The effect of $O^6$-alkylguanines on eukaryotic DNA methylase has been investigated using enzyme from mouse L1210 ascites tumour cells partially purified through an Ultragel AcA34 column. While $N^7$-methylguanine 3-methyladenine, l-methyladenine and 5-methylcytosine had no effect on the activity of DNA methylase, the methylation of DNA *in vitro* was inhibited by $O^6$-methylguanine in a dose-related fashion. The inhibition could be counteracted by increasing the specific activity of the enzyme, and was not reversible by dialysis, suggesting that the $O^6$-methylguanine may be reacting with the enzyme protein in a manner analogous to that with the repair-enzyme, $O^6$-methylguanine methyltransferase ($O^6$MeGMT). Other $O^6$-alkoxyguanines were less effective in inhibiting the DNA methylase *in vitro* in the order ME > Et > Bu. Treatment of GM892 cells, which lack $O^6$MeGMT, with 0.1 mM $O^6$-methylguanine produced an initial decrease in DNA methylase activity within 1–2 h of treatment, followed by an increase in enzyme levels to approximately twice the starting value. This increase in enzyme activity was not inhibited by cycloheximide suggesting an increased processing of pre-formed enzyme. The inhibition of DNA methylase by $O^6$-methylguanine was not observed in Raji cells which are proficient in $O^6$MeGMT. These results suggest that DNA methylase may react with $O^6$-methylguanine in cells lacking $O^6$MeGMT, an effect which would have important consequences in terms of carcinogenesis and gene expression.

### Transcutaneous oxygen tension – a guide to effective perfusion in regional chemotherapy for malignant melanoma

D.S. Byrne, R. Blackie, J. Hughes, G. Burnside, R.M. MacKie & A.J. McKay

*Department of Surgery, Gartnavel General Hospital, Glasgow; Department of Medical Oncology, Glasgow University; and Department of Dermatology, Western Infirmary, Glasgow, UK.*

Isolated limb perfusion (ILP) is an established mode of regional cancer therapy. Its principal application is in the treatment of malignant melanoma of the extremities using melphalan. The surgical technique is very similar in most centres; however, the criteria by which an adequate blood flow is achieved in the isolated limb are complex and variable.

We have measured transcutaneous oxygen tension ($P_tcO_2$), blood flow and intra-arterial pressure in 20 patients undergoing ILP.

We observed a positive correlation between $P_tcO_2$, blood flow and intra-arterial pressure. We also found a positive correlation between $P_tcO_2$ and tissue melphalan levels measured by HPLC.

We conclude that $P_tcO_2$ monitoring offers a simple and useful measurement of the adequacy of tissue perfusion during ILP. Using this measurement it should be possible to ensure maximal tissue delivery of melphalan.

### Relationship between reductive drug metabolism of anthracyclines in microspherical form and anti-tumour activity

N. Willmott, J. Cummings, E. Marley & J. Smyth

*Department of Pharmacy, University of Strathclyde, Glasgow; and ICRF, Department of Medical Oncology, Western General Hospital, Edinburgh, UK.*

Protein microspheres were developed as a carrier to (a) target incorporated cancer therapeutic agents to solid tumour deposits, (b) increase duration of exposure to incorporated agent. To investigate the latter point with respect to anthracyclines a model was developed involving direct intratumoral injection of a SC growing rat carcinoma (Sp107) with drug in solution or in microspherical form. We have reported increased activity of doxorubicin in microspherical form compared to drug in solution. This was associated with retention of drug in tumour tissue and reductive metabolism to 7-deoxyaglycones following administration of $60\,\mu g$ doxorubicin as measured by HPLC with fluorescence detection (mean peak metabolite concentration (48 h): microspheres $3.7\,\mu g\,g^{-1}$; solution $<0.05\,\mu g\,g^{-1}$). In contrast to doxorubicin, 4′-deoxydoxorubicin in microspherical form was no more active than drug in solution and less active than doxorubicin in microspherical form. Thus, if the reductive metabolism observed were an important determinant of activity of doxorubicin in microspherical form it would be predicted that 4′-deoxydoxorubicin in this form would not be metabolised to 7-deoxyaglycones in tumour tissue. The metabolic profile of 4′-deoxydoxorubicin in tumour tissue was in fact similar to doxorubicin in that following intratumoral injection of $60\,\mu g$ of parent drug in microspherical form reductive metabolism to 7-deoxyaglycones occurred (mean peak metabolite concentration (24 h): microspheres $2.7\,\mu g\,g^{-1}$; solution $0.3\,\mu g\,g^{-1}$). Thus, as regards doxorubicin, reductive metabolism of drug in tumour tissue when injected in microspherical form does not appear to be an important component of its increased activity; however, these results have implications for the type of chemotherapeutic agent that could benefit from incorporation into microspheres.

**The influence of drug exposure parameters on the predictive value of the clonogenic assay in an experimental tumour model**

R.M. Phillips, M.C. Bibby & J.A. Double

*Clinical Oncology Unit, University of Bradford, West Yorkshire BD7 1DP, UK.*

The clonogenic assay has been shown to be a poor predictor of response in numerous clinical trials. Several problems may contribute to the poor prediction of tumour sensitivity, one of which is the inadequate representation *in vitro* of drug exposures achieved *in vivo*. The aim of this study was to assess whether or not standard methods of 'imitating' *in vivo* drug exposures (i.e. 1 hour exposure at achievable drug C x t parameters – protocol A) provide a better or worse prediction of tumour response than an assay where drug exposures *in vitro* and *in vivo* are identical (protocol B). In protocol B, tumour cells, derived from an ascitic murine adenocarcinoma of the colon, MAC 15A tumour were first treated with cytotoxic drugs *in vitro* (at achievable plasma drug C x t exposures for 1 hour) before being either inoculated into mice to assess anti-tumour effects *in vivo* or cultured *in vitro* to determine clonogenic cell kill. Adriamycin (ADR), 5-fluorouracil (5-FU), methotrexate (MTX), ThioTEPA (TSPA) flavone acetic acid (FAA) and TCNU were used. In protocol A, all drugs were administered intraperitoneally at their respective maximum tolerated doses, and anti-tumour effects were assessed on the basis of differences in the median survival time of treated and control groups. The predictive value of both protocols A and B were good for ADR and TCNU (true positive predictions) and for MTX and FAA (true negative predictions). For 5-FU and TSPA, a poor correlation (false positive prediction) exists using both protocols A and B suggesting that other factors apart from achievable drug exposures have a significant influence on the final outcome of chemotherapy *in vivo*. For ADR, TCNU, MTX and FAA, the use of standard drug C x t principles in the analysis of the clonogenic assay does correlate well with tumour response in the MAC 15A tumour model.

**Acute dosing with pyridoglutethimide and aminoglutethimide inhibits hepatic cytochrome *P*-450**

Z. Damanhouri, P.J. Nicholls & H.J. Smith

*Welsh School of Pharmacy, UWCC, PO Box 13, Cardiff CF1 3XF, UK.*

Pyridoglutethimide (PD), a new aromatase inhibitor (AI) with greater selectivity of action on steroidogenesis than aminoglutethimide (AG), is currently on clinical trial in women with breast cancer. Such patients often receive several drugs concurrently and it is important to assess the potential of any new agent for drug interactions, a common site being hepatic cytochrome *P*-450. This work compares the ability of equimolar oral doses of PD (56 mg kg$^{-1}$) and AG (60 mg kg$^{-1}$), when administered acutely (1 h beforehand), to inhibit the *P*-450-dependent metabolism of various compounds. In female mice, pentobarbitone (P, 45 mg kg$^{-1}$ i.p.) hypnosis, zoxazolamine (Z, 120 mg kg$^{-1}$ i.p.) paralysis and $^{14}CO_2$ exhalation $t_{0.5}$ after $^{14}$C-antipyrine (A, 0.3 $\mu$Ci i.p.) were significantly ($P<0.05$) prolonged by PD and AG (with P from 28 ± 4 to 202 ± 28 (PD) and 172 ± 26 (AG) min; with Z from 52 ± 4 to 94 ± 10 (PD) and 81 ± 7 (AG) min; with A from 20 ± 1 to 43 ± 1 (PD) and 31 ± 2 (AG) min). Pretreatment of female rats with PD and AG elevated 1 h plasma levels of phenytoin (15 mg kg$^{-1}$ i.p.) from 2.7 ± 0.5 to 7.3 ± 0.8 (PD) and 4.5 ± 0.3 (AG) $\mu$g ml$^{-1}$. A similar effect occurred with the 4 h plasma levels of dicoumarol (8 mg kg$^{-1}$ p.o.) from 26 ± 2 to 35 ± 1 (PD) and 35 ± 2 (AG). The hypoglycaemic action of tolbutamide in rats was potentiated by both PD and AG. The oxidation of 4-nitro-anisole and dicoumarol by rat liver microsomes was non-competitively inhibited by PD and AG. The results demonstrate that both drugs inhibit hepatic *P*-450. This effect should be recognised as a potential source of drug interactions when these AIs are initially given to patients already receiving a variety of medication.

**Kinetic basis for paediatric dosage of vincristine**

B.J. McDermott, M. Kohli, R.F. Stevens, A.J.P. Pearson & P. Moris-Jones

*Department of Therapeutics and Pharmacology, The Queen's University of Belfast; Royal Manchester Children's Hospital; and The Medical School, University of Newcastle upon Tyne, UK.*

Because of immaturity in hepatic conjugation, the kinetics of vincristine (VCR) in infants will vary significantly compared with older children and adults, so that especially young children may not tolerate standard doses calculated on the basis of body surface area (BSA). An infant's BSA is relatively large in proportion to body weight (BW). Furthermore, about 25% of children with malignancy are malnourished at presentation and although the BSA does not change significantly, lean body mass may be altered considerably. In this study we compared the disposition of VCR given by the standard schedule of 1.5 mg m$^{-2}$ with that recommended for children with BSA less than 1 m$^{-2}$ of 0.05 mg kg$^{-1}$ (Woods *et al.*, *J. Pediatr.*, 1981, **98**, 642). The investigation was of randomised cross-over design in 6 patients (5M, 1F) of median age 38 months (14–75), BW 13.9 kg (9.1–26.5 kg) and BSA 0.59 m$^2$ (0.40–0.90 m$^2$). On the basis of BW, median dose given was 0.75 mg (0.46–1.25 mg) whereas it was 0.88 mg (0.60–1.35 mg) when calculated according to BSA and the associated increase was 27% (8–56%). Correspondingly, the median AUC$^{\infty}$ was greater after doses based on BSA (2.39 $\mu$g ml$^{-1}$ min$^{-1}$) than on BW (1.86 $\mu$g ml$^{-1}$ min$^{-1}$). When AUC$^{\infty}$ values associated with each schedule were correlated with age, they decreased with increasing age and the slope of the regression line describing the relationship with BSA was greater than that with BW, by a factor of 3, such that in the youngest child, the AUC$^{\infty}$ increased by 72%. Dosage calculated according to BSA in infants, therefore, results in proportionately greater exposure to the drug, which may account for the increased incidence of VCR-related neurological toxicity, compared to older children.

***N*-(2-hydroxypropyl)methacrylamide copolymer–antibody conjugates have greatly reduced immunogenicity**

P.A. Flanagan, B. Rihova, V. Subr, K. Ulbrich & R. Duncan

*CRC Laboratory, University of Keele, Staffordshire ST5 5BG; Institute of Microbiology and Institute of Macromolecular Chemistry, Czechoslovak Academy of Sciences, Prague, Czechoslovakia.*

*N*-(2-hydroxypropyl)methacrylamide (HPMA) copolymers have been developed as drug-carriers (Kopecek & Duncan,

*J. Controlled Rel.*, 1987, **6**, 315). Copolymer conjugates can be synthesised to incorporate pendant, oligopeptide side-chains terminating in either bound drug, or a suitable targeting residue, such as an antibody molecule. Immunogenicity is already known to be a major problem associated with the administration of antibodies to patients.

Two strains of mouse (A/J high responder; B10, low responder; Rihova *et al., Thymus,* 1988, **8**, 161) were immunised with HPMA copolymer (100 μg), IgG or IgG-HPMA copolymer conjugate (100 μg protein). The first immunisation used complete Freund's adjuvant subcutaneously, followed by two further immunisations intraperitoneally using alum precipitate. Mice were exsanguinated on the seventh day after the final immunisation and the serum separated by centrifugation (1000 *g*, 10 min, 4°C). The antibody titres (IgG and IgM) raised against the above antigens were determined using the ELISA technique. There is up to a 250-fold reduction in the antibody titer (IgG) raised in mice against human immunoglobulin fraction G (IgG) after conjugation to HPMA copolymer. Cross-reactivity of antisera revealed that polymer is externally disposed in the conjugate and this 'masking' of the protein antigen may account for the decrease seen. Experiments in rats have showed up to a 220-fold reduction in antibody titre (IgG) raised against murine monoclonal antibodies B72.3 and A5B7 after conjugation to HPMA copolymer. The activity of such conjugates incorporating drug will shortly be investigated.

### Development of a monoclonal antibody-based competitive ELISA for measuring mitozantrone levels in serum

S.U. Flavell & D.J. Flavell

*Monoclonal Antibody Unit, University Department of Pathology, Southampton General Hospital, Southampton SO9 4XY, UK.*

A monoclonal antibody (NO-1) was produced against the small anti-cancer drug Mitozantrone ($M_r$ 517.4) (Lederle Laboratories, Gosport, Hants, UK) following the immunization of a BALB/c mouse with Mitozantrone-Keyhole Limpet Haemocyanin conjugate. Employing this monoclonal antibody in a competitive ELISA with Mitozantrone-bovine serum albumin (MZ-BSA) as solid phase antigen, it was possible to accurately and reproducibly detect levels of Mitozantrone in pooled human serum in the range from 0.5 to 40 ng ml$^{-1}$. The assay displayed good reproducibility with intra-assay coefficients of variation of between 1.4 and 7.02% being obtained for sextuplicate samples at concentrations of Mitozantrone in serum ranging from 0.1 to 80 ng ml$^{-1}$.

The demonstration that it is possible to raise a specific monoclonal antibody against a very small molecule and the subsequent successful development of an ELISA for this molecule indicates that similar monoclonal-based immunoassays may be possible for other small molecules where quantitation at low levels by standard techniques poses analytical difficulties. Another possible role for a cytotoxic drug antibody of this nature may be in drug targetting where it may be possible to construct a bispecific antibody with anti-tumour cell specificity in one FAB arm and anti-drug specificity in the other. Thus, drug could be targetted to the tumour cell surface by antibody without the need for covalently coupling the drug to the antibody.

### Confirmation of a prognostic index for metastatic breast cancer

J.F.R. Robertson, R.I. Nicholson, I.O. Ellis, C.W. Elston & R.W. Blamey

*Nottingham City Hospital; and Tenovus Institute, Cardiff, UK.*

We have previously reported an index of prognosis from the time of diagnosis of metastatic breast cancer based on 4 factors: grade and oestrogen receptor (ER) of the primary tumour, site of initital metastases, disease free interval (DFI).

Retrospectively 3 groups were identified with index scores of <8, 8–16.5 and >16.5. Survival between the 3 groups was significantly different ($\chi^2 = 146.6$; 2 d.f., $P < 0.001$).

We have applied the prognostic index *prospectively* to a second group of 81 patients who have presented subsequent to calculating the index. The patient population and survival at 18 months for both the original group of patients and the prospective group are shown in the table.

| | *Prognostic index group* | | |
|---|---|---|---|
| *Patient group* | *A* | *B* | *C* |
| *Original* | | | |
| no. of patients (%) | 70 (37) | 79 (41) | 42 (22) |
| % survival at 18 months | 67 | 37 | 0 |
| *Prospective* | | | |
| no. of patients (%) | 26 (32) | 26 (32) | 29 (36) |
| % survival at 18 months | 100 | 33 | 14 |

Survival in the prospective group of patients was significantly different between the prognostic groups ($\chi^2 = 81.18$; 2 d.f.; $P < 0.001$). This index allows accurate prediction of the prognosis in systemic disease and should therefore be used for allocating patients to different treatment regimens.

### An evaluation of mucin-like carcinoma associated antigen (MCA) in breast cancer

E.H. Cooper, M.A. Forbes & A.K. Hancock

*Unit for Cancer Research, University of Leeds, Leeds LS2 9NL; and Bradford Royal Infirmary, Bradford BD9 6RJ, UK.*

Serum levels of mucin-like carcinoma associated antigen (MCA) were measured in 80 healthy women, 109 patients with breast cancer at presentation and in samples taken from 60 patients with active metastatic breast cancer. MCA levels in controls were <1.0–19.6 U ml$^{-1}$, median 6.2 U ml$^{-1}$: the levels in patients with stages I–III at presentation were not significantly different to controls. Longitudinal studies over 5–9 years in 20 patients with stage I and II disease who had remained tumour free showed a narrow MCA range for each individual patient, but the mean and range of a single measurement in a further 63 of these patients were similar to those of the normal controls. Rising MCA levels occurred in 12/14 patients who developed metastases in 2–8 years after surgery. 80% of patients with active metastatic disease had MCA levels >15 U ml$^{-1}$. MCA levels fell during clinical responses to therapy in metastatic cancer. In the context of follow up serum MCA levels appear to be a sensitive indicator of metastatic disease; caution is required in the interpretation of isolated measurements.

### Serum thymidine kinase – a tumour marker in advanced breast cancer

J.F.R. Robertson, K. O'Neill, G. McKenna & R.W. Blamey

*City Hospital, Nottingham; and University of Ulster, Coleraine, UK.*

The enzyme thymidine kinase (TK) is associated with DNA synthesis. TK serum levels were studied in normal controls, primary breast cancer (PBC) and advanced breast cancer (ABC). All values are given as pmol $ml^{-1} h^{-1}$.

| *Group* | *No. of patients* | *Mean value* | *s.d.* |
|---|---|---|---|
| Normal | 20 | 4.22 | ±1.08 |
| PBC | 60 | 6.22 | ±1.24 |
| ABC | 20 | 9.79 | ±7.56 |
| Analysis of variance $P<0.005$ | | | |

Twenty PBC patients were followed up after mastectomy by serial 3 monthly TK levels in the disease free interval. Four patients have developed systemic recurrence with a mean serum value of 14.3.

Ten ABC patients had serial TK levels 2 monthly during the first 6 months of primary hormone therapy. External assessment of response to treatment by UICC criteria has been obtained. The serum values fell in all 5 responders (mean 9.12–4.78) and rose in all 5 progressors (mean 8.62–38.56).

Serum TK reflects stage of disease being significantly elevated in advanced breast cancer both on serial estimations and in comparison with controls. Serial TK levels in ABC patients measures response to systemic therapy. TK appears to be a useful biochemical marker in breast cancer.

### Can growth fraction as estimated by Ki67 staining give an early prediction of the response of tumours to therapy?

L.G. Durrant, S.A. Watson, N.C. Armitage, R. Charnley & D. Morris

*Cancer Research Campaign Laboratories, University of Nottingham; and Department of Surgery, University Hospital, Nottingham, UK.*

Ki67 is a monoclonal antibody which recognises an antigen in all cycling cells. Freshly disaggregated colorectal tumour cells were stained by indirect immunofluorescence with Ki67 monoclonal antibody and analysed on the flow cytometer. The assay accurately estimated growth fractions of 5% and above.

Fifty colorectal tumours have been analysed and 10% of them had a growth fraction in excess of 30% and 29% a growth fraction between 10 and 30%. A further 28% had growth fractions of between 10 and 5% and the remaining 33% had growth fractions below 5%. The accuracy of the assays was good as the standard error of duplicate sample was 7.3±3.3%. However, if the tumour was subdivided into small biopsies prior to separate disaggregations a large variation in growth fraction was observed (57±20%).

This did not correlate with specific areas i.e. central necrotic area low proliferation and the extreme edges a high proliferation index. There was also variation between the growth fraction of biopsies taken at sigmoidoscopy and the growth fraction of the resected tumour. It was hoped to use this latter technique to assess treatment administered post sigmoidoscopy and prior to tumour resection. However, due to the sampling variation it is unlikely to be informative.

In highly proliferating tumours, with a presumably bad prognosis, therapy may reduce the growth rate significantly compared to placebo treated patients and this would be rapidly detected by this assay.

### Ki67 in primary breast cancer

J.F.R. Robertson, K. Walker, R.I. Nicholson, A.P. Locker, I.O. Ellis & R.W. Blamey

*Nottingham City Hospital; and Tenovus Institute, Cardiff, UK.*

Ki67 is a proliferation antigen associated with actively cycling cells. One hundred and four patients with primary operable breast cancer had Ki67 measured on tumour sections by immunocytochemistry. Forty-five patients were positive for Ki67 binding (>5% tumour nuclei stained). There was an excellent correlation with grade and mitotic index.

| | *No. of tumours positive for Ki67 (%)* | | *No. of tumours positive of Ki67 (%)* |
|---|---|---|---|
| Grade I ($n=23$) | 6 (26%) | Mitotic index 1 ($n=42$) | 9 (21%) |
| Grade II ($n=33$) | 12 (38%) | Mitotic index 2 ($n=16$) | 12 (75%) |
| Grade III ($n=48$) | 33 (69%) | Mitotic index 3 ($n=46$) | 30 (65%) |
| $\chi^2=30.7$; 2 d.f.; $P<0.001$ | | $\chi^2=21.95$; 2 d.f.; $P<0.001$. | |

There was no correlation between Ki67 and tumour size nor lymph node stage. Patients with Ki67 tumours had an increased number of recurrences.

Ki67 measured by immunocytochemistry may provide the basis of a semi-automated method for histological grading.

### Measurement of placental alkaline phosphatase in benign and malignant pleural effusions

R.J. Fergusson, L.W. Duncan, F.G. Hay, J. Fisken, M.A. McIntyre, J.E. Roulston & R.C.F. Leonard

*Departments of Clinical Oncology and Pathology, Western General Hospital and Department of Clinical Chemistry, Royal Infirmary, Edinburgh, UK.*

Placental alkaline phosphatase (PLAP) has been shown to be produced by a variety of human tumours. It has been extensively studied in ovarian cancer where it may have some use as a tumour marker. As it has been found in association with lung cancer we have assessed its potential as a diagnostic marker of malignancy in pleural fluid. Sixty patients with 'benign' and malignant pleural effusions were studied: 12 had a primary lung tumour, 23 had evidence of secondary tumour (7 ovary, 7 breast, 3 adenocarcinoma, 3 lymphoma and 3 others) and 25 had a non malignant aetiology for their effusion (10 cardiac failure, 5 related to infection, 2 rheumatoid, 1 TB, 1 thromboembolism disease and 6 unknown). Patients in this last group were followed for up to one year to exclude an occult malignancy. PLAP was measured using the two monoclonal antibodies H17E2 and H317 by an ELISA method. Mean pleural PLAP levels (±s.d.) were similar in the groups with primary lung cancer (0.48 IU $l^{-1}$ ±0.47) and with 'benign' effusions (0.54 m $l^{-1}$ ±0.86). PLAP levels in patients with secondary tumours appeared higher (0.88 IU $l^{-1}$ ±2.02) but this may be explained by the higher levels associated with ovarian primaries (2.16 IU $l^{-1}$ ±3.5). Smoking habits did not influence PLAP levels. We conclude that the measurement of PLAP in pleural fluid is unhelpful as a diagnostic aid apart from perhaps identifying a small subgroup of patients with metastatic ovarian tumours.

### Serum CA19-9, CA15-3, CA72-4 and TATI in epithelial ovarian cancer (EOC) role in relation to CA125

J. Fisken, A. Bowman, R.C.F. Leonard, J.E. Roulston

*University Department of Clinical Chemistry and Clinical Oncology, Royal Infirmary, Edinburgh EH3 9YW, UK.*

Serial measurement of tumour markers (TMs) is a feasible means of determining response to chemotherapy and detecting preclinical recurrence of EOC, without recourse to second-look laparotomy, where appropriate markers exist. Serum CA125 now has well established but limited clinical utility, not all tumours secrete it, thus highly specific '2nd-line' TMs to CA125 are required. We investigated the above 4 novel TMs in this respect, all reportedly elevated to varying degrees in EOC, by assaying serial serum samples from 42 patients with EOC. The number of samples with elevated levels are shown below.

| | *No. of patients* | *CA125 >35 U ml⁻¹* | *CA153 >30 U ml⁻¹* | *CA199 >33 U ml⁻¹* |
|---|---|---|---|---|
| Stage | | | | |
| I+II | 19 | 27/82 | 15/96 | 18/96 |
| III+IV | 23 | 51/86 | 40/109 | 37/109 |
| Histology | | | | |
| Serous | 17 | 41/73 | 30/93 | 16/93 |
| Endometrioid | 14 | 26/55 | 20/63 | 17/63 |
| Mucinous | 8 | 8/34 | 4/40 | 10/40 |

| | *No. of patients* | *CA72–4 >10 U ml⁻¹* | *TATI >20 μl⁻¹* | *>1 Marker elevated* |
|---|---|---|---|---|
| Stage | | | | |
| I+II | 19 | 8/34 | 9/66 | 41/96 |
| III+IV | 23 | 10/41 | 36/61 | 85/109 |
| Histology | | | | |
| Serous | 17 | 3/30 | 24/55 | 64/93 |
| Endometrioid | 14 | 8/27 | 13/35 | 43/63 |
| Mucinous | 8 | 6/15 | 3/31 | 15/40 |

With a slight degree of overlap, TATI, CA19-9, CA15-3 and CA72-4 were elevated in 20, 8, 4 and 3 cases respectively where CA125 was negative, and a further 4, 16, 10 and 1 cases respectively awaiting CA125 assay. A previous study found TATI to be a more specific marker of endometrioid EOC than CA125 (Fisken *et al.*, *Br. J. Cancer*, Abstract in press). TATI and possibly CA19-9 look promising '2nd-line' markers of endometrioid and mucinous EOC respectively, however further clinical details are required to assess the role, if any, of these markers in relation to CA125.

### A prospective assessment of the tumour markers CA50, CA195 and CEA in prediction of tumour stage and recurrence in colorectal cancer

P.M. Sagar, E.H. Cooper & P.J. Finan

*Department of Surgery and Unit for Cancer Research, Leeds General Infirmary, Leeds LS1 3EX, UK.*

CA50 and CA195, two newly developed carcinoma-associated carbohydrate antigens, may be clinically useful markers in monitoring patients following curative colorectal cancer surgery. We report a study comparing CA50 and CA195 with CEA in the prediction of stage and recurrence in 102 consecutive patients. Pre- and serial postoperative serum samples were obtained. CA50 was assayed using a time resolved fluoro-immunometric method and CEA and CA195 by radioimmunoassay. Patients were reviewed 3 monthly with clinical examination, liver function tests, faecal occult bloods, 6 monthly liver ultrasound and annual pelvic CT and colonoscopy. Median follow-up was 16 months (range 3–25). Preoperative serum levels of all three markers increased with Dukes' stage and discriminated localised (Dukes' A, B) from advanced disease (CEA and CA50 $P<0.01$, CA195 $P<0.002$, Mann–Whitney U test). The markers failed to differentiate Dukes' A from B and only CA50 differentiated Dukes' C from D ($P<0.05$). Out of 80 patients who underwent a potentially curative resection, 23 have shown a subsequent elevation in one or more tumour markers, but only 8 have developed proven recurrence-marker elevation preceding clinical detection in 4 (median lag period 3 months). Two patients have developed recurrence without marker elevation. CA50 and CA195 proved no better than CEA in predicting recurrence. The value of these tumour markers remains to be determined.

### Tumour biochemistry and response to treatment studied by clinical $^{31}P$ magnetic resonance (MR) spectroscopy

C.J. Twelves, M. Lowry, D. Porter, M. Smith, P. Heasley, P. Harper, R.D. Rubens & M.A. Richards

*Clinical Oncology Unit and Division of Radiological Sciences, Guy's Hospital, London SE1 9RT, UK.*

Animal tumours have characteristic biochemical features which change in response to chemotherapy (CT) or radiotherapy (RT) before alterations in tumour size are detectable. Human metabolism can now be studied non-invasively with MR spectroscopy. We have obtained 20 MR spectra from 7 patients (pts) with large superficial tumours (4 breast cancer, 3 lymphoma). Serial spectra were obtained in 4 pts after CT or RT.

Examinations were made on a 1.5 Tesla Philips Gyroscan imaging and spectroscopy system with a purpose built 5 cm diameter surface coil placed over the tumour. Tumour size was measured and coil position confirmed with MR images. $^{31}P$ spectra were acquired and processed using a standard technique. Peak heights of phosphomonoesters (PME), inorganic phosphate (Pi), phosphodiesters (PDE), phosphocreatine (PCr), $\gamma$-, $\alpha$- and $\beta$-ATP were measured and ratios for PDE/PME and $\beta$-ATP/Pi calculated.

Breast cancers studied before treatment were characterised by high PME, PDE and Pi and by low $\beta$-ATP/Pi. Lymphoma spectra differed in that more PCr was detected. Spectra in 1 breast cancer treated with RT and 1 lymphoma treated with CT showed marked changes within 24 hrs of starting treatment, before tumour size changed. Both pts subsequently responded clinically. In contrast, spectra from 2 other pts (1 breast cancer, 1 lymphoma) did not appear to change with treatment, and neither patient responded clinically.

Tumour metabolism can be studied by MR spectroscopy and changes in tumour biochemistry after treatment detected. These biochemical changes reflect alterations in membrane turnover (PME+PDE/PME) and cellular energy status ($\beta$-ATP/Pi) within tumours.

**The pedometer: an objective assessment of quality of life?**

J.H. Davies, P.R. Malone, S.J. Harland & R.J. Shearer

*Department of Urology, St Georges Hospital and St Peters Group of Hospitals and Institute of Urology, London, UK.*

Subjective assessment of quality of life of cancer patients is an important part of clinical trials and is usually based on a scoring system. Those in use (Karnofsky and Eastern Co-operative group scale; ECOG) are, however, time consuming and open to criticism of interpretation. Objective assessment of patients response includes the measurement of haematological, biochemical and radiological indices. We have evaluated the use of an electronic pedometer as an objective measurement of patients mobility before and during treatment for advanced urological cancers. Eight patients with advanced prostatic cancer were treated with an aromatase inhibitor. One patient with disseminated testicular cancer was treated with combination chemotherapy. Daily totals were recorded on a Scotcade 'Pe-Do' pedometer before and during treatment. Subjective assessment was by the ECOG scale. Standard objective parameters were measured regularly. In the patient with testicular cancer, a marked reduction in steps following chemotherapy occurred which recovered before the next course. Five of the patients with prostate cancer rapidly progressed, marked by a reduction in steps recorded. Three patients responded subjectively to therapy but pedometer readings showed a decline in steps which matched the progression in objective parameters. In this small group of patients, the pedometer score provided a simple useful objective measurement of their response to therapy.

**Effect of insulin and ketone bodies on tumour growth and host body weight in a cachexia model**

S.A. Beck & M.J. Tisdale

*CRC Experimental Chemotherapy Group, Pharmaceutical Sciences Institute, Aston University, Birmingham B4 7ET, UK.*

We have utilised the MAC16 adenocarcinoma of the mouse colon as an experimental model of human cachexia where weight loss occurs without a reduction in food or water intake. Weight loss produced by the MAC16 tumour is characterised by a loss of body fat and muscle dry weight, which increases in direct proportion to the tumour burden, and is associated with the presence of circulatory catabolic factors, which degrade host muscle and adipose tissue *in vitro*. Both insulin and 3-hydroxybutyrate inhibit the lipolytic and proteolytic activity *in vitro*, and thus a comparison has been made between the effects of daily insulin injections and a ketogenic diet on weight loss and tumour growth in the MAC16 model. Weight loss was significantly reduced by both a ketogenic diet (80% of calories supplied as medium chain triglyceride) and by administration of 20 U insulin $kg^{-1}$ $day^{-1}$ without an alteration in either water consumption or total calorie intake. At the end of the experiment the tumour weight in animals fed a ketogenic diet was significantly lower than those consuming the normal laboratory diet, while in animals treated with insulin the tumour weight was 50% greater than controls. The enhancement of tumour weight by insulin was prevented by the concurrent administration of sodium 3-hydroxybutyrate. Both carcass fat and muscle dry weight were elevated in animals fed the ketogenic diet or administered insulin when compared with controls. These results suggest that both insulin and ketone bodies are effective in prevention of weight loss in cancer cachexia, but that insulin administration may be associated with an enhanced tumour growth rate.

**A new method of screening for preclinical cervical cancer by analysing the glycoprotein content of cervical scrapes**

G.A. Turner, L. Arvanitakis, J.M. Monaghan & S. Thompson

*Department of Clinical Biochemistry, The Medical School, Newcastle upon Tyne, NE2 4HH; and Gynaecological Oncology Unit, Queen Elizabeth Hospital, Gateshead NE9 6SX, UK.*

Cervical cancer is a significant cause of female mortality. It has a clearly defined preclinical phase which can be detected by cytological examination of a cervical smear. Although a full call and recall system has now been introduced in an attempt to reduce the mortality rate, insufficient experienced cytologists are available to cope with the workload. If a biochemical method could be developed that could rapidly screen out all the negative specimens this could result in a 90% saving in manpower costs. The test described here may offer this possibility. This involves removing a cervical specimen using an endocervical brush and placing the cells and mucus into a close-fitting tube containing NP40 and Tris buffer. Glycoproteins are extracted from this solution using Sepharose-coupled wheat germ agglutinin and their composition analysed by SDS/PAGE and silver staining (Thompson *et al.*, *Clin. Chim. Acta*, 1987, **167**, 217). Extracts from 10 women who had normal smear cytology showed very consistent electrophoretic patterns. In contrast 10 women who exhibited various degrees of dyskaryosis showed different and more complex patterns; the most consistent changes were increases in components with $M_r$ of 40–45 kDa and 55–57 kDa and a reduction in components at 75–80 kDa. One of the components in the 55–57 kDa region has been tentatively identified as cytokeratin. These results suggest that negative and positive cervical cytology can be easily distinguished. As yet no qualitative or quantitative relationship has been found between the electrophoretic patterns and the degree of cytological abnormality.

**Increased calmodulin inhibition and cytotoxicity against the MCF-7 breast cancer cell line by iodinated derivatives of tamoxifen**

R. McCague, M.G. Rowlands, I.B. Parr, M. Jarman & P.M. Goddard

*Section of Drug Development, Institute of Cancer Research, Sutton SM2 5NG, UK.*

Since the discovery by Lam that tamoxifen is a competitive antagonist of the activation of cyclic-AMP phosphodiesterase by calmodulin, inhibition of calmodulin action has been suggested to contribute to the inhibition by tamoxifen (TAM) of hormone responsive breast cancer growth. Possibly, optimisation of calmodulin antagonism in agents that also bind to the oestrogen receptor (ER) might provide a strategy for the development of improved drugs for breast cancer treatment.

We have recently discovered that 4-iodotamoxifen (IT) (*E*-1-(4-(2-(dimethylamino)ethoxy)phenyl)-1-(4-iodophenyl)-2-phenyl-1-butene) has greater affinity for cytosolic ER than tamoxifen. Unlike 4-hydroxytamoxifen (OHT) where the increased ER binding contrasts with a decreased calmodulin inhibition, we now find that IT is a more potent calmodulin inhibitor than TAM and the iodinated analogue (IPT) with dimethylamino replaced by pyrrolidino is more potent still. $IC_{50}$ values for the inhibition of cyclic-AMP phosphodiesterase activity increase in the order IPT = 1.4 $\mu$M, IT = 2.3 $\mu$M, TAM = 6.75 $\mu$M, OHT = 19 $\mu$M. This order correlates with that of the cytotoxicity against MCF-7 cells in monolayer culture after exposure to drug for 24 h, a timespan insufficient to observe the antagonism of hormone mediated proliferation. Thus at 10 $\mu$M of drug, numbers of viable cells, as percentage of control were IPT = 29, IT = 29, TAM = 104, OHT = 129. The iodinated tamoxifen analogues did not show this increased cytotoxicity against the oestrogen independent Walker rat carcinosarcoma and L1210 murine leukaemia cells in culture where all the four antioestrogens gave $IC_{50}$ values between 22 and 34 $\mu$M.

The above results support the viewpoint that calmodulin could play a role in the inhibition by tamoxifen of oestrogen responsive tumour growth, and indicate a possible superiority of iodinated tamoxifen analogues in the treatment of hormone dependent breast cancers.

### Steroid hormones and receptors in human breast carcinoma

V. Ramalingam & P. Govindarajulu

*Department of Endocrinology, P.G. Institute of Basic Medical Sciences, Madras 600 113 and Department of Zoology, Tagore Arts College, Pondicherry 605 008, India.*

To evaluate the hormone dependency of human breast cancer, the measurement of oestrogen (ER) and progesterone (PR) receptors were undertaken. Circulating oestradiol and progesterone were also measured to correlate with the receptor status. Ninety-eight premenopausal and 80 post-menopausal subjects with carcinoma breast and 30 normal subjects were included. Premenopausal subjects were further classified into follicular, midcycle and luteal phases. Receptor determinations were performed by Scatchard analysis of dextran coated charcoal method in both the cytosol and nuclear fractions. Serum oestradiol was significantly elevated in 28% of premenopausal and 34% of post-menopausal subjects with carcinoma. However, serum progesterone was decreased only in the luteal phases of premenopausal subjects and unaltered in post-menopausal subjects. The percentage of ER positive subjects was 79 and 36 and that of PR positive subjects was 64 and 29 in the cytosol and nuclear fraction, respectively. Both pre-menopausal and post-menopausal subjects showed no difference in their receptor concentration while significant variations were observed among follicular, midcycle and luteal phases. No clear correlations were observed between serum hormones and their receptor status. Efforts are in progress with many more subjects to make a correlation among serum hormones, tissue hormones and their receptor status.

### High progesterone receptor levels in human breast cancer cells (ZR-75-1) adapted to growth under oestrogen free conditions

H.W. van den Berg, M. Lynch & J.H.J. Martin

*Department of Therapeutics and Pharmacology, The Queen's University of Belfast, Belfast BT9 7BL, UK.*

Under routine culture conditions in medium containing fetal calf serum associated oestrogens and the weakly oestrogenic pH indicator phenol red, ZR-75-1 human breast cancer cells express oestrogen receptor (ER) and progesterone receptor (PGR). PGR levels can be further induced (approximately 10-fold), by exposing cells to oestradiol, (10-9 M), for 5 days. Both tamoxifen and medroxyprogesterone acetate (MPA) are growth inhibitory. Short-term (1 week) culture of cells in medium lacking phenol red and containing dextran charcoal stripped serum resulted in a pronounced slowing of growth rate and PGR was undetectable. ER levels were unchanged and PGR could be induced by oestradiol treatment. Growth rate could be slowed further by tamoxifen treatment whilst MPA now stimulated growth as did oestradiol. Long-term (3 months) culture of cells in oestrogen-free conditions resulted in a return to a growth rate similar to that of cells grown under routine culture conditions. ER could no longer be detected using a whole cell exchange binding assay, while PGR expression had risen to a level normally associated with oestrogen induction. PGR could not be further induced by oestradiol treatment. Long-term oestrogen withdrawn cells retained tamoxifen sensitivity and were again sensitive to growth inhibition by MPA, but physiological concentrations of oestradiol were without effect. This phenotype has remained stable for two years.

Our data would be consistent with the proposal that ZR-75-1 cells adapt to growth in the absence of oestrogen by expressing ER which has lost the ability to bind oestradiol and is biologically active in the absence of the hormone.

### Epidermal growth factor receptor expression in primary breast tumour tissue

J. Lunec, S. Nicholson, J. Fennelly, C. Parker, G.V. Sherbet & A.L. Harris

*Cancer Research Unit, Medical School, University of Newcastle upon Tyne, Newcastle upon Tyne NE2 4HH, UK.*

Levels of epidermal growth factor receptor (EGFR) determined by ligand binding assays on primary breast tumour samples were previously shown to be inversely correlated with the presence of oestrogen receptors and to be an indicator of poor clinical prognosis. We have gone on to investigate the mechanisms which may determine EGFR levels in breast tumours.

Twenty-seven DNA samples and 19 RNA samples were probed with EGFR cDNA encoding the external, trans-membrane and part of the internal region of the receptor. The results were correlated with EGFR levels determined by ligand binding for the same tumours. Amplification of the EGFR gene was detected in 3 out of 11 cases for which EGFR levels were $>70\,\mathrm{fmol\,mg^{-1}}$ membrane protein. For all 16 samples showing $<70\,\mathrm{fmol\,mg^{-1}}$, no amplification was detected. Adjustment for non-random selection of samples indicated an overall incidence of ~3% for amplification of the EGFR gene. The degree of amplification was 5, 10 and 50-fold.

Analysis of RNA samples for EGFR mRNA revealed a complex relationship between gene copy number, transcript levels and the number of receptors. In some cases, high receptor levels were measured in the absence of either amplification or high levels of transcription, indicating translational or post-translational mechanisms for establishing high levels of receptor. The highest transcript levels were observed for two of the samples in which gene amplification was detected and these were associated with high levels of receptor. For the subset of cases for which EGFR $<100\,\mathrm{fm\,mg^{-1}}$, a significant correlation was found between receptor levels and EGFR mRNA, demonstrating some degree of transcriptional determination of receptor levels.

**Receptors for the neuropeptide growth factors bombesin, bradykinin and vasopressin recognise two classes of antagonist**

P.J. Woll & E. Rozengurt

*Imperial Cancer Research Fund, PO Box 123, Lincoln's Inn Fields, London WC2A 3PX, UK.*

Neuropeptides are increasingly recognised as growth factors, so their antagonists could be useful anti-proliferative agents. We have shown that bombesin/gastrin-releasing peptide (GRP), bradykinin and vasopressin are mitogens in Swiss 3T3 cells and each stimulates a rapid intracellular $Ca^{2+}$ flux. Their receptors are distinct as shown by (i) specific cell surface binding of $^{125}$I-GRP and $^{3}$H-vasopressin which is not inhibited by the other mitogens and (ii) the existence of ligand-specific antagonists (Leu$^{13}$-$\Psi$(CH NH) Leu$^{14}$) bombesin, (DArg$^{0}$,Hyp$^{3}$,Thi$^{5,8}$,DPhe$^{7}$)bradykinin and (Pmp$^{1}$, OMeTyr$^{2}$,Arg$^{28}$)vasopressin which are inactive against the other mitogens. In contrast, (DArg$^{1}$, DPhe$^{5}$DTrp$^{7,9}$,Leu$^{11}$) substance P reversibly inhibits ligand binding, $Ca^{2+}$ mobilisation and mitogenesis induced by GRP, bradykinin and vasopressin, but no other growth factors. We suggest that the receptors for these neuropeptides exhibit two binding domains. One is ligand-specific and is blocked by the ligand-specific antagonists. The second domain is common to the three receptors and is blocked by (DArg$^{1}$,DPhe$^{5}$, DTrp$^{7,9}$,Leu$^{11}$)substance P. Small cell lung cancer (SCLC) secretes many peptides and hormones including GRP and vasopressin. (DArg$^{1}$, DPhe$^{5}$, DTrp$^{7,9}$, Leu$^{11}$)substance P inhibits the growth of SCLC cell lines. We are testing the receptor model in SCLC cell lines, utilising measurements of intracellular $Ca^{2+}$ fluxes.

**The biological and the clinical significance of epidermal growth factor and transforming growth factor alpha in normal and malignant ovaries**

O.J. Owens & R.E. Leake

*Department of Biochemistry, Glasgow University, G12 8QQ, UK.*

Epidermal growth factor (EGF) and transforming growth factor alpha (TGF$\alpha$) were assayed in 65 samples by a radioimmunoassay method (The sensitivity of both assays is between 20 pg and 5 ng growth factor per 250 $\mu$l of tissue extract. The cross-reactivity is such that 10 $\mu$g of EGF will measure as 40 pg in a TGF$\alpha$ assay and vice versa). Seventeen samples were from normal ovaries, 6 were from benign ovarian tumours, 27 were from primary ovarian tumours, 3 from metastatic deposits in the omentum, 6 had primary tumours elsewhere and 6 were from second look operations (ovary 2, nodes 3 and omentum 1). TGF was found to be present in all normal ovaries and in the majority of ovarian tumours (85%), while EGF was present in 22% of normal ovaries and 38% of ovarian tumours. The amount of TGF$\alpha$ measured ranged from 195 to 1750 pg g$^{-1}$ of wet tissue in normal ovaries (mean 758 pg g$^{-1}$) while EGF ranged from 237 to 640 pg g$^{-1}$ (mean 375 pg g$^{-1}$). In primary ovarian tumours the value of TGF$\alpha$ ranged from 53 to 4372 pg g$^{-1}$ (mean 964 pg g$^{-1}$), and EGF ranged from 229 to 348 pg g$^{-1}$ (mean 203 pg g$^{-1}$). In summary we can conclude that TGF$\alpha$ is more frequently found than EGF in both benign and malignant ovaries and when present exists in greater quantities. The prognostic significance of these assays is yet to be established.

**Extractable transforming growth factor alpha and epidermal growth factor in cervical and endometrial cancer**

L. Carr & R.E. Leake

*Department of Biochemistry, Glasgow University, Glasgow G12 8QQ, UK.*

Transforming growth factor alpha (TGF$\alpha$) and epidermal growth factor (EGF) were extracted from malignant endometrial and cervical tissue by organic solvent extraction. The growth factors were measured using sensitive radioimmunoassays (range 20 pg to 5 ng growth factor in 250 $\mu$l tissue extract). The primary antibodies were highly specific, such that 10 $\mu$g EGF measured only 40 pg in the TGF$\alpha$ assay and vice versa. In cervix the median value for TGF$\alpha$ was 694 pg g$^{-1}$ tissue (range 206–14,702 pg g$^{-1}$ tissue). Of 13 cervix samples analysed only 3 were negative for TGF$\alpha$ whereas the majority (10) were negative for EGF (3 positive; range 82–599 pg g$^{-1}$ tissue). Endometrial cancer showed a similar pattern with 6 out of the 15 samples assayed as positive for EGF (median 759 pg g$^{-1}$ tissue; range 172–1204 pg g$^{-1}$ tissue). Only one sample was negative for TGF$\alpha$ (median 1259 pg g$^{-1}$ tissue; range 377–15,517 pg g$^{-1}$ tissue). Both growth factors are thought to act via the EGF-receptor. Tumours of the cervix have been shown biochemically and immunohistochemically to express the EGF-receptor whereas, the majority of endometrial tumours were shown to be EGF-receptor negative. Thus the site of action of the endometrial TGF$\alpha$ is, as yet, unclear. As the levels of the growth factor detected are very variable it will be interesting to determine the prognostic value of growth factor levels in both endometrial and cervical cancer.

**Regulation of epidermal growth factor receptor synthesis by ovine and human prolactin**

F. Rinaldi & R.E. Leake

*Department of Biochemistry, University of Glasgow, G12 8QQ, UK.*

A combination of ovine prolactin (1 $\mu$g ml$^{-1}$) and epidermal growth factor (EGF) (0.005 $\mu$g ml$^{-1}$) was shown to induce marked growth stimulation (250% increase over control after four days) in the human breast cancer cell line ZR-75-1. As the level of EGF receptor (EGF-R) is relatively low in these cells under control conditions (70 fmol mg DNA$^{-1}$ ml$^{-1}$), studies were carried out to determine the ability of ovine and human prolactin to induce EGF-R synthesis. Ovine prolactin (1 $\mu$g ml$^{-1}$) and human prolactin (40 ng ml$^{-1}$) were both shown to induce the synthesis of EGF-R over the same time course. Peak levels of EGF-R (320 fmol mg DNA$^{-1}$ ml$^{-1}$) were seen at four days after initial exposure to the hormone. This stimulation was completely abolished by non-toxic levels of cycloheximide (40 nm). Medium conditioned by prolactin activated cells was also shown to stimulate EGF-R synthesis in fresh ZR-75-1 cells. These results indicate that human prolactin induces EGF-R synthesis in human breast cancer cells through a paracrine mechanism. These observations may explain the suggestions in the literature that prolactin can promote breast tumour growth.

**Effects of human platelet-poor plasma (PPP) on the growth of breast cancer cells**

T. Simpson & H. Braunsberg

*Department of Chemical Pathology, St Mary's Hospital Medical School, London W2 1PG, UK.*

Human breast cancer progression may be determined by the concentrations of growth stimulatory (STIM) and inhibitory

(INH) hormones and growth factors, derived from blood or produced locally, and of receptors for these substances in and on the cells. The relative importance of autocrine, paracrine and endocrine effects remains to be established and we have focused on the latter.

Human breast cancer cells (MCF-7) were exposed to media containing charcoal-stripped cell free blood preparations at concentrations ranging from 1 to 20% v/v. Since platelets can release INH and STIM factors during clotting, and freezing of samples reduced STIM but not INH activity, we studied fresh PPP. With samples from 2 normal women, a patient with benign breast disease and 3 of 6 breast cancer patients, growth was maximal at 6% (v/v) PPP and declined at higher concentrations. Two PPPs from breast cancer patients produced maximal growth at 2% and two contained an additional STIM factor of low affinity and/or concentration.

Differences in patterns of blood-borne STIM and INH factors may, at least in part, account for variations in tumour growth rates. Their nature, concentrations and interactions with each other and with steroid hormones and other growth factors require full investigation.

### A novel endothelial cell growth factor from a human lung tumour cell line

C. Walker, R. Akhter & D. Pumford

*Clatterbridge Cancer Research Trust, J.K. Douglas Cancer Research Laboratories, Clatterbridge Hospital, Bebington, Merseyside L63 4JY, UK.*

Human lung cancer cell lines have been shown to produce a variety of growth factors some of which are known to be angiogenic *in vivo* (Soderdahl *et al.*, *Int. J. Cancer*, 1988, **41**, 636). The 1PT cell line, from a poorly differentiated bronchial carcinoma, grows at a confluent density for several months in serum-free DMEM. Medium from this cell line has been shown to be angiogenic *in vivo* (Kumar *et al.*, *Int. J. Cancer*, 1983, **32**, 461) and to stimulate migration, chemotaxis (Daniel *et al.*, *Cell Biol. Int. Rep.*, 1987, **11**, 235) and proliferation of sparse cultures (Walker *et al.*, *J. Cell Sci.*, 1987, **87**, 739) of human umbilical vein endothelial (HUV) cells. It supports their clonal growth and long term serial cultivation and also stimulates the growth of microvascular endothelial cells and fibroblastic cell types but not certain epithelial tumour cell lines. Characterisation of the endothelial mitogen has shown that it is a heat-labile, acid-stable, proteinaceous material, which does not bind to heparin-sepharose (Walker *et al.*, 1987). A variety of techniques has failed to liberate an active fraction of MW<30 kD suggesting that the factor is not bound to a high molecular weight carrier molecule or present in aggregated form. It does not bind to immobilised lentil or wheat germ lectins, but does have affinity for glycine max lectin and is eluted from this by *N*-acetylgalactosamine, indicating that it is a glycosylated protein. Although we have not yet purified this factor, our results so far suggest that it differs from other previously described tumour-derived endothelial mitogens.

### Cell type-dependent modulation of ether lipid-induced changes in calcium homeostasis by a phorbol ester

C.M. Lazenby, M.G. Thompson & J.A. Hickman

*CRC Experimental Chemotherapy Group, Pharmaceutical Sciences Institute, Aston University, Birmingham B4 7ET, UK.*

The ether lipids represent a new class of membrane-active antitumour agents. One of these, SRI 62-834, was reported by us to bring about a concentration-dependent increase in the intracellular $Ca^{2+}$ concentration of HL-60 human myelomonocytic leukaemia cells which was inhibited by pre-treatment with 12-*O*-tetradecanoylphorbol-13-acetate (TPA) (Thompson & Hickman, *Biochem. Soc. Trans.*, 1988, **16**, 278). This led us to suggest that its effect on the membrane were not those of a detergent-like activity. Treatment of $7.5\times10^6\,ml^{-1}$ K562 human erythroblastic leukaemia cells with 60 $\mu$M SRI 62-834 (below the $IC_{10}$) for 10 min resulted in a smooth rise in intracellular $Ca^{2+}$ from $74\pm4.5$ nmol to $167.5\pm17.6$ nmol ($n=3$), as monitored by the fluorescent calcium indicator Quin-2. Pretreatment of these cells with 100 nmol TPA for 10 mins reduced the $Ca^{2+}$ rise by 78%. Treatment of $7.5\times10^6\,ml^{-1}$ V79 hamster fibroblast cells, grown in suspension culture, with 35 $\mu$M SRI 62-834 brought about a sharp rise in intracellular calcium which accompanied a loss of membrane integrity: the effects of 35 $\mu$M SRI 62-834 on this cell line were not inhibited by TPA pretreatment. We suggest that SRI 62-834 may modulate membrane function in distinctive ways depending on the cell type.

### Aromatase inhibition by 4-hydroxyandrostenedione in advanced prostatic cancer

J.H. Davies, M. Dowsett, R.C. Coombes & R.J. Shearer

*Departments of Urology and Medical Oncology, St Georges Hospital; and Biochemical Endocrinology, Royal Marsden Hospital, London, UK.*

Studies have shown the benefit of aminoglutethimide in patients with advanced prostatic cancer. However, its mechanism of action is not clear. Although it inhibits a range of other steroidogenic enzymes, its most potent action is on the aromatase enzyme which is the final enzyme in the biosynthesis of oestrogens and this may be its mode of action. 4-hydroxyandrostenedione (4-OHA) is a more selective steroidal aromatase inhibitor. We have conducted a clinical trial of this drug in an attempt to define the role of aromatase inhibition and of oestrogens in prostate carcinoma. In a phase I study, 16 patients, who had relapsed following orchidectomy for advanced prostatic carcinoma, were given 500 mg 4-OHA intra-muscularly weekly. Complete subjective responses (CSR) were seen in 3 (18%) and partial subjective responses (PSR) in 8 (50%) patients. Average duration of response was 10 weeks. Objectively, 4 (25%) patients had a fall of >50% in serum acid phosphatase. Androgen levels did not change but oestradiol levels fell significantly in 5 of 8 patients in which it was measured. A transient increase in bone pain occurred in 5 (31%) patients. A phase II study is in progress with 20 patients entered to date, receiving the same dose of 4-OHA. CSR has been observed in 7 (35%) and PSR in 5 (25%) patients. Prostatic acid phosphatase and prostate specific antigen fell by >50% in 3 patients who responded. Aromatase inhibition offers a new approach to the management of advanced prostatic carcinoma.

### Measurement of nuclear pleomorphism and DNA content in metastatic variants of the B16 murine melanoma using image analysis techniques

G.V. Sherbet & M.S. Lakshmi

*Cancer Research Unit, The Medical School, University of Newcastle upon Tyne, Newcastle upon Tyne NE2 4HH, UK.*

Tumours are notable for the variability in the expression of differentiated characters, tissue architecture and cellular morphology. Nuclear pleomorphism is one of the criteria used in the histological grading of tumours, but quantitative objective measurement of pleomorphism has not been attempted. In the present work, we have used image processing techniques to measure nuclear pleomorphism (NPM), integrated nuclear density (IND) and nucleocytoplasmic ratio (NCR) in the cell line B16-F1, which is poorly metastatic to the lung, and B16-BL6, which metastasises extensively to the lung. A third cell line, ML8, isolated from metastatic tumour from lungs of an animal bearing a subcutaneous BL6 tumour, was also studied. This was more malignant than its corresponding primary line, the BL6.

The Joyce Loebl Magiscan MD was used to measure NPM, IND and NC in cytospin preparations of cells and sections of tumours produced by s.c. injection of cells into syngeneic C57BL/6 mice, both stained with haematoxylin and eosin. The BL6 cells showed greater pleomorphism than their metastatic counterparts ML8. NPM of F1 cells was intermediate between BL6 and ML8. No appreciable differences were noticed in tumour tissues. The NCR and IND patterns of the three variant cell lines were similar, but the profile of IND, which represents DNA content, was markedly different in the tumours. F1 and BL6 tumours had one major peak (IND = 175) but ML8 had an additional peak (at IND = 425). These data suggest that measurement of integrated nuclear densities may be more appropriate than nuclear pleomorphism as a feature of malignancy.

Supported by the North of England Cancer Research Campaign.

### The development of a model of metastatic bladder cancer using the immune-suppressed mouse

K.M. Joy, C.A. Parry & J. Chowaniec

*Department of Histopathology, University College and Middlesex School of Medicine, London W1P 7LD, UK.*

Our immune-suppressed (i/s) mice permit the growth of s.c. inoculated human bladder cancer cell lines; the resultant tumours are clinicopathologically similar to the primaries from which the cells were derived (Joy *et al.*, *J. Pathol.*, 1981, **134**, 335). Some of the tumours (e.g. produced by EJ cells) metastasise in the mice, but they do not do so reproducibly.

Several cell lines derived from chemically induced preneoplastic or neoplastic rat bladders are likewise tumorigenic in our i/s mice, producing transitional or squamous cell carcinomas which often metastasise, but with variable frequency and onset time. However, an agar-cloned subline, RU-CL2, produces transitional cell carcinomas that rapidly invade the subcutaneous host tissues, including nerve sheaths and skeletal muscle, and reproducibly metastasise haematogenously to the lungs and to the distal lymph nodes. As early as 35d post-inoculation, metastases resembling the primary tumours are histologically detectable in the lungs of some i/s mice; by 50d, multiple metastases are present in the lungs of all animals inoculated.

RU-CL2 is not tumorigenic in immunocompetent mice. Substantial tumours form when RU-CL2 cells are injected into weanling syngeneic rats, but these elicit a vigorous host response and hence soon regress.

Various animal models of tumour metastasis currently exist; some, however, involve tail-vein injection of cells, thus by-passing early events in the metastatic cascade, and relatively few concern metastasis of epithelial cell types. The use of RU-CL2 cells in i/s mice offers a model with which to study factors controlling invasion and metastasis of epithelial tumours. Because of the relatively short onset time for 'spontaneous' tumour metastasis, this model may also prove useful for assessment of cancer chemotherapy modalities.

### Influence of stromal cells on the phenotype of alveolar carcinoma cells *in vitro*

V. Speirs & R.I. Freshney

*CRC Department of Medical Oncology, University of Glasgow, Glasgow, UK.*

Production of pulmonary surfactant (PS) is necessary for normal lung function and its presence is indicative of normal differentiated lung epithelium. The human alveolar carcinoma A549 has been found to synthesise and secrete PS in culture. The aim of this study was to determine the relationship between stromal cells and A549 in the induction of PS production by glucocorticoids following previous observations by Smith (*Biochim. Biophys. Acta*, 1984, **793**, 297; *J. Biol. Chem.*, 1986, **261**, 2179). Cells were grown in filter wells in the presence of either fibroblast conditioned medium (FCM) or fetal lung fibroblasts (MOG-LF113) either in direct contact or in high density coculture $\pm 0.25\,\mu M$ dexamethasone (DX). PS was measured by incorporation of 3H choline into disatured phosphatidyl choline. DX was found to cause a 5-fold increase in PS secretion in cells cultured alone on filter wells. Cells cultured on a conventional plastic substrate showed only a 1.5-fold increase, while those cultured in direct contact showed even higher levels of PS, a 7-fold increase in the absence of DX and a 2.5-fold increase in the presence of DX. Cocultures with A549 growing on the filters and fibroblasts on the bottom of the dish showed similar increases, implying that cell contact is not important for PS production. This was confirmed using FCM where there was a doubling in PS after 48 h incubation. We have partially purified a peptide from FCM of m.wt. 10–30 kDa and pI 8.7. It appears to be heat and alkali stable but unstable to treatment with acid, trypsin or pronase. Incubation of A549 cells with this factor can stimulate PS production by some 3-fold. Future studies will attempt to characterise this factor further and determine its effects on the growth of A549 xenografts in nude mice.

### The NX002 cell line – a new model system to study differentiation in lung squamous carcinoma

S.P. Langdon, G.J. Rabiasz, L.E. Anderson, A.A. Ritchie & J.F. Smyth

*ICRF Medical Oncology Unit, Western General Hospital, Edinburgh, UK.*

We have derived a new model system to study lung squamous differentiation. Induced differentiation has been proposed as a strategy to treat malignant disease (Sartorelli, *Br. J. Cancer*, 1985, **52**, 293) and since chemotherapy, at present, is relatively ineffective for the treatment of lung squamous carcinoma, induced differentiation might represent a possible approach.

The NX002 cell line was derived from a poorly differentiated squamous xenograft in its 20th passage which in turn originated from the primary tumor of a patient with lung squamous carcinoma. Its morphology has remained constant over 30 passages.

Terminal differentiation (TD) in normal keratinocytes is characterised by the attainment of the capacity to form cornified cell envelopes and also by the production of involucrin, a protein incorporated into the cornified envelope at TD. Both these markers are present in the NX002 cell line. Cornified envelopes are observed after cells have been incubated in 1.2 M NaCl for 4 hours and then treated with 2.5% SDS. Involucrin was identified immunohistochemically by the indirect immunoperoxidase method. At early confluence approximately 50% cells have the capacity to form cornified envelopes and 15% are positive for involucrin. In the NX002 xenograft, areas of squamous differentiation (cells undergoing keratinisation) were positive for involucrin.

This model provides an *in vitro–in vivo* system to study regulation and modulation of lung squamous differentiation.

**Small cell lung cancer cluster 1 antigens are diverse and inducible in non-SCLC cell lines after sodium butyrate treatment**

F.G. Hay, L.W. Duncan, S.P. Langdon & R.C.F. Leonard

*Imperial Cancer Research Fund Medical Oncology Unit, Western General Hospital, Edinburgh, UK.*

The Cluster 1 and Cluster 1-associated antigens were reported (Souhami *et al.*, *Lancet*, 1987, 325) to be a locus or loci of interest characteristic of the small cell [SCLC) subtype of lung cancer. A group of MoAbs defines these clusters by reacting strongly with SCLC tumour cells yet showing weak or negative reactions with other lung cancer cells and most other epithelial tumours. We have previously reported however the presence of this group of antigens in well differentiated ovarian tumour cell lines (Langdon *et al.*, *Lung Cancer*, 1988, **4**, 62). In further studies the expression of two Cluster 1 antigens (123C3 and 123A8) could be induced in three lung adenocarcinoma cell lines (H23, H125 and A549) using sodium butyrate, an agent capable of inducing differentiation in many systems (Langdon *et al.*, *Br. J. Cancer*, 1988, **58**, 247). Expression of these markers by non-SCLC cells may indicate that some members of the Cluster 1 and 1A group of antigens are not just confined to SCLC, but may be general markers of differentiation in epithelial tumour cells. We have examined the effects of sodium butyrate on the expression of all Cluster 1 and 1A antigens in 3 lung adenocarcinoma cell lines, 6 ovarian tumour cell lines, 3 colorectal tumour cell lines and 4 SCLC lines. In the untreated group the MoAbs had activity only with the SCLC lines and well differentiated ovarian lines whereas, after 4 days exposure to sodium butyrate, Cluster 1 and 1A antigens were detected heterogenously in all of the above cell lines. After treatment the SCLC lines showed enhancement of Cluster 1A antigens whereas the other currently identified Cluster 1 antigens did not change. The data suggest that the Cluster 1 antigens are diverse in expression and in function.

**Development from murine embryo neural crest of cell lines sharing some characteristics with small cell lung cancer (SCLC)**

M. Clay, C.M. Steel, F.G. Hay, A. Lessells & R.C.F. Leonard

*MRC Human Genetics Unit, Imperial Cancer Research Fund Medical Oncology Unit; and Pathology Department, Western General Hospital, Edinburgh, UK.*

With the aim of developing transfection recipients for human SCLC DNA, we identified a novel alternative to the classic NIH3T3 cell lines by establishing cell lines from explanted mouse embryo neural crest. The neural crest tissue from eight-day mouse embryos was micro-dissected and cultured in an F10 based media. A polycolonal subline grew out and survived thirty passages over eleven months; and it presents a range of distinct morphologies which persist despite being cultured in conditioned medium which normally prevents differentiation. Five monoclonal colonies, each contact-inhibited, were eventually cloned and injected into immune-deprived mice. All five have produced tumour growth although one has a very slow growth rate. Two of the five have been maintained in Iscove's serum free medium suggesting that they may be producing autocrine growth factors. Characterisation of the cell lines has confirmed their non-fibroblastic nature; electron microscopy studies have shown cell junctions, cell processes with possible neurotubule formation and some neuro-secretory granules compatible with a neural origin. Cell surface markers for epithelial antigens and neural antigens are both positive and the cells contain low molecular weight cytokeratins and neuro-filaments. These tumorigenic cell lines will be probed for the presence of N-*myc*, c-*myc* and L-*myc* which we have found characteristically expressed in SCLC cell lines. Efforts to enhance the tumorigenicity in mice will be made using segments of human SCLC DNA for transfection.

**Characteristics and interrelationship of two cell lines derived from a bronchial carcinoma**

S. Wright-Perkins, M. Daniel & C. Walker

*Clatterbridge Cancer Research Trust, J.K. Douglas Cancer Research Laboratories, Clatterbridge Hospital, Bebington, Merseyside L63 4JY, UK.*

It has been suggested that carcinomata of both small cell (SCLC) and non-small cell (NSCLC) types arise from a common stem cell. Changes in phenotype of lung cancer *in vivo* and *in vitro* have been reported. The 1PT cell line, derived from a poorly differentiated bronchial carcinoma, consists of large polygonal cells which grow both in monolayer and in suspension; unlike other NSCLC lines, confluent monolayers continue to proliferate in serum-free DMEM unsupplemented with the factors essential for SCLC cells. A variant line derived from this grows in monolayer as compact plaques or rods of smaller phase-dense cells which unlike the 1PT cells can be grown in serum-free DMEM from clonal density; in suspension, these cells form three-dimensional structures. DNA histograms obtained by flow cytometry show that the 1PT line is hyperdiploid, while the variant is tetraploid. Immunocytochemical techniques have been used to test for the presence of various epithelial and lung-associated antigens. Continued cultivation of the variant line results in a cyclical production and loss of 1PT-like cells. Clonal lines of 1PT have been established in order to investigate whether these can conversely give rise to variant-type cells. The characteristics of these two lines suggest their potential usefulness for the investigation of the interrelationship of various malignant pulmonary epithelial cells.

**Retinoic acid inhibits the transformed phenotype *in vitro* of metastatic and non-metastatic bladder cancer cell lines**

A. Jones, K. Joy & J. Chowaniec

*Department of Histopathology, School of Pathology, University College and Middlesex School of Medicine, London W1P 7LD, UK.*

The ability of all-*trans*-retinoic acid (RA) to inhibit the clonogenicity in soft agar of metastatic and non-metastatic rat and human bladder cancer cell lines was investigated. Retinoic acid reproducibly inhibited anchorage-independent (AI) growth of the well differentiated, papillary, human bladder cancer cell line RT112 which is non-metastatic in immunodeficient mice, and of the poorly differentiated, rat bladder carcinoma cell line RU-CL2 which consistently metastasises in such animals.

For both cell lines, RA-induced inhibition of AI growth was dose-dependent, the $ID_{50}$ being approx. $10^{-9}$ M, though doses as low as $10^{-11}$ M RA were effective. The increased inhibition at doses above $10^{-8}$ M RA was not directly proportional to dose and, at these levels, the effect of RA toxicity could not be excluded. However, even at $10^{-7}$ M RA, the inhibition of clonogenicity in agar was dependent upon the continuous presence of retinoid, since removal of exposure of cells to RA resulted in a loss of RA-induced inhibition. Similarly, dose-dependent RA-induced morphological changes in monolayer cultures of RU-CL2 were reversible. By contrast, the morphology of RT112 cell monolayers was unaffected by the presence of RA.

The inhibition of AI growth of both cell lines was RA-specific, since the synthetic retinoid, 4-hydroxyphenylretinamide (4HPR) was ineffective.

Whereas AI growth both of metastatic and non-metastatic cell lines was inhibited by RA, the morphology of only the metastatic line RU-CL2 was sensitive to RA. This suggests that RA modulation of AI growth is independent of the effects of RA on cell morphology, consistent with the proposal that two nuclear RA receptor genes transactivate the transcription of different target genes (Brand *et al.*, *Nature*, 1988, **332**, 850).

**Humoral hypercalcaemia of malignancy factor and renal carcinoma**

C.J. Gallagher, J. Mosley, R.T.D. Oliver, J. Martin & M. Horton

*Departments of Medical Oncology, Histology, The London Hospital; and Department of Exp. Haematology, St Bartholomew's Hospital, London, UK.*

Renal carcinoma is associated with the humoral hypercalcaemia of malignancy (HHM) syndrome in up to 30% of patients who have a worse prognosis than those remaining normocalcaemic.

Parathyroid hormone-related protein (PTHrP) has recently been identified as one of the factors capable of causing HHM. We have investigated the presence of PTHrP in normal and malignant renal tissues by imunoperoxidase staining of paraffin embedded sections using a rabbit antiserum to synthetic peptides unique to PTHrP. Ten renal carcinomas have been examined of which 7 were associated with hypercalcaemia and 3 were normocalcaemic. Two fetal kidneys of 23 and 40 weeks gestation were also examined. Positive staining for PTHrP was found in all renal carcinoma sections irrespective of the presence or absence of hypercalcaemia. PTHrP was also found in the adjacent normal kidney and in sections of fetal kidney. PTHrP was present in the epithelial cells of the normal renal tubules and collecting ducts. In the fetal kidney a similar pattern was seen with staining of both proximal and distal tubules even at 23 weeks gestation. Some cells in each of the carcinomas stained although there was considerable variation between tumours and between cells within any tumour. The presence of PTHrP in renal carcinomas is consistent with their histogenesis from renal tubular epithelium as has been previously shown by cell surface antigens. Further investigation is needed to define how tumour derived PTHrP becomes hypercalcaemogenic.

**Novel methods for increasing the number of clones expressing sensitivity to a cytotoxic agent in a tumour cell population**

K. Maynard & B.T. Hill

*Laboratory of Cellular Chemotherapy, Imperial Cancer Research Fund, Lincoln's Inn Fields, London WC2A 3PX, UK.*

Studies of the mechanisms associated with cellular sensitivity to antitumour drugs may be useful in devising methods of circumventing or overcoming drug resistance. Instead of attempting to assay the drug sensitivity of thousands of clones in a cell population in order to identify a small number of sensitive clones, we have used two new methods to increase the proportion of sensitive clones in a human tumour cell population. The first procedure, a modification of a method originally described by Sato and Shiomi (*Somat. Cell Genet.* 1979, **5**, 193), used cisplatin-treated adenovirus as a lethal probe to obtain cisplatin-sensitive clones from an ovrian carcinoma cell line (SK-OV-3). These clones showed a range of two to seven-fold increased sensitivities to cisplatin and at least some were deficient at reactivation of cisplatin-treated adenovirus. The second procedure involved the development of a new method using temperature-sensitive adenovirus to increase the number of doxorubicin-sensitive clones in a cell population. These clones showed a three to six-fold increase in doxorubicin sensitivity. Some clones showed cross-sensitivity to other antitumour agents such as etoposide. Characterisation of these doxorubicin-sensitive clones may lead to the identification of enzymes involved in maintaining the normal level of cellular resistance to doxorubicin. This method using temperature-sensitive adenovirus may prove generally useful in isolating clones more sensitive to DNA synthesis inhibitors such as hydroxyurea and methotrexate.

**The use of genetic marking to assess the interaction of sensitive and multidrug-resistant cells in mixed culture**

C. Bradley & J.D. Pitts

*CRC Department of Medical Oncology, University of Glasgow; and Beatson Institute for Cancer Research, Glasgow, UK.*

There is evidence for the transfer of drug resistance from resistant to sensitive cells in culture. Nitrosourea resistance is transferred between cells in spheroids (Tofilon *et al.*, *Science*, 1984, **226**, 864). Ouabain resistance is transferred in monolayer cultures by the rapid diffusion of $K^+$ and $Na^+$ ions through gap junctions (Corsaro & Migeon, *Nature*, 1977, **268**, 737). The membrane pumps in the ouabain resistant cells maintain physiological ion concentrations in the cytoplasms of both cell types. It seems possible, therefore, that other forms of drug resistance which are dependent on the cytoplasmic concentration of small ions or molecules should be transferred between cells joined by gap junctions. We have studied the behaviour of normal ($CHO\text{-}K_1$) and multidrug-resistant ($Adr^r$) Chinese hamster cell lines which have been shown to communicate through gap junctions. The $CHO\text{-}K_1$ cells have been genetically marked by transfection with a bacterial $\beta$-galactosidase gene. This enzyme produces a blue product with appropriate substrate and allows identification of the marked cells. Monolayers of cells ($CHO\text{-}K_1$ alone, $Adr^r$ alone and a 50:50 mixture) were exposed in multiwell dishes to adriamycin or mitozantrone for 24 hours, then plated out at 500 cells per dish and a colony-forming assay performed. The number of blue and unstained colonies from the mixed culture could be counted separately to assess the individual response of the two cell types. In the individual assays $Adr^r$ is 100× more resistant to adriamycin and 20× more resistant to mitozantrone than $CHO\text{-}K_1$. However, in mixed culture the chemosensitivity of the two cell lines remains unchanged with no evidence of transfer of anthracycline resistance in this system.

### MCF-7 cells express differential drug responses and mechanisms of resistance following either fractionated X-irradiation or drug exposure *in vitro*

R.D.H. Whelan, L.K. Hosking & B.T. Hill

*Cellular Chemotherapy Laboratory, Imperial Cancer Research Fund, London WC2A 3PX, UK.*

Drug resistant sublines of the MCF-7 human breast carcinoma cell line have been used to establish whether different mechanisms are expressed or predominate depending on how drug resistance was induced or selected. We have therefore developed a series of vincristine-resistant MCF-7 sublines following fractionated X-irradiation or intermittent drug exposure *in vitro*. Drug-selected sublines expressed a 5–10-fold resistance to vincristine which could be partly reversed with verapamil and exhibited a pattern of cross-resistance to other antitumour agents consistent with the classical multidrug resistance phenotype. This pattern of reduced drug responses was associated with impaired drug accumulation and over-expression of a 170 kDa plasma membrane glycoprotein (P-glycoprotein), resulting from some gene amplification. In contrast, MCF-7 cells which exhibited a 4-fold resistance to vincristine following exposure to fractionated X-irradiation, did not express cross-resistance to adriamycin and had no detectable alteration in drug transport or over expression of P-glycoprotein. Verapamil did not potentiate the cytotoxicity of VCR in this subline. In addition, X-ray pretreated cells showed no modification in glutathione content or glutathione related enzyme activities, whereas glutathione S-transferase and peroxidase were significantly elevated in drug-selected resistant MCF-7 cell lines. These data indicate that different mechanisms of resistance may operate or predominate depending on how resistance was derived.

### Anthracycline cross-resistance and drug accumulation in a multidrug resistant human lung cancer cell line which does not express P-170 glycoprotein

H.M. Coley, P.R. Twentyman, P. Workman & J.G. Reeve

*MRC Clinical Oncology and Radiotherapeutics Unit, Hills Road, Cambridge CB2 2QH, UK.*

The adriamycin (ADM)-resistant variants of the human lung cancer cell lines NCI-H69 and COR-L23 show the multidrug resistant (MDR) phenotype, being cross-resistant to colchicine, vincristine and VP16. The MDR variant of small cell line H69 (H69/LX4) shows amplification and hyper-expression of MDR-1 gene encoding P-170 whereas the MDR variant (COR-L23/R) of large cell line COR-L23 does not. We have previously identified a number of anthracyclines which retain high activity in MDR cells (including H69/LX4), and have now determined the activity of these agents in COR-L23/R. The agents studied were aclacinomycin A (ACL), 4′-deoxy-4′-iodo ADM (iodo-ADM) and Ro 31-1215. These analogues have specific modifications at the 9 position of the A ring and/or the sugar residue. Relative degrees of resistance (i.e. $ID_{50}$ resistant line/$ID_{50}$ parent line) for H69 were ADM = 150, ACL = 5.8, iodo-ADM = 18.9 and Ro 31-1215 = 12.4, whereas the results for COR-L23 were ADM = 15, ACL = 1.2, iodo-ADM = 1.8, and Ro 31-1215 = 4.0. Both H69/LX4 and COR-L23/R were defective in ADM accumulation with respect to their parent lines. For H69/LX4 the ADM content was 30% of the parent and the corresponding result for COR-L23/R was approx. 65%. We are currently assessing the accumulation of the anthracycline analogues in the COR-L23 cell lines, and the effects of verapamil on chemosensitivity and drug accumulation. It appears that the presence of P-170 is not necessary for the drug cross-resistance profile and defective drug accumulation associated with MDR.

### Antisense oligonucleotides complementary to the *mdr1* gene (P-glycoprotein)

C. Clugston & R. Brown

*CRC Department of Medical Oncology, Beatson Institute, Glasgow, UK.*

Elevated expression of the *mdr1* gene, encoding the P-glycoprotein, increases the resistance of cells to several naturally occurring chemotherapeutic agents, including adriamycin. High expression of the *mdr1* gene has also been implicated in resistance of tumours to chemotherapy. In order specifically to inhibit expression of the *mdr1* gene we have synthesised complementary oligonucleotides (ONDs) to the *mdr1* RNA. The choice of ONDs was based on computer modelling of secondary structure of the *mdr1* RNA and searches for other possible cross-hybridising RNA species. Adriamycin resistant MCF-7 cells, which have elevated *mdr1* expression, are being used as the recipient cell line. ONDs have been synthesised with phosphodiester linkage or phosphorothioate linkage and their stability and efficiency of uptake are being compared. Heat inactivation of the serum increases the stability of the ONDs by more than 10-fold. DEAE dextran, polybrene and chloroquine did not increase stability or were too cytotoxic to the cells. Sensitivity of the cells to adriamycin is being measured using a short-term assay, the MTT assay. At present no significant increase in sensitivity of the cells has been detected but there are still considerable problems to be overcome with the intracellular stability of the ONDs and in ensuring sufficient uptake to inhibit *mdr1* expression.

### Response of chemically and virally induced mouse skin tumours to doxorubicin

N. Keith, P.J. Mee & R. Brown

*CRC Department of Medical Oncology, Beatson Institute, Glasgow, UK.*

Tumours can be induced in mice by treatment with chemical initiators (dimethyl benzanthracine; DMBA) and promoters (12-*O*-tetradecanoyl-phorbol-13-acetate; TPA). More than 90% of these DMBA/TPA induced tumours have the same specific A-T mutation at codon 61 in the cellular Ha-*ras* gene (Quintanilla *et al.*, *Nature*, 1986, **322**, 78). Tumours are also induced if the DMBA initiation is replaced by topical application of Harvey murine sarcoma virus directly to the skin (Brown *et al.*, *Cell*, 1986, **46**, 447). We have examined the response of these chemically and virally induced papillomas to the widely used chemotherapeutic agent, doxorubicin. We observe a greater regression frequency of virally induced, than chemically induced, papillomas after a single injection of doxorubicin. Eleven weeks after a single intravenous injection of $10\,mg\,kg^{-1}$ doxorubicin 95% of the virally induced papillomas have regressed but only 20% of those chemically induced. A larger regression frequency of chemically induced papillomas (73%) is observed after repeated injections of doxorubicin ($5\,mg\,kg^{-1}$ once per week for 4 weeks). Both chemically induced and virally induced papillomas show a small number of tumours which do not regress, even after repeated doxorubicin injections. At present we are analysing these doxorubicin resistant tumours for mutations of H-*ras* genes and for expression of drug resistance genes: P-glycoprotein (*mdr1*) and the acidic form of glutathione S-transferase (gst-pi).

**Cross-resistance associated with elevated cellular glutathione levels occurs with melphalan and photons but not melphalan and neutrons**

R.A. Britten, H.M. Warenius, R. White, J.A. Green & Browning

*CRC Department of Radiation Oncology and Department of Clinical Biochemistry, Clatterbridge Hospital, Wirral, UK.*

Cross-resistance to X-rays associated with elevated glutathione (GSH) levels following pretreatment with alkylating agents has been demonstrated in animal and human cell lines. Also we have shown a 10-fold ($P>0.001$) elevation of GSH prospectively in a series of 30 ovarian carcinoma treated patients by alkylating agents and cisplatin. Raised glutathione levels may thus be clinically important as a possible cause for the failure of combined sequential chemotherapy/radiotherapy regimes. Fast neutrons with less dependence on peroxyl radical formation for their effects might also be less affected by elevated GSH than are photons. We thus tested the effects of photons and neutrons on our melphalan-sensitive and -resistant OAW42 ovarian carcinoma cell lines. The melphalan-resistant subline OAW42/MER was 2-fold more resistant to melphalan than the parental OAW42 cells. Depletion of GSH by BSO potentiated melphalan cytotoxicity by 2–5-fold. The chemoresistant OAW42/MER cells were 1.75-fold more resistant to photon irradiation than OAW42 cells, BSO potentiated the lethality of photon irradiation by 17% in OAW42/MER cells, but did not have any effect in the parental OAW42 line. OAW42/MER cells were not cross-resistant to neutrons. We conclude that cross-resistance to photon irradiation exists in OAW42 cells resistant to alkylating agents. Neutron therapy is equally effective in both resistant and sensitive cells, and should possibly be considered as the appropriate radiation modality in neoadjuvant chemotherapy/radiotherapy protocols.

**Changes in glutathione metabolism following exposure to alkylating agents in human ovarian tumour biopsies**

R.A. Britten & J.A. Green

*CRC Department of Radiation Oncology, Clatterbridge Hospital, Wirral L63 4JY, UK.*

The glutathione (GSH)/glutathione-S-transferase (GST) dependent detoxification process has been shown to have an important role in alkylating agent resistance. Modulation of melphalan cytotoxicity has been achieved in animal, and human tumour cell lines (Green *et al.*, *Cancer Res.*, 1984, **11**, 211). Following confirmatory work on the importance of this mechanism in the human ovarian tumour OAW42 line in this laboratory, and the demonstration of a strong correlation between GSSG and GST levels ($r=0.9$), GSH and GST activity have been studied prospectively in fresh human ovarian tumour biopsies to determine their clinical relevance. This study has accrued 50 biopsies from 39 patients, with sequential data available on 5 patients. GSH levels in patients who had received prior chemotherapy showed a 10-fold elevation ($P<0.001$), over those previously untreated. The increase in GSH levels was directly proportional to the prior cumulative dose of alkylating agent received in the sequential biopsies. Within 10 weeks of the cessation of drug exposure GSH levels had fallen to a basal level, above that found in untreated patients, which persisted for up to 25 months. Changes in total GST activity following chemotherapy did not demonstrate a consistent pattern, and GST sub-unit analysis is currently being conducted on these samples. This finding suggests that a major component of the adaptive cellular response to alkylating agents may be partially reversible induction of GSH levels, concurrent with tumour cell subpopulation selection.

**Does aphidicolin glycinate reverse cisplatin resistance?**

J. Coffey & J.R.W. Masters

*University College London, Urological Oncology Unit, St Pauls Hospital, 24 Endell St, London WC2H 9AE, UK.*

Aphidicolin specifically reversed cisplatin resistance *in vitro* in a human ovarian cancer cell line (Ozols *et al.*, *AACR*, 1988, **29**, 526), and has entered phase I clinical trial. To confirm this observation we compared the cytotoxicities of aphidicolin glycinate (AG) and cisplatin (CP) alone and in combination against a cisplatin-resistant human bladder cancer cell line (RT112-CP) and its sensitive parent (RT112). Both cell lines were maintained under identical culture conditions and drug sensitivities were measured after a 6 day exposure using the MTT assay. The resistant cell line required approximately three times as much AG or CP to reduce cell numbers by 50%. A range of concentrations of CP were combined with three concentrations of AG: one non-toxic, one marginally toxic (reducing cell numbers by <10%) and one causing a 20–30% reduction. The non-toxic concentration of AG did not modify CP toxicity. However, the two higher concentrations reduced survival by slightly more than would be expected if the toxicities were additive and independent. For example, $500\,ng\,ml^{-1}$ CP reduced the survival of RT112-CP to 49%, $40\,ng\,ml^{-1}$ AP to 68% and the combination to 25% (expected 33%). In contrast to Ozols *et al.* (1988), AP increased the toxicity of cisplatin to both parent and resistant lines to the same small degree. Thus, these data do not provide evidence that AG specifically reverses CP resistance, but do indicate that AG produces a small potentiation of CP toxicity.

**Studies on cross-resistance patterns and chromosome changes in cisplatin-resistant human bladder and testicular cell lines**

M.C. Walker, J.M. Parrington, L.F. West & J.R.W. Masters

*Urological Oncology Unit, St Paul's Hospital, 24 Endell St, London WC2; and MRC Human Biochemical Genetics Unit, UCL, Stephenson Way, London NW1, UK.*

Cisplatin is the most active single agent in the treatment of advanced testicular germ cell tumours and transitional cell carcinomas of the bladder. However, development of resistance to this drug is a major cause of treatment failure.

Cisplatin-resistant cell lines, designated SuSa-CP and RT112-CP, were derived *in vitro* from a human testicular tumour cell line (SuSa) and a bladder carcinoma cell line (RT112) by continuous exposure to cisplatin. Patterns of cross-resistance to other agents commonly used in treatment of bladder and testis tumours were investigated. The responses of cells to a range of concentrations of cisplatin, carboplatin, VP-16, methotrexate (MTX), Adriamycin (doxorubicin) and bleomycin were compared using a tetrazolium dye (MTT) assay. Approximate levels of cross-resistance are shown in the table; figures are $IC_{50}$ of resistant line/$IC_{50}$ of parental line.

| | *Drugs* | | | | | |
|---|---|---|---|---|---|---|
| *Cell line* | *Cis-platin* | *Carbo-platin* | *MTX* | *VP-16* | *Adria-mycin* | *Bleo-mycin* |
| RT112-CP | 10.0 | 9.9 | 6.6 | 1.9 | 1.0 | 2.8 |
| SuSa-CP | 2.1 | 2.0 | 3.0 | 1.8 | 1.0 | 1.2 |

Modal chromosome numbers were obtained from orcein-stained metaphase spreads. These were: RT112 47; RT112-CP 43; SuSa 52; SuSa-CP 53. G-banding revealed major differences between parent and resistant cell lines, including chromosome losses, gains and translocations. However, the pattern of alterations was not consistent between the cell lines.

### Identification of a relationship between glutathione metabolism and sensitivity to doxorubicin or cisplatin in a range of human tumour cell lines

L.K. Hosking, R.D.H. Whelan & B.T. Hill

*Cellular Chemotherapy Laboratory, Imperial Cancer Research Fund, London WC2A 3PX, UK.*

Differences in total glutathione content (GSH), may explain altered drug responses of human tumour cell lines (Bedford *et al., Chemico-Biological Interactions,* 1987, **61,** 1). Therefore total GSH content and associated enzyme activities have been determined in a series of twenty parental and drug-resistant human tumour cell lines derived following fractionated X-irradiation or drug exposure *in vitro*. These cell lines exhibited differential sensitivities to both doxorubicin and cisplatin as judged by colony formation following 24 h drug exposures. Drug concentrations reducing cell survival by fifty percent ranged from 2–60 and 30–450 ng per ml for doxorubicin and cisplatin respectively, while total glutathione levels were between 10–80 nmol per mg of cellular protein. The relationships between GSH content or certain related enzyme activities and drug sensitivities were statistically analysed using linear regression. Correlation coeficient values of $>0.75$ indicated that GSH content may be a determinant of cellular sensitivity to both cisplatin and to doxorubicin. Glutathione reductase (GR) and glutathione peroxidase (GP) activities may influence cellular sensitivity to cisplatin, as suggested by correlation coefficients $>0.6$, but no correlations were identified for glutathione S-transferase. Statistical analysis of the data obtained using parental cell lines only demonstrated a similar pattern of correlations. Glutathione metabolism in human tumour cell lines may therefore influence cellular sensitivity to certain antitumour agents.

### $O^6$-Alkylguanine-DNA alkyltransferase expression and glutathione transferase action in MAC tumours correlate with intrinsic resistance to nitrosoureas and chlorambucil *in vitro*

J.M. Lunn, J. Carmichael, M.C. Bibby, J.A. Double & A.L. Harris

*Cancer Research Unit, University of Newcastle upon Tyne, Medical School, Framlington Place, Newcastle upon Tyne NE2 4HH; and Clinical Oncology Unit, University of Bradford, Bradford, West Yorkshire BD7 1DP, UK.*

The dimethylhydrazine-induced mouse colon adenocarcinomas (MAC tumours) display ranges of responsiveness to commonly used cytotoxic drugs. In order to assess the relevance of two proposed resistance mechanisms, we have measured the levels of $O^6$-alkylguanine-DNA alkyltransferase ($O^6$AT) and glutathione transferase (GSHT) in MAC13, MAC15, MAC16, MAC26 and MAC30T tumours. $O^6$AT was determined by incubating a $^3$H-Me labelled DNA substrate with tumour extracts. GSHT activity was assessed using chlorodinitrobenzene as substrate. Levels of $O^6$AT ranged from $<20\,fmol\,mg^{-1}$ protein (MAC13) to $80\,fmol\,mg^{-1}$ protein (MAC26), increasing levels of $O^6$AT correlating with increasing resistance to MeCCNU. Decreased resistance of MAC26 to TCNU probably reflects improved drug delivery of the more water soluble nitrosourea in this well vascularised tumour. Levels of GSHT were highest in MAC16 tumours, being five times as great as those in MAC15, MAC26 and MAC30T tumours. The relative activities correlated well with the range of resistance shown to chlorambucil. Thus, resistance to nitrosoureas could be related to the removal of $O^6$-alkylguanine lesions from the DNA, while resistance to chlorambucil was more related to detoxification by glutathione transferase.

### $O^6$-Alkylguanine-DNA-alkyltransferase levels in human testis and bladder tumour cell lines

M.C. Walker, J.R.W. Masters & G.P. Margison

*Urological Oncology Unit, St Paul's Hospital, 24 Endell St, London WC2H 9AE; and CRC Department of Carcinogenesis, Paterson Institute for Cancer Research, Manchester M20 9BX, UK.*

The cytotoxic effects of chloroethylnitrosoureas (CNU) are mediated by the formation of interstrand crosslinks that originate from an initial chloroethylation of the $O^6$-position of guanine in DNA. The enzyme $O^6$-alkylguanine-DNA-alkyltransferase (ATase) can repair this monoadduct and hence prevent crosslink formation. Thus cellular sensitivity to CNU appears to be determined by the level of ATase in the cell.

Testicular tumour cells are sensitive to many anticancer agents, including cisplatin (CP) (Walker *et al., JNCI,* 1987, **79,** 213). Preliminary to studying the cytotoxicity of CNU, we measured ATase levels in 5 testis and 5 bladder tumour cell lines and CP-resistant sublines SuSa-CP (testis) and RT112-CP (bladder). ATase was measured in each cell line by incubation of cell extracts with $^3$H-methylnitrosourea-methylated DNA: calculations were based on protein-limiting values and expressed as $fmol\,mg^{-1}$ total protein.

| Cell line testis | HL | GH | 1618K |
|---|---|---|---|
| ATase (fmol $mg^{-1}$) | 121 | 81 | 230 |
| *Cell line, bladder* | *T24* | *HT1197* | *HT1376* |
| ATase (fmol $mg^{-1}$) | 718 | 20 | 520 |

| Cell line testis | GCT27 | SuSa | SuSa-CP |
|---|---|---|---|
| ATase (fmol $mg^{-1}$) | 4 | 259 | 450 |
| *Cell line, bladder* | *RT4* | *RT112* | *RT112-CP* |
| ATase (fmol $mg^{-1}$) | 278 | 396 | 450 |

Both testis and bladder cell lines showed a wide range of ATase levels. Interestingly, both of the CP resistant cell lines had higher ATase levels than their corresponding parent line. Further studies are in progress to measure the sensitivity of the cell lines which display widest differences in ATase levels to a CNU and to the monofunctional agent methylnitrosourea.

### Cross-resistance studies with quinazoline inhibitors of thymidylate synthase (TS) against two methotrexate (MTX)-resistant sublines of the L1210

J.A.M. Bishop, A.L. Jackman, L.R. Hughes & A.H. Calvert

*Institute Cancer Research, Sutton, Surrey; and ICI Pharmaceuticals PLC, Macclesfield, Cheshire, UK.*

A number of more water soluble, less toxic analogues of $N^{10}$-propargyl-5,8-dideazafolic acid (CB 3717) have been identified as potent inhibitors of cell growth. Many of these compounds are thought to be metabolised intracellularly to polyglutamated forms that are well retained by cells. However, little is known about the mechanism by which these quinazolines enter cells. We describe here, cross-resistance studies using two MTX-resistant sublines of the L1210. The first of these, the L1210:1565 is a reduced folate transport-deficient mutant which expresses the same level of dihydrofolate reductase (DHFR) as the wild type. The second, the L1210:R71 over-produces DHFR by 230 fold.

| | $IC_{50}$ ($\mu M$) | | |
|---|---|---|---|
| | *L1210* | *1565* | *R71* |
| Methotrexate | 0.026 | 3.6 | 630 |
| Trimetrexate | 0.010 | 0.034 | – |
| CB3717 | 3.4 | 27.0 | 10 |
| 2-desamino CB3717 | 0.4 | 28.0 | 17 |
| 2-desamino-2-methyl CB3717 | 0.085 | 53.0 | 4 |

It is clear that removal or replacement of the 2-$NH_2$ function of CB 3717 can have a profound effect on the ability of the molecule to utilise the reduced folate/MTX carrier, increasing the extent to which these analogues are able to utilise this pathway. Comparing the results of the two MTX-resistant lines, we may expect that these compounds will circumvent resistance resulting from DHFR amplification, but not that due to deletion of the reduced folate carrier.

### Comparison of two methods for the study of growth and drug sensitivity of small cell lung cancer cell lines

S. Bicknell, R. Milroy, J.A. Plumb, S. Banham & S.B. Kaye

*CRC Department of Medical Oncology, University of Glasgow and Department of Respiratory Medicine, Royal Infirmary, Glasgow, UK.*

As part of our studies of drug resistance in small cell lung cancer we have established 8 cell-lines from patient biopsies. All grow as floating aggregates in suspension. Estimation of growth rates and chemosensitivity with such cell lines presents major problems since disaggregation frequently leads to loss of cell viability. We have previously reported a modification of a tetrazolium based chemosensitivity assay (*Br. J. Cancer*, 1988, **58**, 231) for use with such lines. We have now compared this assay with the results obtained by the use of spheroid growth delay technology. Individual aggregates were plated out in 24-well plates and growth and response to cytotoxic drugs were monitored by the use of a television camera linked to a microscope and a computer. Although a spectrum of volume doubling times was obtained, this approach was slow and was only reproducible for those lines which grew as tight aggregates comparable to man-made spheroids. In contrast, the tetrazolium based assay was applicable to cell lines with a variety of growth characteristics giving reproducible estimates of doubling times (4–7 days) and drug sensitivity ($ID_{50}$ 36–1050 nM).

Although both techniques allow study of the intact aggregate the tetrazolium based assay is more suitable for laboratory studies of drug resistance in small-cell lung cancer. However, since the spheroid growth delay technique allows serial measurements of individual aggregates it can be used for more detailed studies such as the effect of aggregate size on the apparent chemosensitivity of a cell line.

### Short-term culture of cells from renal cell carcinomas to determine their chemosensitivity

M.O. Symes, T. Lai, B.R. Stonebridge & P.J.B. Smith

*University Departments of Surgery and Computer Science, Bristol Royal Infirmary, Bristol BS2 8HW, UK.*

In 29 cases tumour cells were separated from enzymatically disaggregated renal cell carcinoma tissue by centrifugation of the resulting mixed cell suspension, on a continuous density gradient of Nycodenz (Nycomed AS, Oslo). When the tumour cell band at the top of the column was examined in a haemocytometer counting chamber, only carcinoma cells were seen.

Tumour cells from 29 renal cell carcinomas were exposed *in vitro* to increasing concentrations of Medroxyprogesterone acetate (MPA) or one of several cytotoxic agents for 24 hours. Thereafter the uptake of $^{75}$Selenomethionine ($^{75}$SeM) over 48 hours was compared to that of tumour cells not exposed to a drug.

Using drug concentrations of 1 $\mu g\,ml^{-1}$, MPA produced >50% inhibition of $^{75}$SeM uptake in 5 of 24 cases. The comparable proportion for Doxorubicin was 4 of 5 and for Mitozantrone 10 of 18. Methotrexate and Vinblastine were much less effective. In 3 of 4 cases where >50% inhibition was obtained with Doxorubicin and in 8 of 10 with Mitozantrone the carcinoma had spread beyond the kidney. Thus drug sensitivity may be associated with a high mitotic rate.

In the 10 cases where $^{75}$SeM uptake was inhibited by Mitozantrone the mean drug concentration giving 50% inhibition was $0.51 \pm 0.18$ (s.d.) $\mu g\,ml^{-1}$. The mean plasma concentration time product as measured by HPLC following i.v. injection of 12 mg m$^{-2}$ Mitrozantrone was 0.3 $\mu$g ml$^{-1}$ (Alberts *et al.*, Investigational New Drugs, 1985, **3**, 101). This figure was significantly greater when determined using $^{14}$C-labelled Mitozantrone, and the ratio of whole marrow to plasma concentration was 8:1, whilst 15% of the total administered drug was recovered from body organs after 35 days. Thus reduced uptake of $^{75}$SeM following exposure of tumour cells to 1 $\mu$g ml$^{-1}$ of Mitozantrone for 24 hours may predict clinical responsiveness to this drug.

**Reversal of resistance in the breast cancer cell-line MCF-7/Adr$^{R}$ was most effective with the modulating agent quinidine**

S. Stallard & S.B. Kaye

*CRC Department of Medical Oncology, University of Glasgow, UK.*

The resistant breast cancer cell line MCF-7/Adr$^{R}$ provides a model of drug resistance *in vitro*. It is also a potential model to study means of overcoming this resistance. This cell line is known to overexpress the *mdr1* gene product, the *p*-glycoprotein. Resistance to adriamycin therefore could potentially be reversed by agents known to interfere with the putative *p*-glycoprotein drug efflux system.

In this study five such resistance modifiers have been compared.

The effectiveness of verapamil, D-verapamil, quinidine, bepridil and nifedipine at reversing drug resistance in the MCF-7/Adr$^{R}$ line and the parent MCF-7 line were measured by the MTT assay.

At a concentration of 6.6 $\mu$M for each agent, quinidine increased the sensitivity of the MCF-7/Adr$^{R}$ by 10-fold, verapamil by 6.8-fold, D-verapamil by 5.7-fold, bepridil by 4.3-fold and nifedipine by 5-fold.

None of the agents influenced the sensitivity of the parent MCF-7 line. Quinidine was therefore the most effective agent for reversing drug resistance in the MCF-7/Adr$^{R}$ breast cancer line. Its modulating activity started at a concentration of 4 $\mu$M, and was maximal at 7 $\mu$M. Those concentrations are easily achievable clinically, in comparison to other agents, such as verapamil. Quinidine therefore is an attractive candidate for further clinical study.

**A comparison of ($^{75}$Se)-selenomethionine ($^{75}$SeM) incorporation, protein content and colony inhibition as methods for measuring the efficacy of anti-cancer drugs**

A.P. Moran, T. Lai & M.O. Symes

*Department of Surgery, University of Bristol, UK.*

The anti-tumour action of 5-fluorouracil (5-FU) on cells from the colorectal carcinoma line H-29 was assessed in 3 ways. First the uptake of $^{75}$SeM by $10^5$ tumour cells over 48 hours, following exposure to increasing concentrations of drug for 48 hours was measured. Second the protein content of $10^5$ tumour cells was measured following 48 hours exposure to drug. Third following culture with drug for 48 hours 600 tumour cells were plated and the number of tumour cell colonies formed was counted after 12 days of culture. Results were expressed as percentage inhibition compared to tumour cells not exposed to 5-FU. Seven experiments were performed and the mean ±s.d. for tumour cell inhibition measured by the 3 methods is shown in the table.

| *5-FU concentration ($\mu$g ml$^{-1}$)* | *$^{75}$SeM uptake* | *Protein content* | *Colony inhibition* |
|---|---|---|---|
| 100 | 47.4±6.0 | 91.9± 2.4 | 100 ± 0 |
| 10 | 25.8±7.0 | 83.4± 6.2 | 100 ± 0 |
| 1 | 17.3±5.7 | 49.3±18.1 | 61.0±29.6 |
| 0.1 | 7.8±3.7 | 6.4± 6.3 | – |

Estimation of protein content and of colony inhibition gave higher values for inhibition than $^{75}$SeM uptake. As 5-FU has a greater effect on DNA than on RNA synthesis, and as $^{75}$SeM uptake measures the latter, it is concluded that all 3 methods give useful results. In 2 experiments the effect of mitozantrone was compared by $^{75}$SeM uptake and colony inhibition. Both methods gave similar results at drug concentrations of 10 and 5 $\mu$g ml$^{-1}$, but colony inhibition was greater at 1 and 0.1 $\mu$g/ml.

**Marked enhancement of base analogue cytotoxicity by 3-aminobenzamide; correlation with increased phosphoribosylpyrophosphate levels and augmented base salvage**

E. Watson, K. Moses & B.W. Durkacz

*Cancer Research Unit, Medical School, University of Newcastle upon Tyne, Newcastle upon Tyne NE2 4HH, UK.*

3-Aminobenzamide (3AB) is presumed to inhibit DNA excision repair via an inhibition of ADP-ribosyltransferase (ADPRT) activity. Here we report that 3AB enhances base analogue cytotoxicity by mechanisms other than an inhibition of ADPRT or DNA repair.

Coincubation of CHO-K1 cells with non-toxic concentrations of 3AB (3 mM) potentiates the cytotoxicity of 6-mercaptopurine with a dose enhancement factor (DEF) at 10% survival of 30. Whereas treatment of cells with MP or 3AB alone has no effect on phosphoribosylpyrophosphate (PRPP) levels, coincubation results in a 30-fold increase between 0–1.5 $\mu$M MP, followed by a decline to basal levels at higher doses (2–10 $\mu$M MP). Methotrexate (MTX) enhances MP cytotoxicity by increasing PRPP levels, but the mechanism is distinct from that of 3AB since MTX alone increases PRPP levels. We suggest that 3AB enhances MP cytotoxicity by augmenting salvage via the enzyme hypoxanthine-guanine phosphoribosyl transferase, for which PRPP is a rate limiting substrate. 3AB also enhances 5-fluorouracil (FU) cytotoxicity with a DEF at 10% survival of 2. 3AB treatment causes a 2-fold increase in levels of FU in RNA, but has no effect on levels of FU in DNA. This observation is compatible with increased FU conversion to FUMP resulting from an increase in PRPP levels. However, we observe that 3AB inhibits nucleoside transport and we cannot exclude an inhibition of FUdr efflux by 3AB as a mechanism of potentiation. The reason for the sharp rise and fall in PRPP levels in cells treated with MP and 3AB may reflect fluctuations in levels of MP metabolites, which in turn alter the flux through purine *de novo* and salvage pathways, both of which utilise PRPP. These data demonstrate novel metabolic effects of 3AB, independent of ADPRT inhibition.

**The role of anthrapyrazole–iron complexes in hydroxyl radical formation DNA strand scission and cytotoxicity**

M.A. Graham, D.R. Newell, L.H. Patterson, C. Qualmann, B.K. Sinha & C.E. Myers

*Drug Development Section, Institute Cancer Research, Sutton, Surrey, UK; School of Pharmacy, Leicester Polytechnic, Leicestershire, UK; and Biochemical Pharmacology Section, NCI, Bethesda, MD, USA.*

The anthrapyrazoles (APZ) are anthraquinoneimines, which possess certain structural features common to the anthraquinone mitozantrone (MZT) and the anthracycline doxorubicin (DOX). Iron chelation by DOX and MZT is well characterised and DOX–iron chelates have been shown to catalyse OH mediated DNA strand breakage. By analogy with DOX, A-ring hydroxylated APZs might also chelate iron. Hence, this study investigates the relationship between iron binding, OH radical formation, DNA strand scission and cytotoxicity with four APZs which differ in the A-ring hydroxyl substitution. *In vitro* cytotoxicity tests against a CCRF-CEM cell line, ranked the four analogues in order of potency with IC50 values of, 7-OH (CI-941) ($8\times10^{-9}$M) > 10-OH and 7, 10 unsub.($2\times10^{-7}$M) > 7, 10-OH ($1\times10^{-6}$M). The latter result is probably an under estimate due to the chemical instability of the 7, 10-OH APZ which has a tendancy to form an A-ring quinone. Fe(III) binding (as measured by difference vis-spectroscopy) showed that the 7, 10-OH and 10-OH APZ readily chelated iron with an optimal drug:Fe ratio of 1:2. In contrast, the 7-OH and the 7, 10 unsub. APZ produced relatively weak or no spectral changes. Using ESR spectroscopy, a $H_2O_2$ dependent DMPO spin adduct characteristic of OH was observed in the presence of the 7, 10-OH and 10-OH APZ, but not with 7-OH APZ or the 7, 10 unsub. APZ. These results indicate the importance of the 10-OH group in iron binding and subsequent OH formation. The ability of the APZ-FE chelates to induce DNA strand breaks was studied using pBR322 plasmid DNA in the presence of glutathione as a source of reducing agent. Following a 30 min exposure of DNA at 37°C to the APZ-Fe mixtures (1:2), single strand (SS) and double (DS) breaks in DNA were separated and quantitated using agarose gel electrophoresis. All the APZ/iron mixtures induced SS breaks (30–50% total DNA) at concs > 5 $\mu$M drug, although the effect was no more marked than with iron alone. However, in contrast to iron alone, the simultaneous presence of the APZ and iron resulted in total loss of the supercoiled DNA, indicative of multiple DS breakage, Hence the DNA binding nature of the APZs may facilitate Fe mediated free radical DNA strand scission. The lack of correlation between iron binding/OH radical formation, plasmid DNA strand scission and cytotoxicity indicate that, for CI-941, cytotoxicity is unlikely to be related to the formation of an Fe complex and subsequent OH radical mediated DNA damage.

**Synthesis and some biological properties of quinazoline antifolates variously substituted with fluorine on the benzoyl ring**

T.J. Thornton, A.L. Jackman, B. O'Connor, J. Bishop, L. Hughes & A.H. Calvert

*Drug Development Section, Institute of Cancer Research, Sutton, Surrey; and ICI Pharmaceuticals, Macclesfield, Cheshire, UK.*

Previous studies of the effects of benzoyl ring modifications to $N^{10}$-propargyl-5, 8-dideazafolic acid (CB3717) have included the introduction of 2′-and 3′-Cl and 3′, 5′ di Cl substituents (Jones *et al., J. Med. Chem.*, 1986, **29**, 468). 2′-Cl substitution slightly reduced thymidylate synthase (TS) inhibition, whilst 3′-substitution markedly reduced TS inhibition. Both analogues were less cytotoxic to L1210 cells than CB 3717. The present study concerns the effect of introducing another halogen substituent, fluorine into the benzoyl ring of the 2-desamino-2-methyl analogue of CB 3717 (*Proc. Am. Assoc. Cancer Res.*, 1988, **29**, 1137). Inhibitory effects on isolated L1210 TS and L1210 cells in culture are tabulated. The key step in the preparation of these compounds is the synthesis of the various 4-nitro- or protected 4-aminobenzoic acids appropriately substituted with fluorine.

| Benzoyl ring | | | | *L 1210 TS* | *L 1210 Cells* |
|---|---|---|---|---|---|
| *2′* | *3′* | *5′* | *6′* | *$IC_{50}$ ($\mu M$)* | *$IC_{50}$ ($\mu M$)* |
| H | H | H | H | 0.04 | 0.085 |
| F | H | H | H | 0.02 | 0.027 |
| H | F | H | H | 1.24 | 0.051 |
| F | H | F | H | 0.60 | 0.027 |
| F | H | H | F | 0.08 | 0.061 |
| F | F | H | H | 1.71 | 0.28 |

2′-Monofluoro substitution improves TS inhibition and L1210 cytotoxicity, 3′-Monofluoro substitution reduces TS inhibition by 31-fold, but slightly improves cytotoxicity. Introducing a second F into the ring progressively worsened TS inhibition going from 2′,6′ diF to 2′,5′ diF to 2′,3′ diF although these compounds retained the same level of cytotoxicity. The beneficial influence of the 2′-fluoro-substituent may be due to an interaction between F and the amide H, as evidenced by $^1$H-NMR spectroscopy, causing co-planarity between the benzene ring and the amide residue.

**The synthesis and thymidylate synthase (TS) inhibitory activity of the poly-$\gamma$-glutamyl derivatives of some analogues of $N^{10}$-propargyl-5, 8-dideazafolate (CB3717)**

G. Bisset, K. Pawelczak, A.L. Jackman, E.E. Dix Perkin, A.H. Calvert & L.R. Hughes

*Institute Cancer Research, Sutton, Surrey; and ICI Pharmaceuticals, Alderley Park, Cheshire, UK.*

CB3717 has been shown to be metabolised intracellularly to poly-$\gamma$-glutamate derivatives by the enzyme folypolyglutamate synthetase (FPGS). These metabolites are very much more potent inhibitors of isolated L1210 TS (up to ~150-fold) and are preferentially retained within L1210 cells (Sikora *et al., Biochem. Pharmacol.*, 1988, **37**, 4047). Polyglutamation of folate-based TS inhibitors may therefore enhance their cytotoxicity. The more water-soluble 2-desamino (A) and 2-desamino-2-$CH_3$ CB3717 (B) analogues were found to be less active against TS (8 and 2-fold respectively) but significantly more cytotoxic to L1210 cells (10 and 40-fold) (*Proc. Am. Assoc. Cancer Res.*, 1987, **28**, 1073; 1988, **29**, 1138). Both these analogues were found to be substrates for FPGS. We have synthesised some of the poly-$\gamma$-glutamyl derivatives of (A) and (B). The poly-$\gamma$-glutamyl derivatives of B with a chain length of up to 5 glutamates were synthesised from 2-desamino-2-$CH_3$-$N^{10}$-propargyl-5,8-dideazapteroic acid. The tri and tetraglutamates of A were synthesised from 2-desamino-$N^{10}$-propargyl-5,8-dideazapteroic acid that had been obtained by carboxypeptidase $G_2$ cleavage of A. The TS inhibitory activity of these polyglutamates are shown below.

| Compound | TS Kiapp+s.e. (nM) | Fold increase in TS inhibition |
|---|---|---|
| A | 63.68+2.89 | – |
| A+glu2 | 0.97+0.06 | ×66 |
| A+glu3 | 0.63+0.07 | ×101 |
| B | 31.18+1.92 | – |
| B+glu1 | 1.11+1.92 | ×28 |
| B+glu2 | 0.40+0.03 | ×78 |
| B+glu3 | 0.27+0.03 | ×115 |
| B+glu4 | 0.40+0.03 | ×78 |

The fold increase in TS inhibitory activity per glutamate addition is similar to that previously reported for CB3717. The remarkable increase in the cytotoxicity of A and B over CB3717 is therefore probably due to an increased rate of the intracellular synthesis of polyglutamate metabolites.

**Direct evidence that a free radical mechanism is involved in adriamycin toxicity**

A. Bartoszek, L. McLellan, J.D. Hayes & C.R. Wolf

*ICRF, Lab. of Mol. Pharmacol. Hugh Robson Bldg, George Sq, Edinburgh; and Dept of Clinical Chemistry, Royal Infirmary of Edinburgh, Edinburgh, UK.*

A variety of mechanisms have been proposed for the cytotoxic effects of adriamycin (Adr). However, most of the evidence for these pathways is circumstantial. In this study we have obtained evidence that a free radical mechanism is directly involved in the cytotoxic effects of this compound. Adr can be converted to free radical products by cytochrome P-450 reductase. The MTT assay was used to generate cytotoxicity curves for Adr towards the MCF7 cell line in the presence or absence of the reductase. The presence of this enzyme together with the required cofactor NADPH significantly increased the cytotoxicity of this compound. A 5-fold increase in the cytotoxicity being observed. This effect was dependent on both the dose of Adr and the amount of enzyme added. If the reductase was preincubated with Adr prior to addition to the assay no change in cytotoxicity was seen indicating the involvement of an unstable toxic reactive intermediate in the reaction pathway. In addition, initial studies indicate that the addition of purified glutathione transferases to the MTT assay in the presence of NADPH and the reductase can reduce the cytotoxicity of this compound.

**Uptake, DNA binding and cytotoxicity of diethylaminoalkylaminoanthraquinones in MCF-7 human breast cancer cells**

J.E. Maine, D. Cairns, J.R. Brown & L.H. Patterson

*Dept of Pharmacy, Leicester Polytechnic, Leicester LE1 9BH, UK; and Dept of Pharmaceutical Chemistry, Sunderland Polytechnic, Sunderland SR2 7EE, UK.*

The importance of the DNA binding anthraquinones as antitumour agents is exemplified by the clinical use of doxorubicin and mitozantrone. However, doxorubicin therapy is compromised by a cumulative dose related cardiotoxicity while mitozantrone is indicated only for treatment of advanced breast cancer and acute leukaemias. This has led us to investigate the cytotoxic potential of other DNA-binding anthraquinones of which the 1-, 1,4-, 1,5- and 1,8-diethylaminoethylamino-substituted anthraquinones are described here. The synthesis and DNA binding affinity of these compounds has been previously described (Islam *et al.*, *J. Med. Chem.*, 1985, **28**, 857). Cytotoxicity was measured after 24 h treatment of $10^5$ exponentially growing cells with anthraquinones (0.01–10 μM) and a subsequent 48 h drug-free incubation. To measure drug uptake MCF-7 cells were incubated with the anthraquinones (15 μM) for between 1 and 90 min, the cells were then centrifuged at 12,000 *g* and the supernatant analysed by HPLC using a C6 (Spherisorb) 15 cm column with a acentonitrile (30%): ammonium formate (70%, 0.5 M, pH 4.25) mobile phase. All the anthraquinones were cytotoxic to MCF-7 cells with $ED_{50}$ values as follows: 1,5-AQ (<0.01 μM)>1-AQ (0.01 μM)>doxorubicin (0.04 μM)>1,8-AQ (1 μM)>1,4-AQ (2 μM). The wide range of cytotoxicity observed was not due to differences in cellular uptake which for all compounds appeared to approach steady state after 60 min with the following uptake values: 1-AQ (34%), 1,8-AQ (34%), 1,4-AQ (36%), 1,5-AQ (46%), doxorubicin (47%). The ranking of cytotoxicity does not correlate with DNA binding affinity which is 1,5-AQ>1,4-AQ>1,8-AQ>1-AQ. It is likely therefore that other mechanisms contribute to the cytotoxicity observed. In this respect these compounds have been shown previously to generate reactive oxygen species.

**Increased anabolism of 5-fluorouracil in Walker carcinosarcoma following methotrexate pretreatment, detected by $^{19}F$ MRS**

P. McSheehy, M. Prior & J. Griffiths

*CRC Biomedical Magnetic Resonance Research Group, St George's Hospital Medical School, London SW17 0RE, UK.*

$^{19}F$ magnetic resonance spectroscopy (MRS) can follow tumour 5-fluorouracil (5FU) metabolism to cytotoxic fluoronucleotides (FNuct) non-invasively *in situ* (Stevens *et al.*, *Br. J. Cancer*, 1984, **50**, 113), and we have shown in the Walker carcinosarcoma that the FNuct peak area is related to growth inhibition. 5FU 3 hours after methotrexate (MTX-5FU) gives a superior therapeutic ratio in the Walker tumour compared to both drugs simultaneously, alone or in reverse order (5FU-MTX) (Wayss *et al.*, *Med. Oncol. Tumour Pharmacother.*, 1985, **2**, 27). Our aim was to use MRS to detect increased 5FU metabolism to FNuct following the MTX-5FU schedule and to relate this to cytotoxicity.

For MRS, 6 anaesthetised rats bearing Walker tumours received 5FU (50 mg $kg^{-1}$ i.v.) 3 hours after MTX 50 mg $kg^{-1}$ (test) or 0.9% NaCl (control) i.p. After 60 min the experiment was terminated by excising and freeze-clamping the tumour for analysis by ion-exchange HPLC. For growth studies 10 rats per group received Walker cells on day 0 and on days 1 and 5 received either 0.9% NaCl, 5FU (50 mg $kg^{-1}$), MTX-5FU (50 mg $kg^{-1}$) or 5FU/MTX (50 mg $kg^{-1}$) i.p. Tumour size was assessed on day 8. Results are mean ± SEM.

After 60 min the $^{19}F$ MRS FNuct/5FU peak area ratio in tests was significantly increased: 0.68 ± 0.24 *vs* 3.17 ± 0.97 ($P$=3.1%). The 5FU peak area was unchanged (866 ± 120 *vs* 1022 ± 245) while FNuct was significantly increased (568 ± 217 *vs* 1949 ± 420), $P$=1.5%. HPLC showed 50% of the FNuct in both treatments was FUTP, which was increased 3-fold in tests. MTX-5FU reduced tumour growth ($P$=1%), whereas 5FU alone did not, but it was not significantly different to 5FU-MTX. MRS may have a role in optimising the scheduling of MTX and 5FU combination chemotherapy.

**The effect of cationic DNA affinity binders on the DNA sequence selective alkylation of guanine-N7 positions by nitrogen mustards**

J.A. Hartley, S.M. Forrow & R.L. Souhami

*Dept of Oncology, University College and Middlesex School of Medicine, 91 Riding House Street, London W1P 8BT, UK.*

Large variations in alkylation intensities exist among guanines in a DNA sequence following treatment with chemotherapeutic alkylating agents such as nitrogen mustards and chloroethylnitrosoureas. The majority of compounds react preferentially in runs of guanines which correlates well with the molecular electrostatic potential at the guanine-N7 position imposed by the nearest neighbour base pairs, and suggests that the specific biological effects of such compounds may depend on preferential reaction at GC-rich genomic locations. It is also clear that the substituent attached to the reactive group could impose a distinct sequence preference for reaction.

In order to understand further the structural and electrostatic factors which determine the sequence selectivity of alkylation reactions, the effect of increased ionic strength, the intercalator ethidium bromide, AT-specific minor groove binders netropsin and distamycin A, and the polyamine spermine on guanine-N7 alkylation was investigated using a modification of the guanine-specific chemical cleavage technique for DNA sequencing. Increased ionic strength produced the expected reduction in reaction rates but did not alter the rank order of reactivity of guanines to nitrogen mustards, although the degree of selectivity was altered in some cases depending on the charge on the mustard molecule. Alkylation was also dose-dependently inhibited by all the cationic DNA affinity binders studied. More interestingly, however, the guanine-N7 alkylation pattern was qualitatively altered by pretreatment with ethidium bromide, netropsin and distamycin A. The result differed both with the nitrogen mustard and the cationic agent used. The effect was most striking in the case of netropsin and distamycin A indicating that selective binding to AT sequences in the *minor* groove can have long range effects on the alkylation of DNA in the *major* groove.

**Enhanced 4-hydroperoxycyclophosphamide cytotoxicity and DNA cross-linking following BCy NU-mediated glutathione depletion in human leukaemia cells**

C.M. Chresta, T.R. Crook & R.L. Souhami

*Dept of Oncol., Univ. College & Middlesex School of Medicine, 91 Riding House Street, London W1P 8BT, UK.*

*N,N'*bis (*trans*-4-hydroxycyclohexyl) *N'*-nitrosourea (BCyNU) is a nitrosourea which has carbamoylating but not alkylating activity. It has been demonstrated to carbamoylate and inactivate glutathione reductase thereby reducing the intracellular levels of glutathione (GSH). We have previously shown that buthionine-S,R-sulphoximine -mediated GSH depletion potentiated the cytotoxicity of cyclophosphamide, with a corresponding increase in DNA cross-linking. We have, therefore, investigated the potential interaction between BCyNU and cyclophosphamide. Treatment of K562 human leukaemia cells with 15 $\mu$M BCyNU for one hour resulted in depletion of glutathione to 40% of control values, without significant reduction of cell viability as measured by the MTT assay. Subsequent treatment with 10 $\mu$M 4-hydroperoxycyclophosphamide (4-HC), a self-activating derivative of cyclophosphamide, reduced the level of glutathione to less than 20% of control values. BCyNU pretreatment enhanced the cytotoxicity of 4-HC resulting in a dose modification factor of 2.5. Alkaline elution analysis of cellular DNA demonstrated that the level of interstrand cross-linking was 2-fold higher in GSH depleted cells than in non-depleted cells, in addition the induction of single strand breaks was markedly increased.

These findings demonstrate that BCyNU potentiates the cytotoxicity of 4-HC, and suggest that this is due to the increased formation of DNA interstrand cross-links caused by a reduced intracellular conjugation of drug with glutathione which results in an increased binding of drug to DNA targets.

**Photosensitisation of human leukaemic cells by anthracenedione antitumour agents**

J.A. Hartley, S.M. Forrow, R.L. Souhami, K. Reszka & J.W. Lown

*Dept of Oncology, University College & Middlesex School of Medicine, London; and Dept of Chemistry, University of Alberta, Edmonton, Alberta, Canada.*

While 1,4-diaminosubstituted anthraquinone antitumour agents such as mitoxantrone and ametantrone do not show marked photosensitising capabilities, the structurally related 1,5- and 1,8-diaminosubstituted compounds (AM1 and AM2) efficiently oxidised NADH upon illumination ($\lambda > 475$ nm). Illumination of aerated samples of AM1 and AM2 in the presence of NADH has previously been shown to cause oxygen consumption, formation of superoxide radical and hydrogen peroxide. Similarly these compounds caused formation of DNA single-strand breaks (SSBs) upon exposure to visible light and a correlation was established between extent of DNA damage, oxygen consumption and NADH oxidation.

In the present study AM1 and AM2 were tested for their ability to photosensitise human leukaemic cells in culture. Viability was assessed using the MTT assay and DNA SSBs by alkaline elution. Following a 1 hour exposure to AM2, an $ID_{50}$ value of 53 $\mu$M was obtained in the dark, which was reduced to approximately 2 $\mu$M following illumination for 2 min, a dose of light which was non-toxic to cells in the absence of drug. A shift in cell viability curve was also observed for AM1 but under identical conditions the dose modification was only 10. In contrast, neither ametantrone or mitoxantrone gave a decreased viability upon illumination. No DNA SSBs were detected in unilluminated cells up to 10 $\mu$M drug, and were only seen at the $ID_{50}$ (50 $\mu$M). In contrast, a large amount of SSBs was observed at 2 $\mu$M AM2 following illumination, although light alone produced no damage. Whether such photochemical reactions will contribute to unnecessary phototoxic side-effects, or can be exploited in photodynamic therapy remains to be established.

**A new tumour-associated antigen which is recognised by monoclonal antibody NCRC-37 and expressed on the cells and in the sera of gastrointestinal cancer patients**

L.G. Durrant, E. Jacobs, R. Charnley & R.W. Baldwin

*Cancer Research Campaign Laboratories, University of Nottingham; and Department of Surgery, University Hospital, Nottingham, UK.*

A monoclonal antibody recognising a tumour associated antigen on gastrointestinal cells was produced from fusion of splenocytes from a mouse immunised consecutively with four different colorectal cell lines. The antigen is present in the acini of gastrointestinal epithelial but is expressed by the cells and in the pseudoacini of gastrointestinal tumours. It is not expressed by haematopoietic cells or on lung, kidney, liver, brain, heart, spleen, testicular or skin tissues.

The epitope is expressed on a high molecular weight (>200 kDa) glycoprotein and may also be expressed on other antigens (experiments in progress). It is not found on carcinoembryonic antigen. However the epitope is found on an antigen(s) secreted into the sera of patients with gastrointestinal cancer.

| | *Percentage of samples with following (antigen(s) (units ml$^{-1}$)* | | | |
|---|---|---|---|---|
| | *0–10* | *11–20* | *21–30* | *31–130* |
| Colorectal cancer | | | | |
| Normal | 60 | 24 | 16 | 0 |
| Primary tumours | 27 | 32 | 14 | 27 |
| Recurrent tumours | 0 | 33 | 0 | 67 |
| Metastases | 7 | 7 | 13 | 73 |
| Gastric cancer | | | | |
| Normal | 0 | 33 | 67 | 0 |
| Gastric tumours | 6 | 6 | 54 | 34 |

There was no correlation between the antigens detected by antibody NCRC-37 and CEA or CA19-9 antigens.

NCRC-37 detects a new tumour associated antigen which may complement postoperative CEA monitoring for detection of recurrent disease or response to therapy.

**Development of an ELISA to detect early local relapse of colorectal cancer**

L.G. Durrant, N.C. Armitage & R.W. Baldwin

*Cancer Research Campaign Laboratories and Department of Surgery, University Hospital, Nottingham, UK.*

There is considerable interest in non-invasive techniques for identification of patients with recurrent disease following resection of primary colorectal tumours. At present CEA is used in postoperative monitoring, but computerised tomography showed identifiable recurrence 7.5 months before an increase in CEA was seen. There is a need for indentification of a new tumour associated antigen secreted by a large percentage of early recurrent tumours to complement CEA monitoring of this disease.

The Y haptenic blood group is expressed by the cells of malignant gastrointestinal tumours and therefore assays to detect serum antigens bearing this hapten have been developed. The specificity of the assays was between 88–93% and the sensitivity in detecting extensive disease was between 24 and 33%. However, up to 67% of patients with local or abdominal recurrent disease secreted antigens expressing the Y hapten whereas only 30% of patients with overt hepatic metastases secrete a similar antigen. There was no correlation with expression of CEA or CA19-9 antigens. Recognition of antigens bearing the Y hapten may be useful in detecting early local relapse of colorectal cancer.

**The following companies have kindly contributed towards the costs of this meeting:** Abbot Laboratories, Amersham International, Astra Alab AB, Becton Dickinson UK, Beecham Pharmaceuticals, Bio-Rad Laboratories, Boehringer Ingelheim, Boehringer Mannheim, Bristol Myers, Canberra Packard, Farmitalia Carlo Erba, Gibco BRL, Glaxo, Arnold Horwell, ICI, ICN Biochemicals, Kirby Warrick, Lederle Laboratories, Northumbria Biologicals, Olympus Optica Co., Perkin Elmer, Roche, Sera Lab, Vector Laboratories, Wellcome Research Laboratories.